# Content-aware frame interpolation (CAFI): deep learning-based temporal super-resolution for fast bioimaging

**DOI:** 10.1038/s41592-023-02138-w

**Published:** 2024-01-18

**Authors:** Martin Priessner, David C. A. Gaboriau, Arlo Sheridan, Tchern Lenn, Carlos Garzon-Coral, Alexander R. Dunn, Jonathan R. Chubb, Aidan M. Tousley, Robbie G. Majzner, Uri Manor, Ramon Vilar, Romain F. Laine

**Affiliations:** 1 Department of Chemistry, Imperial College London, London, UK; 2 Centre of Excellence in Neurotechnology, Imperial College London, London, UK; 3 Facility for Imaging by Light Microscopy, NHLI, Imperial College London, London, UK; 4 Waitt Advanced Biophotonics Center, Salk Institute for Biological Studies, La Jolla, CA USA; 5 CRUK City of London Centre, UCL Cancer Institute, London, UK; 6 Department of Pediatrics, Stanford University School of Medicine, Stanford, CA USA; 7 Institute of Human Biology, Roche Pharma Research & Early Development, Roche Innovation Center Basel, Basel, Switzerland; 8 Laboratory for Molecular Cell Biology, University College London, London, UK; 9 Department of Chemical Engineering, Stanford University, Stanford, CA USA; 10 Department of Cell & Developmental Biology, University of California, San Diego, CA USA; 11 Present Address: Micrographia Bio, Translation and Innovation Hub, London, UK

**Keywords:** Machine learning, Imaging, Software

## Abstract

The development of high-resolution microscopes has made it possible to investigate cellular processes in 3D and over time. However, observing fast cellular dynamics remains challenging because of photobleaching and phototoxicity. Here we report the implementation of two content-aware frame interpolation (CAFI) deep learning networks, Zooming SlowMo and Depth-Aware Video Frame Interpolation, that are highly suited for accurately predicting images in between image pairs, therefore improving the temporal resolution of image series post-acquisition. We show that CAFI is capable of understanding the motion context of biological structures and can perform better than standard interpolation methods. We benchmark CAFI’s performance on 12 different datasets, obtained from four different microscopy modalities, and demonstrate its capabilities for single-particle tracking and nuclear segmentation. CAFI potentially allows for reduced light exposure and phototoxicity on the sample for improved long-term live-cell imaging. The models and the training and testing data are available via the ZeroCostDL4Mic platform.

## Abstract (web summary)

Content-aware frame interpolation (CAFI) improves the temporal resolution in time-lapse imaging by accurately predicting images in between image pairs. By allowing fewer frames to be imaged, CAFI also enables gentler live-cell imaging.

## Main

Live-cell imaging is a powerful tool to study dynamic cellular processes by capturing spatiotemporal organization of biological micro-environments. For this, the imaging speed of a microscopy acquisition needs to be sufficiently high to observe cellular processes and dynamic patterns accurately, which sometimes compromises the signal-to-noise ratio (SNR), resolution of the acquired images and/or viability of the sample. Improving the SNR or increasing the dimensionality of the data recording (for example, 4D (3D + *t*) acquisitions) provides better context but slows down the recording speed, making it more difficult, if not impossible, to capture and understand dynamic processes.

**Fig. 1:**
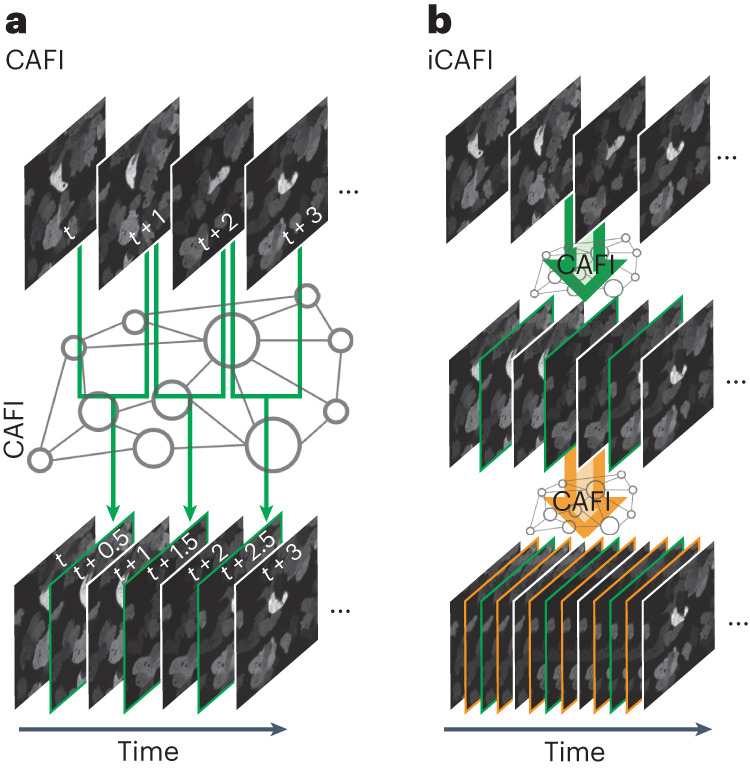
Schematic representation of CAFI. **a**, CAFI can interpolate images between consecutive frames in a time-course dataset, therefore doubling the resulting temporal frequency. **b**, iCAFI allows for further improvement of the temporal resolution by repeatedly applying the network prediction.

Although classical mathematical interpolation techniques such as simple frame duplication (NONE) or bilinear (BIL) or bicubic (BIC) interpolation can artificially increase the temporal image density as a post-acquisition step, those methods do not provide more information about the sample dynamics. A smarter interpolation tool would allow for a time-course acquisition to be resampled with higher temporal sampling in a fashion that would be content aware^1^ with respect to the dynamics observed and would therefore provide accurate predictions of the missing temporal frames, thereby enabling effectively higher temporal resolution imaging (Fig. 1a). Such approaches would effectively lower the illumination dose on the specimen, reducing phototoxicity and photobleaching and allowing longer recordings without compromising cell health or data quality. Therefore, developing computational tools that increase the temporal image resolution in a content-aware fashion have the potential to be transformative and can push the capabilities of any speed-limited microscopy modality.

Deep learning (DL) algorithms have been used for microscopy image post-processing for several years and have transformed the analysis and interpretation of imaging data^1–3^. This has led to several breakthroughs for applications in the field of cellular imaging allowing researchers to carry out previously unachievable experiments. For example, DL strategies have been successfully used to improve low-SNR images^1,4,5^, and enhance microscopy image resolution in both the lateral and axial dimensions^6–12^.

The computer vision subfield of video frame interpolation (VFI) developed several DL frameworks for increasing the frame rate of videos to produce slow motion movies^13,14^. The content-aware frame interpolation (CAFI) task is not trivial due to the diversity of the optical flow of the moving objects, and frame interpolation neural networks are known to sometimes produce inaccurate predictions or artifacts^14,15^ that could mislead researchers to draw faulty conclusions on biological processes. Despite the great potential of such tools, no fully validated and well-performing CAFI implementations are currently available to the scientific community, let alone as easy-to-use software solutions.

In this Article, we present two implementations of state-of-the-art CAFI networks, Zooming SlowMo (ZS)^16,17^ and Depth-Aware Video Frame Interpolation (DAIN)^15^ for smart interpolation of microscopy video data. Both networks have shown competitive performance in various benchmark studies against other VFI networks without producing substantial visual artifacts on videos^14–17^.

The DAIN network has demonstrated very strong abilities in several interpolation tasks, was used in many comparison studies and has been cited 235 times^18–23^ so far and provided a good code base for reimplementation. The network has been ranked in the top four networks in the Advances in Image Manipulation (AIM) 2019 challenge^14^, in 2020 it was still among the top five performing networks in the VFI Vimeo90K benchmark^24^, and even in 2022 it performed highly competitively against other state-of-the-art networks^18^. Furthermore, DAIN has been applied for X-ray cardio-angiography, reducing the radiation exposure for generating high-frame rate videos^25^.

The ZS network also displayed competitive state-of-the-art performance in a spatiotemporal interpolation comparison study^26^ and was successfully used as a comparison network for investigating improvements of low-quality infrared videos^27^. DAIN and ZS were also directly compared in an exhaustive space–time interpolation study (comparing DAIN + EDVR^28^ against ZS)^16^. In this study ZS performed better in the one-step inference of spatiotemporal upsampling than the two step approach with the DAIN + EDVR networks. Therefore we chose DAIN and ZS also for this study to apply their temporal upsampling capabilities on microscopy images. Here we demonstrate and compare their abilities to increase the temporal image frequency for a range of microscopy scenarios, perform extensive tracking analysis benchmarks on simulated and real-life experimental data and demonstrate the network’s ability to improve segmentation results on labeled nuclei, therefore validating the potentials of the approach.

Surprisingly, even the pretrained models of these networks obtained from natural scenes (moving cars and so on) perform better than conventional NONE, BIC and BIL interpolation. Importantly, we show that fine-tuning these models on appropriate microscopy datasets further improves the quality of the CAFI output depending on the quality and amount of data available. Additionally, we quantitatively demonstrate the improvements achieved by CAFI for the task of single-particle tracking (SPT) using both simulated and an experimental live-cell lysosomal dynamics dataset. Tracking of both datasets showed clear performance improvements after CAFI on several quantitative evaluation criteria.

Furthermore in a tracking experiment on simulated moving particles with increased noise levels the neural network interpolations performed better than classical BIL interpolation, but with increased noise levels the quality of the tracking results dropped more strongly than for the classical interpolation technique. In an image segmentation experiment of stained nuclei we also show that DAIN and ZS both increased the segmentation results over classical interpolation techniques, where the DAIN network gave the best results on this task.

Overall, we show the improved performance of CAFI over NONE and BIL image interpolation method on 12 different datasets of four microscopy modalities (point-scanning confocal, spinning-disk confocal, lattice light sheet and confocal brightfield microscopy), suggesting that content awareness can embed relevant information that was not present in the raw dataset. We also demonstrate that CAFI can be used iteratively (iCAFI; Fig. 1b) to increase the restored temporal sampling even further, to 16-fold in the simple case of moving particles. Also, we show that CAFI can be used to improve both the temporal and axial sampling of five multidimensional datasets (3D + *t*) (Extended Data Fig. 3). These datasets include cell dynamics of different cell lines (CAR-T, MDCK and SH-SY5Y cells and two datasets of *Caenorhabditis elegans* embryos labeled with α-tubulin and green fluorescent protein (GFP), respectively). The pretrained networks of both networks consistently performed better than classical interpolation techniques based on image quality assessment. The performance improvement of the fine-tuned networks was however dependent on the quality and amount and quality of the training data available and therefore needs to be evaluated before use by thorough downstream analysis.

We provide the two CAFI network implementations, the corresponding data and pretrained models as part of the ZeroCostDL4Mic platform^29^, making CAFI easily available to the wider scientific community both for running predictions and for fine-tuning the networks.

## Results

### CAFI exceeds classical methods in temporal interpolation

**Fig. 2:**
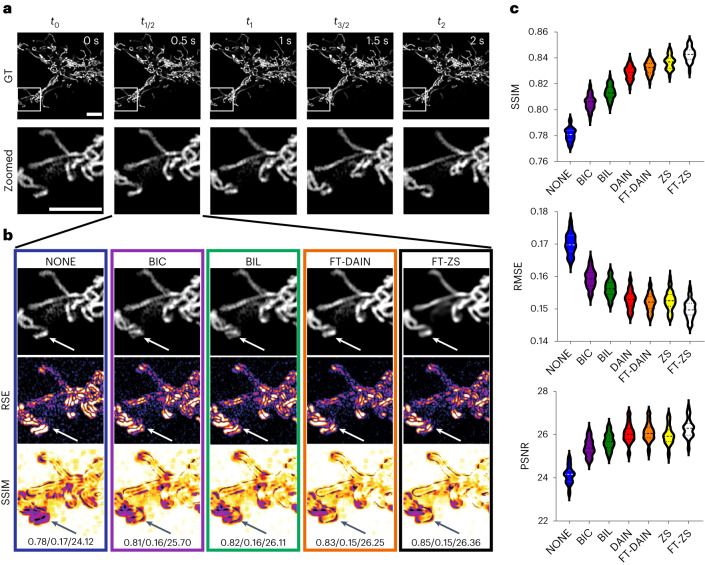
CAFI enables the recovery of fast mitochondrial dynamics. **a**, Ground truth (GT) image sequence example (top) with zoomed-in sections (bottom) of labeled mitochondria branches of U2OS cells (scale bars, 5 μm; timestamp in top right corner of GT images). **b**, Interpolated frames (top), RSE maps (middle) and SSIM maps (bottom) with white/black arrows highlighting areas of fast movement; SSIM/RMSE/PSNR displayed below. **c**, SSIM, RMSE and PSNR metric results compared for each interpolation method including best-performing fine-tuned DAIN (FT-DAIN) and ZS (FT-ZS) networks (*n* = 48 interpolated image frames). Data from Fang et al.^30^. Source data

To investigate the potential for CAFI to accurately predict time frames, we initially tested the two CAFI networks (DAIN and ZS) on a publicly available mitochondrial dynamics dataset^30^. The network predictions were compared with classical interpolation techniques such as BIC, BIL interpolation and NONE. The quality of the interpolated images was evaluated using three common objective pixel-based quality metrics: structural similarity (SSIM), root-mean-square error (RMSE) and peak signal-to-noise ratio (PSNR)^1,31^. SSIM measures the similarity between two images by comparing luminance, contrast and structure, making it a robust and comprehensive metric for evaluating image quality. RMSE quantifies the differences between interpolated and ground truth images on a pixel-by-pixel basis, providing a measure of the overall error magnitude. PSNR, on the other hand, gauges the ratio between the maximum possible signal power and the power of the noise, offering an indication of the fidelity of the reconstructed image compared with the original. These metrics have been widely used in the evaluation of image reconstruction methods, making them suitable for assessing the performance of CAFI in our study. To objectively compare ZS with DAIN, the ZS network architecture, originally implemented with 4× pixel upsampling^16^, was modified to only perform the interpolation without any upsampling. We implemented both networks as ZeroCostDL4Mic notebooks^29^ and trained the models using the Vimeo90K-septuplet video dataset^24^ (for more details on training, see Extended Data Fig. 2). We hypothesize that training the networks on publicly available and large video datasets, even if unrelated to microscopy, teaches them to recognize general movement dynamics in image sequences that may be useful for live-cell dynamics. For the DAIN network, a pretrained model using this dataset is already available^15^. A detailed description of the training fine-tuning process, including the dimensions and size of the training data, can be found in Extended Data Fig. 2. Without any further fine-tuning on microscopy images, both networks already performed better than the classical NONE, BIL and BIC interpolation techniques (Fig. 2b), demonstrating general dynamics can be learnt from natural scenes and applied to microscopy datasets. After fine-tuning of ZS (FT-ZS) and DAIN (FT-DAIN) models on a subset of the mitochondria dynamics data, the performance of both models further improved (see quality metrics for FT-DAIN and FT-ZS in Fig. 2c). Based on the quality metrics, FT-ZS best captured movement patterns of the mitochondria branches, followed by FT-DAIN and both networks could interpolate movement patterns with greater precision than any classical technique. This experiment was performed on two independent mitochondrial image sequences (Fig. 2, Extended Data Figs. 4 and 5 and Supplementary Video 1). As expected, the simple frame duplication NONE was the worst-performing method as it does not blend or add any new information into the interpolated image frame. Furthermore, we noticed that BIL performed better than BIC, which created slightly stronger smeared interpolated frames than BIL leading to the weaker performance. Therefore, we used only NONE and BIL interpolation for further image quality comparison studies.

### Predicting simulated particle motion with CAFI

Lysosomal behavior is a highly dynamic phenomenon involved in important cellular processes such as degradation and repair mechanisms^32^. To study its dynamics, the organelles need to be tracked at speed over long periods of time, which can be a challenge for conventional imaging approaches. To investigate whether CAFI can improve the tracking performance of particle motion such as lysosome dynamics, we initially tested the different interpolation techniques on simulated fluorescent particles moving with a range of motion, as was done for the 2014 International Symposium on Biomedical Imaging (ISBI) particle tracking challenge^33,34^. The simulated dataset gave us the opportunity to control all parameters of the particle properties such as size, velocity and contribution of random and directed motion as well as Brownian motion to successfully mimic lysosomal dynamics. Furthermore, the simulated dataset provided the ground truth of the particle locations allowing for easy comparison of the tracking results after CAFI. The full set of parameters used for data generation is given in Supplementary Table 1 and a detailed explanation on the parameters can be found in Methods.

First, we evaluated the ability of the networks to cope with increasing particle velocities. For this, ground truth datasets were generated by temporally downsampling a densely generated simulated dataset. An increasing number of frames were removed to simulate increasing particle velocities. The missing frames were subsequently used as ground truth for image quality evaluation. These same missing frames were predicted with NONE, BIL interpolation and DAIN and ZS after fine-tuning the models (see Extended Data Fig. 2 for further details on training, see visual illustration of downsampling methodology in Supplementary Fig. 1 and for more details on how the data was generated see Methods). The different particle velocities are labeled from V2 to V13 indicating the maximum number of pixels that a particle with directed linear motion could travel from one image to the next (considering that particles not always traveled in each frame).

**Fig. 3:**
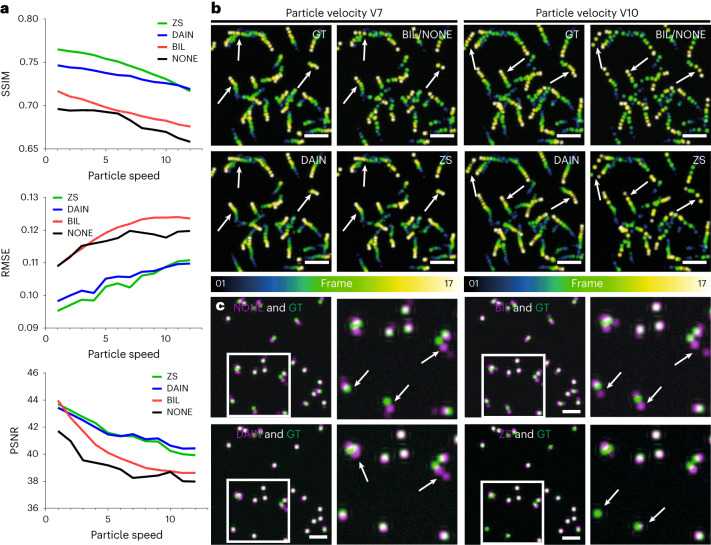
Quantitative assessment of CAFI performance in predicting particle motion using simulated data. **a**, Image quality metrics comparison (SSIM, RMSE and PSNR) of simulated dataset interpolation with NONE, BIL, DAIN and ZS for increasing particle velocities with particle diameters of 15 pixels. These values represent the averaged metrics over the entire dataset, which comprises 17 interpolated image slices. **b**, Temporal color-coded overlaid projection of image sequences of the different interpolation methods for the particle velocities V7 (left) and V10 (right); white arrows highlight regions of interest for comparing interpolation performance (scale bar, 100 pixels; *n* = 17 frames). NONE is not displayed because of visual equivalence to BIL. **c**, Representative artifacts observed from predictions of NONE (SSIM 0.344, RMSE 0.203, PSNR 21.69), BIL (SSIM 0.375, RMSE 0.200, PSNR 22.58), DAIN (SSIM 0.435, RMSE 0.187, PSNR 23.82) and ZS (SSIM 0.455, RMSE 0.184, PSNR 23.57) (in magenta) overlaid with ground truth (in green) of the simulated dataset at particle velocity V7. White arrows highlight regions of interest where different techniques make mistakes (scale bars, 50 pixels). Source data

We observed that the interpolation quality of all interpolation techniques decreased with higher particle velocities (Fig. 3a). Surprisingly, NONE performed better than BIL interpolation for these tracking results, which can be explained by the fact that simple frame duplication does not introduce any artifacts to the image sequence, which BIL does by blending two images together to generate the intermediate one. CAFI performed mostly better than NONE and BIL interpolation and ZS tended to perform better than DAIN for low velocities. However, ZS interpolation quality was more substantially affected by increasing velocities than DAIN. This can also be seen qualitatively in Fig. 3b and Supplementary Fig. 2. Both networks performed very well on particles with slow to medium velocities (see white arrows in Fig. 3b, left) but at higher velocities DAIN performed better than ZS (see white arrows highlighting error regions in Fig. 3b, right). When multiple particles are in close proximity and moving in different directions or when particles move too quickly, DAIN erroneously interpolates the signal in multiple different directions (see error examples in Fig. 3c). ZS did not create any particles if the traveled distance from one frame to the next became too large (see error example in Fig. 3c). This observation explains why ZS showed a steeper decrease in image quality at higher movement velocities compared with DAIN. Compared with NONE and BIL interpolation both CAFI networks made fewer mistakes (see Fig. 3c and statistical analysis in Supplementary Fig. 4), again highlighting the power of CAFI for cellular dynamic studies.

This observation was confirmed for experimental data obtained from 3D lattice light sheet imaging of a *C. elegans* embryo where we interpolated images in the axial dimension with increasing axial spacing between slices. This can be seen as a change in axial velocity (Supplementary Fig. 3). Here again, we observed that CAFI produced fewer artifacts and better results than the classical NONE and BIL interpolation techniques.

In summary, DAIN and ZS both performed better than NONE and BIL for temporal and axial interpolation on the simulated particle as well as on a real microscopy dataset across different movement velocities, respectively. ZS had slightly better performance for slow to moderately movement speeds; however, ZS made more mistakes for faster object creating blurry artifacts or letting the object disappear. This is where DAIN’s performance is more stable, and it should therefore be used for more challenging and faster-moving datasets.

### iCAFI: 16× faster time resolution

A repeated interpolation on the same dataset with a CAFI model has the potential to produce an even higher temporal image frequency from a given dataset (iCAFI; Fig. 1b). However, this approach may lead to an amplification of the errors introduced by the networks in the first interpolation step. To evaluate the performance of iCAFI, we first generated a simulated dataset that was downsampled, removing every second image in four iterative steps (2×, 4×, 8×, 16× downsampling). We then explored whether the missing frames can be obtained by iteratively applying NONE, BIL, DAIN and ZS predictions (see visual illustration of down- and re-upsampling method in Supplementary Fig. 5).

**Fig. 4:**
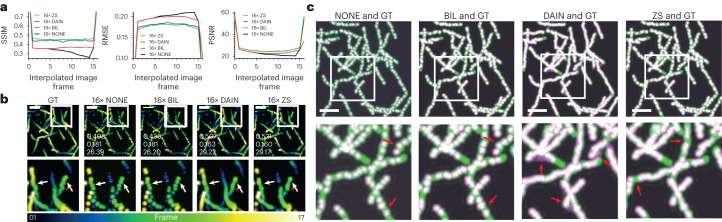
iCAFI allows for 16-fold accurate interpolation of particle motion prediction. **a**, Image quality metrics comparison (SSIM, RMSE and PSNR) of NONE, BIL, DAIN and ZS on multistep image interpolation for every image frame between two ground truth input images. These values represent the averaged metrics over the entire dataset, which comprises 17 interpolated image slices. **b**, Temporal color-coded overlaid projection for visual comparison of 16× interpolated image sequences of ground truth (GT), NONE, BIL, DAIN and ZS; SSIM/RMSE/PSNR of image sequence shown in overlaid images of each interpolation technique. Red stars highlighting example of Brownian motion loss of CAFI compared with GT, and white arrows emphasizing areas of interest for interpolation discrepancies. **c**, Overlaid image-stack projections showing the errors between GT (green) and the interpolated results of NONE, BIL, DAIN and ZS (magenta) with matching overlaid parts (white); white boxes indicate zoomed-in sections and red arrows highlight regions of interest for error comparison (scale bars, 50 pixels; *n* = 17 frame). Source data

The quality metrics diagrams in Fig. 4a show an example for the quality of every frame that was created between two consecutive ground truth image frames (for example, frames 0 and 16). Based on these quality metrics, both CAFI networks showed very similar performance in the multistep interpolation. The first interpolation step (predicting the center frame number 8) showed the biggest image quality drop, since this was the most demanding interpolation step because of the large travel distance of the particles from one image to the next. In the following interpolation steps, which consider smaller travel distances (for example, frames 0 to 8 or 8 to 16 for the second interpolation iteration) the quality for DAIN and ZS started to recover when approaching the original input images (see in particular U-shape PSNR curve in Fig. 4a).

By comparing the overlaid maximum intensity temporal image stack projections of DAIN and ZS with the ground truth, the simulated Brownian motion of the particles got lost in the interpolated image sequence for both networks (see red stars in Fig. 4b and demonstration in Supplementary Video 2). However, the direction of the particles can be accurately captured with CAFI (Fig. 4b, white arrows), which was not possible with the NONE or BIL interpolation. ZS sometimes missed particles in the first interpolation step that could not be recovered in following interpolation steps (Fig. 4c, red arrows), creating gaps in the predicted trajectory. DAIN, however, captured most particles well but created small artifacts that got amplified in the interpolation steps thereafter (Fig. 4c, red arrows). This error amplification explains the slightly worse performance of DAIN compared with ZS for this dataset. Due to the lack of content awareness, the NONE and BIL interpolation were not able to fill the missing spaces between the ground truth input frames of the overlaid temporal projections and therefore performed considerably worse than the two CAFI techniques (Fig. 4c, red arrows).

### CAFI’s SPT analysis

Pixel-based metrics serve as a useful tool for the computer vision research field in evaluating the performance of model predictions. But before deploying any model for use in scientific research, it is essential to confirm that the downstream analyses (for example, segmentation and particle tracking statistics) that use the model predictions are sufficiently accurate. To evaluate the quality improvements of the interpolated image sequences obtained from the CAFI networks in the context of SPT, the different particle velocity datasets were used to perform tracking experiments using the TrackMate^35^ plugin developed in the image analysis platform Fiji^36^. For tracking quality evaluation, the five tracking performance criteria from the ISBI particle tracking challenge were used^33^. The different particle velocity tracks obtained from the predictions of the different techniques (NONE, BIL, DAIN and ZS) were compared with the actual ground truth position of the tracks (for an explanation of how these tracks were generated, see Methods).

**Fig. 5:**
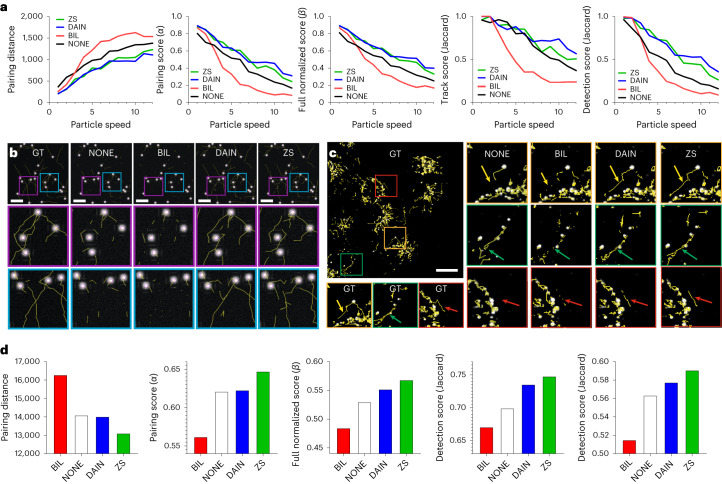
Qualitative assessment of tracking performance of CAFI networks on simulated and experimental data. **a**, Five tracking evaluation metric results of simulated datasets at different particle velocities with NONE, BIL, DAIN and ZS interpolation. **b**, TrackMate tracks comparison of BIL, DAIN and ZS interpolation compared with ground truth tracks at particle velocity V10 (scale bars, 50 pixels; *n* = 17 frames). **c**, Comparison of lysosomal tracking performance on experimental lysosomal dynamics dataset with zoomed-in sections; arrows highlighting region of interest of tracking differences (scale bar, 20 μm, *n* = 20 frames). **d**, Lysosomal tracking performance metrics comparison. Source data

At very low particle velocities, all interpolation methods achieved similar results and ZS performed slightly better than DAIN. The tracking results obtained from CAFI predictions outperformed the tracking results of the BIL interpolation by a large margin, especially for intermediate velocities (see V4 to V8 in Fig. 5a). Surprisingly, NONE interpolation performed better than BIL due to the fact that simple frame duplication does not introduce artifacts to the image sequence, which is happening for BIL in the blending process of two consecutive images. The comparison of the tracks confirmed the superiority of CAFI output over those obtained from NONE and BIL interpolation. Representative tracking results at velocity V7 are shown in Fig. 5b, and a representative particle tracking video is provided in Supplementary Video 3.

We then used the same approach of tracking and image quality evaluation on image sequences of simulated particles with increasing levels of noise (decreasing levels of SNR), by adding random background noise over the signal. We applied frame interpolation using BIL, ZS and DAIN and observed that, for all methods as expected, increasing noise levels lowered the image quality compared with the ground truth of the interpolated image sequences (Extended Data Fig. 6). However, the metrics obtained from DAIN and ZS interpolation results outperformed those for BIL (Supplementary Fig. 6 and Extended Data Fig. 6). In the tracking experiment with the noised data CAFI performed consistently better than BIL over all noise levels but the improved tracking quality dropped with increased noise levels compared with BIL (Extended Data Fig. 6b). The degrading tracking quality was mainly caused by a lack of contrast of the particles created by the interpolated images, which caused a decreased performance of the tracking algorithm to distinguish between a particle and noise signals (Supplementary Fig. 7). However, no more observable artifacts of the interpolated images were observed for the interpolated noised sequences.

### Quantitative analysis for CAFI on lysosomal data

To prove that CAFI can be applied efficiently to real experimental data, we then used CAFI to analyze lysosomal dynamics of live 20× magnified SH-SY5Y cells on a 4D (3D + *t*) dataset. All *z*-slices were projected on one image with maximum intensity projection generating a 2D + *t* dataset. We then removed every other frame to generate a dataset for which ground truth was available. The missing frames from the downsampled dataset were predicted with NONE, BIL and CAFI methods and the stacks were then analyzed with TrackMate. The results were compared with the TrackMate tracks of the original (not downsampled) dataset. On this experimental dataset, ZS performed better in all tracking evaluation metrics than all other interpolation techniques. Due to some generated artifacts generated by the DAIN network, the network was just slightly better than NONE. The five evaluation metrics (Fig. 5d) and the comparison of the tracks show clear improvements of ZS over all other interpolated tracking results (Fig. 5c and Supplementary Video 4), and therefore it is recommended to use the ZS network for this task.

### Nuclei segmentation comparison with NONE, BIL and CAFI

**Extended Data Fig. 1:**
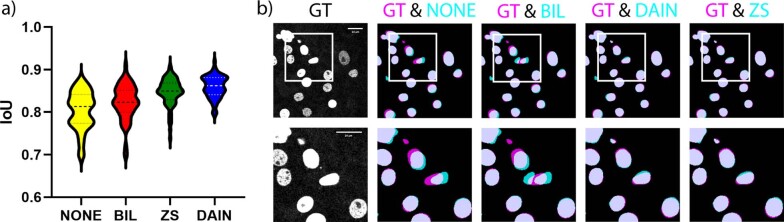
Segmentation analysis with CAFI. Segmentation analysis with CAFI: a) Comparison of the intersection of union (IoU) quality distribution of StarDist segmentation results for 4x iCAFI interpolation (DAIN, ZS) compared to classical interpolation methods (NONE, BIL) on an image sequence of labeled nuclei. The full sample comprised 66 images, and the average IoU for NONE, BIL, DAIN, and ZS was 0.80 ± 0.04, 0.82 ± 0.04, 0.86 ± 0.03, and 0.85 ± 0.03 respectively. b) One representative example for the segmentation improvements of ZS and DAIN over NONE and BIL interpolation (in cyan) compared to the ground truth segmentation (in magenta). Source data

To further test the usefulness of the CAFI tools, a segmentation experiment was carried out. Hoechst 33342-labeled nuclei of N2DH cells were segmented using StarDist^37^ after 4× downsampling and 4× re-upsampling of the image sequence with NONE, BIL, DAIN and ZS. The 4× iCAFI interpolation resulted in fewer segmentation mistakes compared with classical interpolation results with NONE and BIL (Extended Data Fig. 1). This is reflected quantitatively on the basis of the average intersection of union (IoU). The IoU with the segmented ground truth sequence was higher for DAIN and ZS segmentations compared with interpolation results with NONE and BIL (Extended Data Fig. 1a). Here DAIN performed on average slightly better than ZS causing fewer segmentation mistakes. DAIN shows a 3.6% and 5.6% and ZS a 2.3% and 4.2% improvement over BIL and NONE, respectively. Therefore it is recommended to use the DAIN network for this task.

### CAFI performance across microscopy modalities

After investigating the capabilities of DAIN and ZS to improve tracking and segmentation results, we tested their performance on ten more experimental datasets obtained from different microscopy modalities (point-scanning confocal, spinning-disk confocal, lattice light sheet and confocal brightfield microscopy) to demonstrate the versatility of the CAFI approach. The different datasets tested comprise different motion types such as lysosomal movement, cell migration and fibronectin^38^ visualization of different cell lines. Five of these datasets were specifically used to test CAFI’s ability to interpolate 4D (3D + *t*) data where the quality improvements of every slice of DAIN and ZS were directly compared with the results of classical interpolation techniques NONE and BIL.

**Fig. 6:**
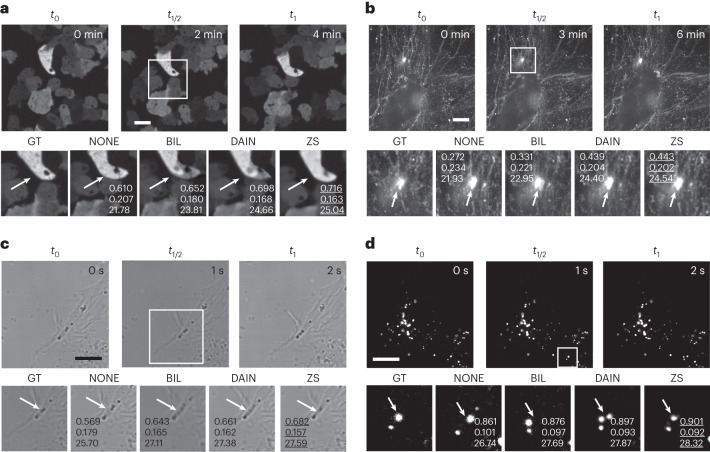
CAFI allows smart interpolation for a wide range of microscopy applications. **a**,**b**, CAFI interpolation comparison of NONE, BIL, DAIN and ZS interpolation shown for *D. discoideum* cells (**a**) and fibronectin-labeled A2780 cells (**b**) (data from Kaukonen et al.)^38^, both recorded on spinning-disk confocal microscopes. **c**, SH-SY5Y cells recorded with a confocal brightfield microscope. **d**, Labeled lysosomes of SH-SY5Y cells recorded with a point-scanning confocal microscope (all scale bars, 10 μm; timestamps in top right corner of GT images); with quality metrics (SSIM/RMSE/PSNR) displayed in zoomed-in images. Arrows highlight regions of interest of visible differences between the interpolation techniques.

**Extended Data Fig. 2:**
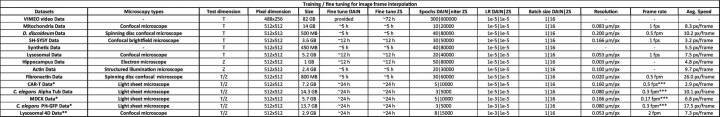
Table of datasets and training parameters. Training data and parameter information for fine-tuning DAIN and ZS networks for the image interpolation task of the different datasets. The other network parameters were kept as default. For datasets labeled with * the fine-tuning of DAIN did not lead to substantial improvements for this dataset and therefore the provided pretrained network was used for the comparisons. For datasets labeled with ** the fine-tuning of DAIN and ZS did not lead to substantial improvements for this dataset and therefore the provided pretrained network were used for the comparisons. The *** indicates that for the 4D datasets the frame rate displayed corresponds to the time dimension.

**Extended Data Fig. 3:**
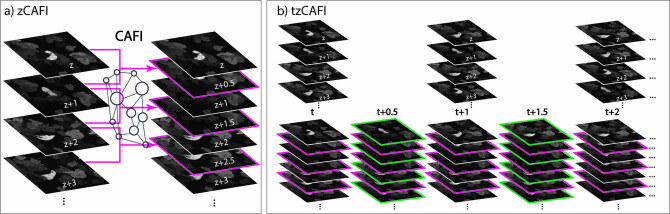
Schematic representation of two more CAFI functionalities (z-CAFI and tz-CAFI). Schematic representation of two more CAFI functionalities. a) zCAFI interpolates images between two consecutive images along the z-axis of a dataset. b) tzCAFI sequentially applies both zCAFI (a) and CAFI (Fig. 1a main text) on 4D (3D+t) dimensional dataset.

**Extended Data Fig. 4:**
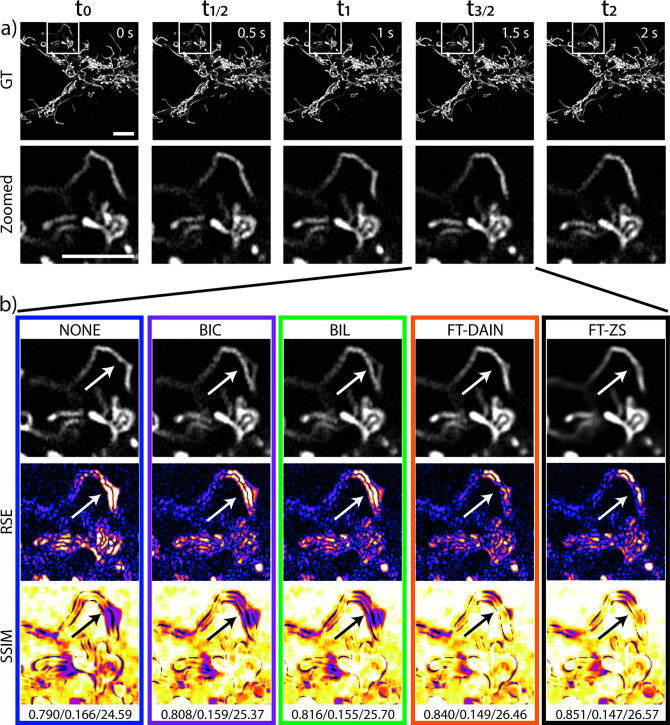
2x Interpolation results on mitochondrial dynamics. 2x frame interpolation of mitochondrial dynamics image sequence of U2OS cells using classical interpolation with NONE, BIL, BIC and CAFI with fine-tuned FT-DAIN and FT-ZS. a) Ground truth (GT) image sequence example (top) with zoomed-in sections (bottom) (scale bar: 5 *μ*m; timestamp in top right corner of GT images). b) Interpolated frame (top), RMSE map (middle) and SSIM map (bottom) with white/black arrows highlighting movement regions; SSIM/RMSE/PSNR displayed below. Data from Fang et al.^30^.

The four microscopy datasets of *Dictyostelium discoideum*, twice SH-SY5Y cells (with brightfield and confocal microscope) and fibronectin images were downsampled where every second frame was removed and kept for ground truth comparison and quality evaluation. For each imaging modality we compared the image quality results of CAFI (DAIN and ZS) with and without fine-tuning, NONE and BIL interpolation. The two CAFI networks were each fine-tuned on images of the same microscopy modality (for more details on the training, see Extended Data Fig. 2 and Methods). The results of the quality evaluation metrics of all datasets are presented in Supplementary Table 2. DAIN and ZS trained only on the VIMEO video dataset already outperformed the classical interpolation techniques for all tested datasets. ZS performed better than DAIN in all datasets and after fine-tuning on the training images of the same imaging modality the prediction quality of both networks further improved. We noted that DAIN sometimes created artifacts by blending the two ground truth image frames together (see arrow in DAIN of Fig. 6a,d). ZS however, sometimes created washed-out and smoothened results where the interpolation details could not be reconstructed with great confidence (see arrow in ZS of Fig. 6c). More detailed interpolation comparisons are shown in Extended Data Figs. 7–10, and video demonstrations of 2× and examples of 4× (iCAFI) frame interpolation are presented in Supplementary Videos 5–8.

Given that CAFI is ultimately a smart interpolation tool, we also demonstrate that CAFI can perform smart interpolation of *z*-stacks on 3D datasets (see Supplementary Figs. 8 and 9, Supplementary Videos 9 and 11 and Supplementary Table 3 for quality comparisons). Additionally, CAFI’s *t*- and *z*-interpolation ability was further investigated on five 4D datasets (*tzxy*). A frame-wise interpolation comparison between image quality obtained from CAFI and NONE and BIL interpolations showed a consistent improvement of the neural network approach over the classical techniques. The amount of improvement, however, was dependent on the individual dataset at hand and can therefore vary in its degree. It was more pronounced for image frames where more movement was occurring compared with very static image slices. However when too much change occurs in the sequence, the performance increase of the networks is also less pronounced. In most cases, the quality of the neural network interpolated images improved for the SSIM metric in comparison with BIL and most obviously compared with NONE interpolation. Also, the RMSE metric showed strong improvements for several datasets. The datasets were composed of four lattice light sheet microscopy datasets imaging CAR-T, MDCK cells and two *C. elegans* embryo recordings and one confocal microscopy dataset of lysosomes labeled SH-SY5S cells. The visualization workflow is shown and explained in Supplementary Fig. 10, and the results are visualized in Supplementary Figs. 11–25. In the visualizations every second slice in the temporal dimension was removed for better visibility of the quality differences. Furthermore, comparison videos of these datasets are provided in Supplementary Videos 12–16.

Furthermore, a particularity of ZS is that it is also capable of performing lateral upsampling (Supplementary Fig. 26). We tested this capability and also demonstrated its great performance for this task. For more details see Supplementary Note 2, and demonstrated examples are shown in Supplementary Figs. 27–33 with training conditions and quality comparisons presented in Supplementary Tables 4–6.

## Discussion

In this work, we present two implementations of state-of-the-art CAFI neural networks (ZS^16,17^ and DAIN^15^) that can effectively increase the frame rate of time-lapse microscopy data by predicting intermediate frames between two consecutive images. Both neural networks showed great performance on mitochondria dynamics (Fig. 2) and particle tracking tasks from both simulated and experimental datasets (Figs. 3–5). We compared their performance with classical interpolation (NONE and BIL) and showed that content awareness offered better-quality images based on a range of image metrics. Furthermore, iCAFI allowed for iterative interpolation of the same dataset allowing for 16× interpolation. The first interpolation step of iCAFI was the most demanding and could lead to artifacts that might be amplified in the following iterations. As expected, we noticed that random movement patterns such as Brownian motion could not be reconstructed using CAFI, and these tools should therefore not be used if this is the subject of interest. However, the general directed motion of objects was successfully recovered in a smoothened fashion.

The CAFI networks were also capable of predicting a range of dynamic movement patterns as demonstrated on 12 different datasets from four different microscopy modalities (point-scanning confocal, spinning-disk confocal, lattice light sheet and confocal brightfield microscopy). Within these datasets, the average speed of the fast-moving objects ranged from 3 to 26 pixels per frame, demonstrating the complexity of the task (see last column of Extended Data Fig. 2). For each dataset both CAFI networks outperformed classical interpolation techniques such as NONE and BIL interpolation even without fine-tuning of the networks on the images of the same microscopy modality. In most cases the performance increased after fine-tuning and both networks were capable of learning and predicting the more complex cellular movement patterns. Furthermore, their strengths and weaknesses in the context of image artifact generation were assessed. For fast-moving objects both CAFI networks started to make mistakes and created notable image artifacts; however, for slow and moderate object movement speeds both networks performed very well in their interpolation tasks where DAIN showed more stable results for higher movement speeds and ZS demonstrated greater precision for slower dynamics. ZS created blurry artifacts or missed fast-moving objects, and DAIN created artifacts by creating movement in different directions. ZS showed the stronger improvements for tracking performance, and DAIN was better suited for improving segmentation of nuclei. Overall, the network improvement was dependent on the quality and amount of training data available. For more details, see Extended Data Fig. 2.

Finally, DAIN and ZS also outperformed NONE and BIL in the axial interpolation, and the lateral upsampling functionalities of ZS was found to achieve good performance, too.

These universal content-aware image interpolation solutions show great potential for any microscopy modalities that would benefit from a reduced laser exposure on the sample or a higher frame rate to investigate fast cellular processes. Despite their potential, CAFI networks may still produce image artifacts when imaging fast objects. So, importantly, quality control of model output on appropriate validation data should be performed in the context of downstream analyses (for example, segmentation and particle tracking) before implementing these tools for real-world investigations, as has been suggested^39^. In the future, the simultaneous analysis of multiple channels could provide additional inputs for identifying the dynamics. We also expect that neural network architectures that have memories for more than the two adjacent frames considered here to improve the performance of CAFI approaches, but at the cost of higher computational costs and complexity. The increasing availability of public datasets, especially of dynamic datasets, will also improve the capabilities to build more general models for microscopy and high-performance pretrained models with efficient fine-tuning.

Content awareness therefore constitutes a powerful approach for microscopy as it facilitates the recovery of high-quality datasets using knowledge embedding acquired at the training stage. As was previously demonstrated for spatial resolution improvement^1^, here we show that temporal resolution can also be recovered accurately using CAFI networks such as DAIN and ZS. We expect that this content awareness may exploit additional context from large multidimensional imaging datasets. However, it is important to remember that using content awareness for image restoration heavily relies on the assumptions that the training dataset fully encompasses all the types of dynamics that will be observed in the analyzed dataset. Deviation from this will almost inevitably lead to errors. So, although CAFI provides a powerful tool for live-cell microscopy, it should be considered only when the acquisition of such datasets is not physically possible and only when the outputs have been validated on downstream workflows using real-world ground truth data. We anticipate that tracking large and nonlinear dynamics could pose substantial challenges for CAFI and that beyond the datasets and training regimes presented here the performance of CAFI is unknown. A comprehensive analysis of this issue falls beyond the scope of the present study; however, we speculate that rapid and abrupt events, such as fusion or fission of mitochondria or endoplasmic reticulum, might prove particularly demanding for our method. One potential avenue for overcoming these difficulties is the use of high-quality ground truth datasets, which can leverage the inherent adaptability of DL techniques to improve performance. In the right settings CAFI is thought to be a valuable tool that could be used to improve several different challenges in microscopy such as faster multicolor imaging, long-term imaging or high-throughput imaging. A nonexhaustive list of further possible applications for this tool is presented in Supplementary Table 9.

In summary, here we demonstrate the potentials and versatility of CAFI to improve the frame rate of many microscopy imaging modalities in need for a higher frame frequency. We also provide the tools and pretrained models used in this paper to the wider scientific community through the ZeroCostDL4Mic platform.

## Methods

### Simulated dataset

The simulated dataset was created using the ICY plugin from the 2014 ISBI particle tracking challenge^33,34^. The datasets for temporal interpolation consisted of ‘switching uniform’ white particles moving in front of a black background. The tool allowed to precisely control the image and particle parameters. The image parameters included options for image dimensions, temporal frame density and SNR. The parameter related to particle appearance and behavior included particle size, velocity and variables for selecting the contributions of Brownian (sigma) and directed motion fraction. The sigma parameter describes the particle displacement distance of Brownian motion, and the directed motion fraction parameters describe the probability for Brownian motion and directed motion to occur. The parameter of ‘*xy* pixel size’ was chosen to generate particles with diameters of roughly 15 pixels, which was a good size estimate for real-life lysosomes recorded with 63× magnification. The movement-related parameters were empirically selected to simulate the behavior of lysosomal dynamics most accurately. For generating the training data, we selected a particle velocity range between 2 and 10, which encompasses a broad spectrum of movement speeds to ensure diverse training data. All parameter values for the particle simulation datasets are provided in Supplementary Table 1.

For the different velocity experiments, one densely framed image sequence (225 frames) was generated. To generate the different ground truth velocities an increasing number of images for each velocity dataset was removed between each two consecutive time points (1 to 12 removed images labeled as velocities V2 to V13). Therefore, each dataset had a gradually increased particle travel distance from one frame to the next and therefore also an increased overall particle velocity. These ground truth velocity datasets where then downsampled by removing every second image before re-interpolating them with the provided different interpolation techniques (see visual illustration of this preparation methodology in Supplementary Fig. 1). Furthermore, the ground truth image sequences were limited to 17 frames to make them easily comparable.

For the iCAFI multistep temporal interpolation experiment, the same densely framed simulated dataset (225 frames) was downsampled, removing every second image in four iterative steps (2×, 4×, 8×, 16× downsampling), while keeping the removed images as ground truth for comparison for the following iterative re-interpolation steps (see Supplementary Fig. 5 for visual illustration of the down- and re-upsampling process). As training data for fine-tuning of the CAFI networks, 10 simulated datasets with 225 images per sequence were created with the same parameters as the test data, including the full equivalent range of velocities to be predicted (equivalent to velocities V2 to V13).

### Particle tracking

Particle tracking was performed using the Fiji plugin TrackMate^35^. For detecting the simulated dataset particles, the LoG detector with an estimated blob diameter of 10 and a threshold of 5 was used. The linking step was performed with the ‘Simple LAP tracker’ with a linking distance of 25 μm, gap-closing of 25 μm and maximum frame gap of 2. For the full set of parameters for the different particle tracking scenarios as well as for the real-life lysosomal tracking experiment, see Supplementary Tables 7 and 8.

### Particle velocity ground truth tracks generation

The ground truth tracks for the different particle velocities were generated in the following steps. First the big, simulated dataset (225 frames) was generated with the ICY data generator. This plugin provided a XML file with all the ground truth time points and precise particle point locations. This XML file was first converted into the ISBI XML format in the TrackMate interface, and the relevant time points of this file were downsampled in the same way as the actual image sequences, where an increasing number of frames was removed between each time point (see visual illustration of method in Supplementary Fig. 1). A developed Python script selected just the time points and particle coordinates relevant for the specific movement velocity and generated a new XML file containing just the locations of the particles for the selected frames in that particle velocity option. These ground truth tracks were then compared with the TrackMate detected tracks of the BIL and the CAFI interpolated image sequences, and the five performance criteria from the ISBI particle tracking challenge^33^ were evaluated with the associated ICY ISBI Challenge Tracking Batch Scoring plugin for comparing the different tracking files.

The ground truth tracks for the real-life lysosomal data were evaluated as the TrackMate generated tracks of the full image sequence before downsampling of the dataset. The evaluated tracks from TrackMate were exported in the ISBI challenge format. Then the TrackMate tracks of the downsampled and re-interpolated image sequences were compared with the ground truth tracks using the ISBI Challenge Tracking Batch Scoring plugin from ICY^33^.

### Noise experiment

A simulated particle dataset with a 225-image sequence was created with the ICY track generator^40^ with the same settings as shown in Supplementary Table 1. The plugin-created data provided the ground truth positions of the particles and the associated tracks. The image sequence and tracks were downsampled to create a ground truth dataset with particle velocity V5 by selecting every fifth image of the sequence. This was achieved by using a custom Python script for downsampling the tracks and the Google Colab implementation for downsampling the image sequence. Then different levels of noise were added to the image sequences (Gaussian noise with standard deviation 0–80) using Fiji^36^, and each resulting corrupted sequence was then further downsampled in the temporal dimension by a factor of 2, which was used for the interpolation inference afterward. These image sequences with the different noise levels were then re-interpolated with BIL and the provided pretrained networks DAIN and ZS, and the image quality metrics (SSIM, RMSE and PSNR) were evaluated by comparing them with the noisy but not downsampled ground truth sequences. Furthermore, TrackMate^35^ was used to extract the tracks of the noisy images (estimated blob diameter 15, threshold 1.5, linking distance 15, gap-closing distance 15, gap-closing max frame gap 1). All the tracks were compared with the ground truth tracks using the ICY ISBI batch scoring plugin^33,34^, and the results are shown in Supplementary Fig. 6.

### *z*-Velocity interpolation

A 3D dataset (*zxy*) of a *C. elegans* embryo recorded with light-sheet microscopy with 300 slices in the *z*-dimension was increasingly downsampled removing 1–15 frames (*z*-velocity) between two consecutive frames creating different ground truth datasets. These datasets were again temporally downsampled by a factor of 2, then re-interpolated with the DAIN, ZS network and classical NONE and BIL interpolation. Then the averaged quality of the interpolated images against the ground truth was plotted and is shown in Supplementary Fig. 3.

### Segmentation experiment

An image sequence of Hoechst 33342-labeled nuclei of N2DH cells^37^ over time (*t* = 89 frames) was first downsampled in the temporal dimension to 23 frames and then re-interpolated to its original frame density (4× iCAFI) with NONE, BIL, DAIN and ZS. Then all sequences were used for segmentation with the StarDist2D plugin^37^ in Fiji^36^ (model: versatile (fluorescent nuclei), normalized image; percentile low: 1; percentile high: 99.8; probability: 0.5; overlap threshold: 0.45). The mean IoU with the segmented ground truth sequence was calculated using the StarDist Google Colab notebook from the ZeroCostDL4Mic platform^29^, which implemented the following formula, where *A* is set 1 and *B* is set 2:

$$\begin{array}{l}{\mathrm{IoU}}(A,B)=\frac{| A\cap B| }{| A\cup B| }.\\ \end{array}$$

### 4D interpolations

Five different 4D (*tzxy*) datasets were downsampled by a factor of 2 in *t*- and *z*-dimension and re-interpolated with the neural networks DAIN and ZS and with the classical NONE and BIL techniques. Then the image quality metrics (SSIM, RMSE and PSNR) of each frame of the neural network interpolated image sequences was calculated with respect to the same frame of the classical interpolation techniques. For better visualization, every second frame in *z*-dimension was removed in the 2D visualization for Supplementary Figs. 11–22 and every second *t*-dimension frame for Supplementary Figs. 23–25 (lysosomes) (see visualization workflow in Supplementary Fig. 10, and the results are visualized in Supplementary Figs. 11–25).

### Network training

The ZS interpolation with the options of 1× (no lateral upsampling) and 2× lateral upsampling was initially trained from scratch on the Vimeo90K-septuplet^24^ (82 GB) dataset. First the full dataset was split into smaller 8–12 GB subdatasets, which was necessary because of quota limitations of the Google Colab environment. Then the training was carried out for a total of 300 epochs for DAIN and 600,000 *n*_iter_ for ZS. Before using the two CAFI models for the different interpolation tasks both networks were fine-tuned with 0.5 to 14 GB of images of the same imaging modality, which was depended on the amount of available training data. To increase the amount of available data, image augmentation techniques (rotation and mirroring) were applied to the available training data. Furthermore, for images with image dimensions other than 512 × 512, they were cropped or zero-padded to this dimension. The learning rate for training of both networks was reduced to 1 × 10^−5^, while the other parameters were used as provided from the original papers^15,16^. The average movement speed of the objects within each dataset was calculated by manually tracking three fast-moving objects using TrackMate and averaging the measured velocity. For more details on the training datasets, resolution and additional parameters of each dataset, see Extended Data Fig. 2.

In most cases fine-tuning of the networks leads to improved performance; however, for the 4D interpolation experiment the training of ZS failed on one and for DAIN on four of the datasets (as indicated in Extended Data Fig. 2), leading to reduced performance compared with the provided pretrained networks. For those datasets, the results from the pretrained networks were used for the comparison studies and already showed good improvements over the classical interpolation techniques.

For the training of the lateral upsampling functionality, ZS and SRFBN-S were fine-tuned on 3.6–12.1 GB training data with a learning rate of 1 × 10^−5^ and 1 × 10^−4^, respectively. For fine-tuning of ZS for 4× lateral upsampling of the electron microscopy dataset to compare it with the PSSR network, gaussian noise was added to the provided training data as it was done in the original paper^30^ (for more details on the datasets and the epoch/*n*_iter_ sizes, see Supplementary Table 4).

To increase the amount of available training data of the *D. discoideum* dataset, the recorded images with a resolution of 1,200 × 1,200 pixels were augmented by zooming to each corner part of the image with the image size of 512 × 512 pixels and by resizing the full target image down to 512 × 512 pixels.

The data preparation for small datasets was performed in the provided Google Colab notebooks. For datasets bigger than 2 GB the training data preparation was performed offline with the Python data preparation scripts provided in the GitHub repository. The created folders were then uploaded on Google Drive/Google Cloud Storage for training in the Google Colab environment.

### DAIN architecture

The DAIN, developed by Wenbo et al. in 2019 (ref. ^15^), uses depth information to detect occlusions and perform frame interpolation. It employs hierarchical features and an adaptive wrapping layer, integrating input frames, depth maps and contextual features. Pretrained networks such as an hourglass network (a special type of convolutional encoder-decoder network) pretrained on the MegaDepth dataset^41^, PWC-Net^42^ and ResNet^43^ contribute to depth mapping, flow estimation and contextual information, respectively. To ensure that the network predicts residuals between the ground truth frame and the blended frame, the two warped frames are linearly blended. A U-Net is used for kernel estimation, and all data streams are combined in an adaptive wrapping layer. The network is pretrained on the Vimeo90K dataset^24^, a large collection of high-quality video clips. For further details, consult the original paper^15^.

### ZS architecture

The ZS network, inspired by Xiang et al.’s 2021 work^16^, enables both VFI and video super-resolution in a single step, enhancing image resolution by up to 4×. It comprises four key components: a feature extractor, a temporal interpolation module, a deformable ConvLSTM and a high-resolution frame reconstructor. The network utilizes deformable feature interpolation for temporal context and a ConvLSTM for time-aligned aggregation. This facilitates effective motion handling and global context leverage. For an in-depth understanding, refer to the original paper^16^.

### Lysosome cell imaging

SH-SY5Y cells (CRL-2266) were cultured in Dulbecco’s modified Eagle medium (DMEM, Invitrogen) supplemented with 10% fetal bovine serum (FBS, Invitrogen), glutamine (2 mM) and penicillin/streptomycin (50 μg ml^−1^, Invitrogen). All cells were grown in a 5% CO_2_ incubator at 37 °C. The cells were plated and grown on eight-well chamber slides (LabTek II Chamber Coverglass) in 250 μl of culture medium at a plating density of 25,000 cells per well and allowed to grow for 24 h. Next the medium was changed to medium containing Lipofectamine 2000 (2 μl ml^−1^) and the lysosomal copper(I) probe FLCS1 (60 nM)^44^. The cells were incubated with this dye for 24 h. Before imaging, the cell medium was changed back to DMEM with 10% FBS (250 μl per well). The image acquisition was performed on a confocal microscope (Leica SP5) with 63× magnification 1.4 numerical aperture (NA) oil objective. Three-dimensional image (*xyzt*) time series were recorded for 20 min collecting 40 time points of 30 *z*-stack images at a resolution of 512 pixels (0.481 μm per pixel) and temporal time sequences (*xyt*) were recorded with the same settings at an imaging speed of 1 s per frame.

### *D. discoideum* cell imaging

*D. discoideum* cells were genetically engineered to transiently express enhanced GFP (eGFP) and tdTomato from an extrachromosomal plasmid and grown adherently in HL5 medium. Before imaging, cells were washed in KK2 medium and transferred onto KK2-agar pads at a density of approximately 5 × 10^5^ cells cm^−2^. Once cells had adhered to the pads, the pads were inverted onto ibidi U-dishes and overlaid with silicone oil to prevent dehydration. Samples were imaged on an inverted spinning-disk confocal microscope (3i), using a 63× 1.4 NA oil objective and equipped with a prime95B CMOS camera (photometrics). Three-dimensional image (*xyzt*) time series were recorded with 2-min frame intervals.

### Lattice light sheet microscopy

We used a custom-built lattice light sheet microscope (LLSM)^45^ to image biological samples. The samples were illuminated by a 488 nm diode laser (0.5 W coherent), 561 nm diode laser (0.5 W, coherent) or 647 nm diode laser (1.0 W coherent) using an excitation objective (special optics, 0.65 NA with a working distance of 3.74 mm) at 2–10% acousto-optic tunable filter transmittance and laser power of 100 mW for any given laser line. Order transfer functions were obtained empirically by acquiring point-spread functions using 200-nm TetraSpeck beads adhered freshly to 5-mm glass coverslips (Invitrogen T7280) for each wavelength and for each day of experiments. To achieve lattice light sheet illumination, a square lattice was displayed on a spatial light modulator. This lattice was generated by an interference pattern of 59 Bessel beams separated by 1.67 μm and cropped to 0.22 with a 0.325 inner NA and 0.40 outer NA. The lattice light sheet was dithered 10–25 μm to obtain homogeneous illumination with 5–7% flyback time. Fluorescent signal was collected by a Nikon detection objective (CFI Apo LWD 25XW, 1.1 NA, 2-mm working distance), coupled with a 500 mm focal length tube lens (Thorlabs), a Semrock filter (BL02561R-25) and sCMOS cameras (Hamamatsu Orca Flash 4.0 v2) with a 103 nm per pixel magnification. *z*-Stacks were acquired by moving the lattice light sheet and the detection objective synchronously using a galvo mirror coupled at the back focal plane of the illumination objective and a piezomotor, respectively. The slices of the stacks were taken with an interval of 100 nm through ranges of 30–50 μm at 100 ms camera exposure with 1–5-s intervals between *z*-stacks. Raw data were flash corrected^46^ and deconvolved using an iterative Richardson–Lucy algorithm^45^ run on two graphics processing units (NVIDIA, GeForce GTX TITAN 4 GB random-access memory). Flash calibration, flash correction, channel registration, order transfer function calculation and image deconvolution were done using the LLSpy open software^47^. Visualization of the images and volume inspection were done using Spimagine^48^, ClearVolume^49^ and Napari software^50^. *C. elegans* embryos were extracted from dissected adult worms in M9 Buffer and then transferred to freshly prepared imaging medium 10 mM Tris–Cl pH 8.5 (EB buffer). Acquisition filter used was the Semrock 446-523-600-677. Microscopy acquisition was executed at room temperature (approximately 24 °C). To avoid any movement of the embryos, embryos were glued to the 5-mm coverslip using CellTak (Corning). CellTak was curated with 70% ethanol and rinsed with distilled water no longer than 3 h before the experiment. A volume was acquired by sectioning of 301 slices separated by 100 nm. Laser power was an AOTF power of 10% of 50 mW 488 nm diode laser power. Exposure time was 50 ms per slide. Three-hundred consecutive volumes were acquired in this experiment. GFP–α-tubulin and GFP-PH (tagged PH-domain of phospholipase C δ 1) *C. elegans* embryos were kindly provided by the Hyman Lab from Max Planck Institute of Molecular Cell Biology and Genetics (MPI-CBG). MDCK II (Sigma cat. no. 00062107) cells were cultured at 37 °C and 5% CO_2_ in DMEM (ThermoFisher cat. no. 11885076) supplemented with 10% FBS (Corning) and 1% penicillin–streptomycin (ThermoFisher). Live-cell lattice light sheet microscopy was performed in L15 medium supplemented with 1% FBS and insulin–transferrin–selenium (Invitrogen). The samples were incubated for 12–36 h at 37 °C in 25-mm tissue culture plates. At least 12–16 h before experiments, samples were transferred to the LLSM imaging medium (L15 medium supplemented with 1% FBS and insulin–transferrin–selenium, Invitrogen). MDCK cells constitutively expressing Gap4–mCherry were generated to visualize the membrane. Briefly, cells were transfected with a plasmid containing the PiggyBac transposon system and the Gap4–mCherry sequence (DNA2.0), and cells were selected for plasmid integration with geneticin (G418, ThermoFisher). MDCK cells were plated on the 5-mm glass disk and let grow for at least 24 h. A volume was acquired by sectioning of 301 slices separated by 100 nm. Laser power was an AOTF power of 5% of 100 mW 561 nm diode laser power. Exposure time was 25 ms per slide. One-hundred consecutive volumes were acquired in this experiment. The volume was generated by moving the sample piezo through the static lattice light sheet.

### Production of retroviral supernatant and CAR-T cells

Retroviral supernatant was produced as previously described^51^. Using the RosetteSep T cell Enrichment Cocktail (STEMCELL Technologies), T cells were isolated from healthy donor buffy coats that were obtained through the Stanford Blood Center under an institutional review board-exempt protocol. CAR-T cells were generated and cultured as previously described^52^. Briefly, cryopreserved T cells were thawed on day 0 and cultured with Human T-Activator *α*CD3/CD28 Dynabeads (Gibco) at 3:1 bead:cell ratio in AIM-V medium (Gibco) supplemented with 5% FBS, 10 mM HEPES, 2 mM GlutaMAX, 100 U ml^−1^ penicillin, 100 μg ml^−1^ streptomycin and 100 U ml^−1^ recombinant human IL-2 (Peprotech). Retroviral transductions were performed on T cells on days 3 and 4 post activation on retronectin (Takara) coated non-tissue culture-treated plates. On day 5 post-activation, *α*CD3/CD28 beads were magnetically removed and CAR-T cells were maintained in culture with AIM-V medium changes every 2–3 days at a density of 0.3 × 10^6^ cells ml^−1^.

### Reporting summary

Further information on research design is available in the Nature Portfolio Reporting Summary linked to this article.

## Online content

Any methods, additional references, Nature Portfolio reporting summaries, source data, extended data, supplementary information, acknowledgements, peer review information; details of author contributions and competing interests; and statements of data and code availability are available at 10.1038/s41592-023-02138-w.

### Supplementary information

Supplementary Information All supplementary information as a single PDF.

Reporting Summary

Supplementary Video 1 Demonstration of CAFI networks for temporal interpolation on point-scanning confocal microscopy dataset of fluorescently labeled mitochondria branches (data from Fang et al.^30^). Scale bars correspond to 5 μm and 2.5 μm for the full and zoomed-in video, respectively.

Supplementary Video 2 Demonstration of CAFI networks for temporal interpolation of a simulated particles dataset generated with the ISBI particle tracking challenge plugin from Icy^34^. Particle diameter is 15 pixels, and the selected movement velocities range from V2 to V8.

Supplementary Video 3 Demonstration of TrackMate^35^ tracking improvements of simulated particles generated with the ISBI particle tracking challenge plugin from Icy^24^ after temporal interpolation with CAFI. Particle diameter is 15 pixels, and the movement velocities selected range from V2 to V8.

Supplementary Video 4 Demonstration example of lysosomal tracking improvements after temporal interpolation with CAFI using TrackMate^35^. Lysosomes were labeled with FLCS1 (ref. ^44^), and the image sequence was collected as 3D + *t* dataset on a Leica SP5 with a 63× magnification 1.4 NA oil objective. *z*-Stacks were projected with maximum intensity generating a 2D + *t* dataset. The tracking results from the full image sequence was taken as ground truth tracks for quality comparison. Scale bars correspond to 20 μm and 6 μm for the full and zoomed-in video, respectively.

Supplementary Video 5 Demonstration of CAFI networks for temporal interpolation on inverted spinning-disk confocal microscopy dataset of GFP-labeled *D. discoideum* cells. Images were recorded using a 63× 1.4 NA oil objective with 2-min frame intervals. Scale bars correspond to 10 μm and 4 μm for the full and zoomed-in video, respectively.

Supplementary Video 6 Demonstration of CAFI networks for temporal interpolation on a spinning-disk confocal microscopy dataset of fluorescently labeled fibronectin of A2780 cells (data from Kaukonen et al.; scale bars correspond to 10 μm and 3 μm for the full and zoomed-in video, respectively^38^).

Supplementary Video 7 Demonstration of CAFI networks for temporal interpolation on confocal brightfield dataset of SH-SY5Y cells. Images were recorded on a Leica SP5 with a 63× magnification 1.4 NA oil objective with 1-s frame intervals. The image sequence was downsampled removing every second image in two iterative steps and re-interpolated in temporal dimension with 2× CAFI and 4× iCAFI. Scale bars correspond to 10 μm and 4 μm for the full and zoomed-in video, respectively.

Supplementary Video 8 Demonstration of CAFI networks for temporal interpolation on a point-scanning confocal microscopy dataset of fluorescently labeled lysosomes of SH-SY5Y cells. Images were recorded on a Leica SP5 with a 63× magnification 1.4 NA oil objective with 1-s frame intervals. The image sequence was downsampled removing every second image in two iterative steps and re-interpolated in temporal dimension with 2× CAFI and 4× iCAFI. Scale bars correspond to 5 μm and 2 μm for the full and zoomed-in video, respectively.

Supplementary Video 9 Demonstration of zCAFI networks for axial interpolation on an electron microscopy dataset of rat hippocampus (data from Fang et al.^30^). The image sequence was downsampled removing every second image in two iterative steps and re-interpolated in axial dimension with 2× CAFI and 4× iCAFI. Scale bars correspond to 0.2 μm and 0.08 μm for the full and zoomed-in video, respectively.

Supplementary Video 10 Demonstration of zCAFI networks for axial interpolation on a structured illumination microscopy (SIM) dataset of fluorescently labeled actin of DCIS.COM cells (data from Kaukonen et al.^38^). Scale bars correspond to 10 μm and 2 μm for the full and zoomed-in video, respectively.

Supplementary Video 11 Demonstration of tzCAFI on fluorescently labeled fibronectin of A2780 cells on a 4D spinning-disk confocal microscopy dataset (data from Kaukonen et al.^38^). The networks were trained for the interpolation task on data in the temporal dimension and the same fine-tuned network was used for both interpolation dimensions (axial and temporal). Scale bars correspond to 5 μm.

Supplementary Video 12 Demonstration of tzCAFI on *C. elegans* embryo labeled with GFP targeting α-tubulin recorded on a 4D LLSM. Due to data limitation the networks were just fine-tuned on augmented (90°, 180°, 270° rotated and mirrored) images of the tested dataset. This resulted in improvements for ZS but not for DAIN where the pretrained network showed already improved performance and was finally used for this comparison.

Supplementary Video 13 Demonstration of tzCAFI on CAR-T cells recorded on a 4D LLSM. Scale bars correspond to 8 μm. Due to data limitation, the networks were just fine-tuned on augmented (90°, 180°, 270° rotated and mirrored) images of the tested dataset. This resulted in improvements for ZS but not for DAIN where the pretrained network showed already improved performance and was finally used for this comparison.

Supplementary Video 14 Demonstration of tzCAFI on MDCK cells recorded on a 4D LLSM. Scale bars correspond to 8 μm. Due to data limitation, the networks were just fine-tuned on augmented (90°, 180°, 270° rotated and mirrored) images of the tested dataset. This resulted in improvements for ZS but not for DAIN where the pretrained network showed already improved performance and was finally used for this comparison.

Supplementary Video 15 Demonstration of tzCAFI on *C. elegans* embryo labeled with GFP targeting GFP–PH recorded on a 4D LLSM. Scale bars correspond to 8 μm. Due to data limitation, the networks were just fine-tuned on augmented (90°, 180°, 270° rotated and mirrored) images of the tested dataset. This resulted in improvements for ZS but not for DAIN where the pretrained network showed already improved performance and was finally used for this comparison.

Supplementary Video 16 Demonstration of tzCAFI on fluorescently labeled lysosomes in SH-SY5Y cells recorded on recorded on a confocal microscop. Scale bars correspond to 8 μm. Due to data limitation, the networks were just fine-tuned on augmented (90°, 180°, 270° rotated and mirrored) images of the tested dataset. For this dataset fine-tuning did not improve the performance of the networks, and therefore the already well-performing pretrained networks were used for this comparison.

Supplementary Data 1 Velocity interpolation in z-dimension results comparing SSIM, RMSE, PSNR of the different interpolation techniques.

Supplementary Data 2 Statistical analysis for particle velocity experiment.

Supplementary Data 3 Image quality assessment of noised simulated particle dataset.

Supplementary Data 4 Quality evaluation metric comparison of ZS compared to PSSR for 4x lateral upsampling on EM dataset.

### Source data

Source Data Fig. 2 SSIM, RMSE and PSNR of mitochondria frame interpolation comparison for the different interpolation techniques.

Source Data Fig. 3 Quantitative assessment of CAFI’s performance on simulated data with increasing particle speed.

Source Data Fig. 4 iCAFI SSIM, RMSE and PSNR results for 16× interpolation.

Source Data Fig. 5 a, Quantitative assessment of tracking performance of all interpolation techniques on simulated data. b, Quantitative assessment of tracking performance of all interpolation techniques on real lysosomal dynamics data.

Source Data Extended Data Fig. 1 Segmentation analysis data for comparing the IoU for the different interpolation techniques.

**Extended Data Fig. 5:**
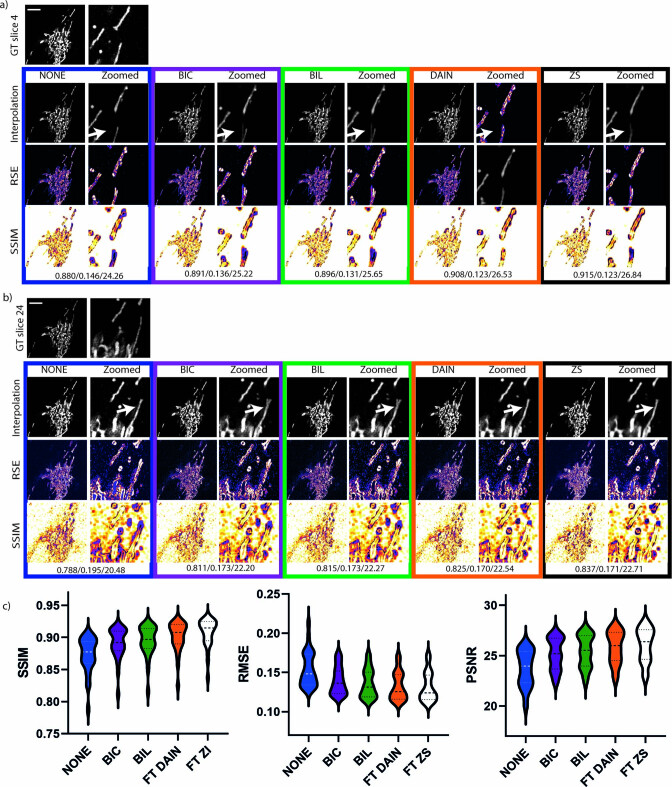
Second example of mitochondrial dynamics 2x interpolation. Interpolation analysis of second mitochondria dynamics image sequence of U2OS cells using classical interpolation with NONE, BIL, BIC and CAFI with fine-tuned FT-DAIN and FT-ZS. Interpolated frames, RMSE and SSIM images are shown of the different interpolation techniques with zoomed-in sections next to it (scale bar: 5 *μ*m; n=29 interpolated image frames). a) Showing frame 4 and b) frame 24 of the same sequence. c) Showing violin plots of the SSIM, RMSE and PSNR error of the different simulation techniques. Data from Fang et al.^30^. Source data

**Extended Data Fig. 6:**
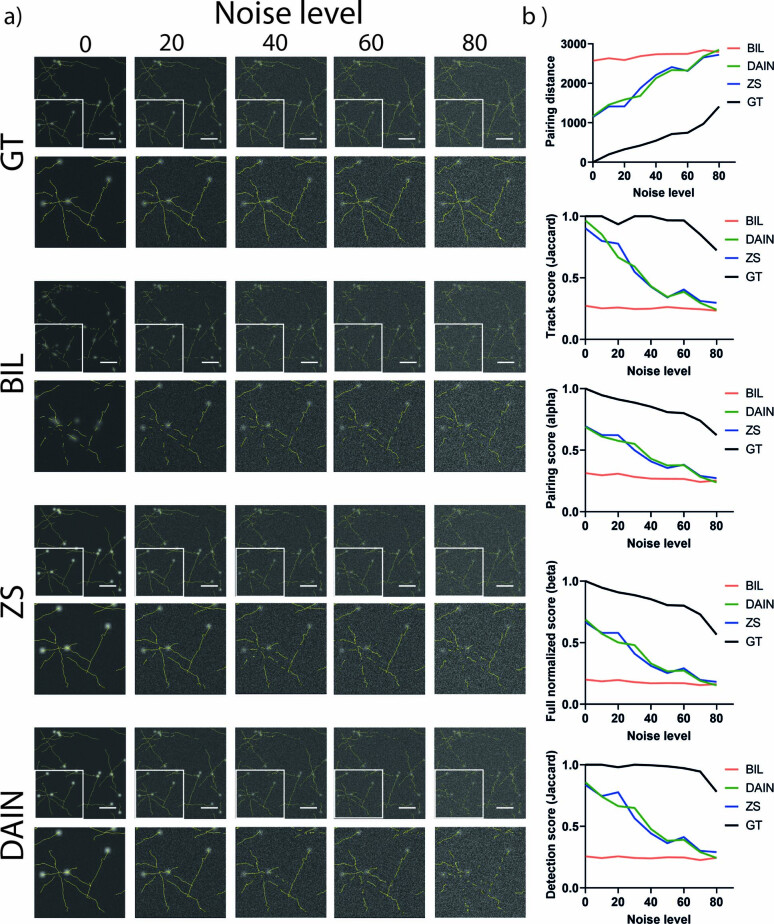
Tracking experiment on noised simulated data. Tracking of noised simulated particle dataset. a) Simulated particle dataset generated with ICY track generator^40^ with different levels of noise added with Fiji^36^ were tracked with TrackMate^35^ after interpolating with BIL, DAIN and ZS. GT described the comparison of the tracks of noised ground truth sequence to the real generated tracks. b) Five tracking evaluation metric results of simulated particles at different noise levels with BIL, DAIN and ZS interpolation (n=45 images averaged).

**Extended Data Fig. 7:**
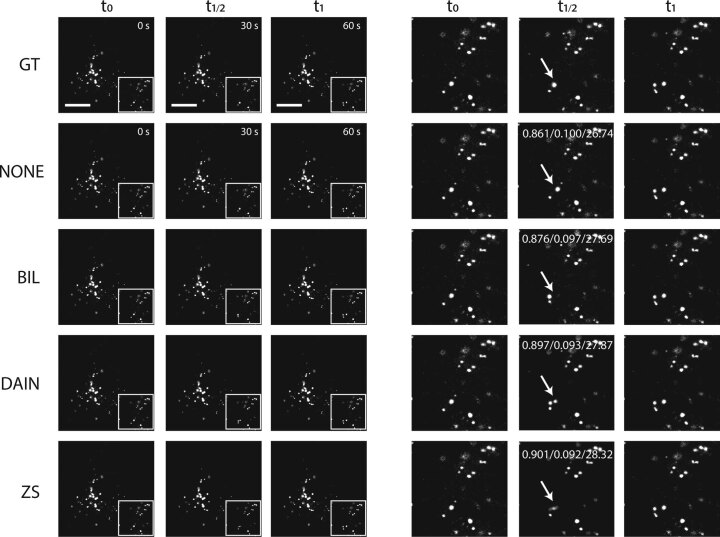
2x frame interpolation on moving lysosome of SHSY5Y cells. 2x frame interpolation of NONE, BIL, DAIN and ZS for lysosomes of SH-SY5Y cells recorded on a point-scanning confocal microscope. Overall ZS produces slightly better-quality results than DAIN even though ZS misses faster moving lysosomes which DAIN is able to capture well (example highlighted with white arrows in zoomed-in sections on the right). SSIM/RMSE/PSNR values displayed in the zoomed-in interpolated images. CAFI tools clearly outperform NONE and BIL interpolations. Scale bar: 10 *μ*m.

**Extended Data Fig. 8:**
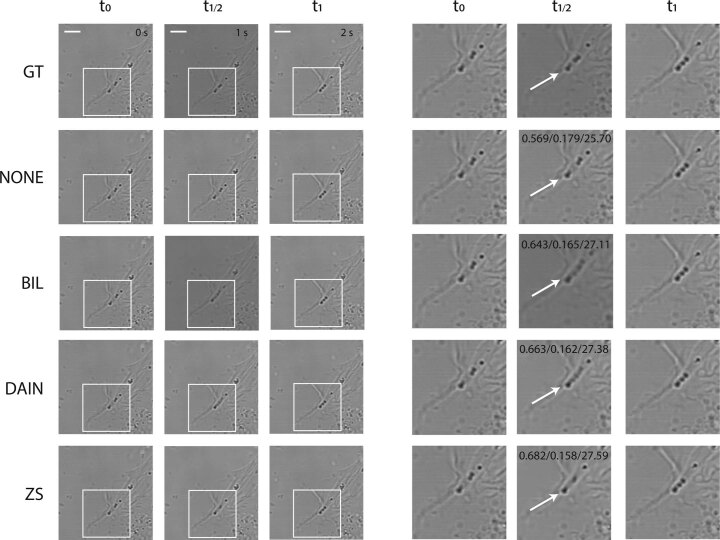
2x frame interpolation on brightfield image of SHSY5Y cells. 2x frame interpolation with NONE, BIL, DAIN and ZS for SH-SY5Y cells recorded on a confocal brightfield microscope. ZS produces slightly better-quality results than DAIN. DAIN creates visual artifacts for fast moving lipid droplets while ZS manages to capture the movement better (see white arrows in zoomed-in sections on the right). SSIM/RMSE/PSNR values displayed in the zoomed-in interpolated images. CAFI tools clearly outperform NONE and BIL interpolations. Scale bar: 10 *μ*m.

**Extended Data Fig. 9:**
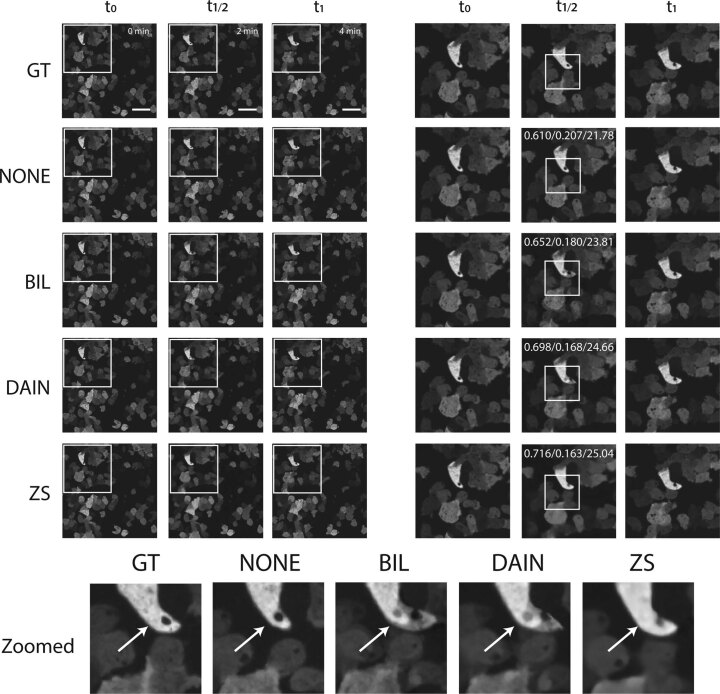
2x frame interpolation on moving Dictyostelium discoideum cells. 2x image frame interpolation of NONE, BIL, DAIN and ZS for *Dictyostelium discoideum* cells recorded on a spinning-disk confocal microscope. ZS creates smoother transitions and better-quality interpolation results than DAIN (see white arrow in zoomed-in section). SSIM/RMSE/PSNR values displayed in the zoomed-in interpolated images. CAFI tools clearly outperform NONE and BIL interpolations. Scale bar: 20 *μ*m.

**Extended Data Fig. 10:**
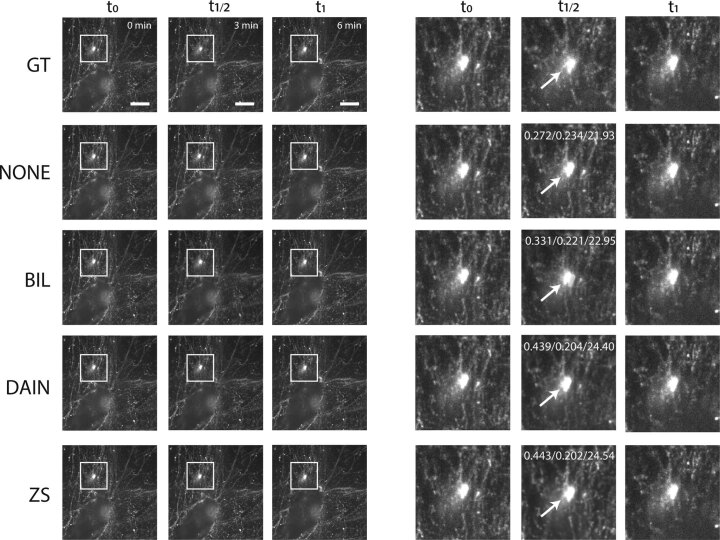
2x frame interpolation on fibronectin labelled A2780 cells. 2x image frame interpolation of NONE, BIL, DAIN and ZS for fibronectin labelled A2780 cells recorded on a spinning-disk confocal microscope. ZS creates smoother transitions and better-quality interpolation results than DAIN (see white arrow in zoomed-in sections on the right). SSIM/RMSE/PSNR values displayed in the zoomed-in interpolated images. CAFI tools clearly outperform NONE and BIL interpolations. Scale bar: 10 *μ*m. Data from Kaukonen et al.^38^.

Source Data Extended Data Fig. 5 SSIM, RMSE and PSNR of mitochondria frame interpolation comparison for the different interpolation techniques.

## Data Availability

Example training data and pretrained models are included in the GitHub release (v1.0.0). Our training and testing datasets as well as our source data files from our experiments are made available via Zenodo (10.5281/zenodo.10076346). The Vimeo90K dataset used for pretraining the ZS network can be downloaded from http://data.csail.mit.edu/tofu/dataset/vimeo_triplet.zip. The mitochondria source data are made available at Texas Data Repository 10.18738/T8/YLCK5A as provided by Fang et al.^30^ Source data are provided with this paper.

## Code availability

The CAFI source code and documentation (of ZS and DAIN) are available for download on GitHub (https://github.com/mpriessner/CAFI) and are free for nonprofit use.
