# Supplementary material for: Content-aware frame interpolation (CAFI): deep learning-based temporal super-resolution for fast bioimaging
Source: Nat Methods. 2024 Jan 18;21(2):322–30. doi: 10.1038/s41592-023-02138-w (PMC10864186; doi:10.1038/s41592-023-02138-w)
Supplement: Supplementary file 1 — All supplementary information as a single PDF. [file 41592_2023_2138_MOESM1_ESM.pdf]

nature methods

Article

https://doi.org/10.1038/s41592-023-02138-w

Content-aware frame interpolation (CAFI): deep learning-based temporal super-resolution for fast bioimaging

In the format provided by the authors and unedited

Content-aware frame interpolation (CAFI): Deep learning-based temporal super-resolution for fast bioimaging - Supplementary Information

Martin Priessner, David C.A. Gaboriau, Arlo Sheridan, Tchern Lenn, Carlos Garzon-Coral, Alexander R. Dunn, Jonathan R. Chubb, Aidan M. Tousley, Robbie G. Majzner, Uri Manor, Ramon Vilar, Romain F. Laine

Supplementary Note 1: Links to ZeroCostDL4Mic platform and to Google Colab notebooks

CAFI Github page:
https://github.com/mpriessner/CAFI
ZeroCostDL4Mic Wiki:
https://github.com/HenriquesLab/ZeroCostDL4Mic/wiki
DAIN 4 Microscopy Notebook:
https://colab.research.google.com/drive/1bL6wgTWrghHK7LH9xb4KGSk5WuOa5nJS?usp=sharing
ZoomingSlowMo 4 Microscopy Notebook:
https://colab.research.google.com/drive/1TZ0K-rq9Nrgu9_XZ0UOK6brxjIM0ISNU?usp=sharing

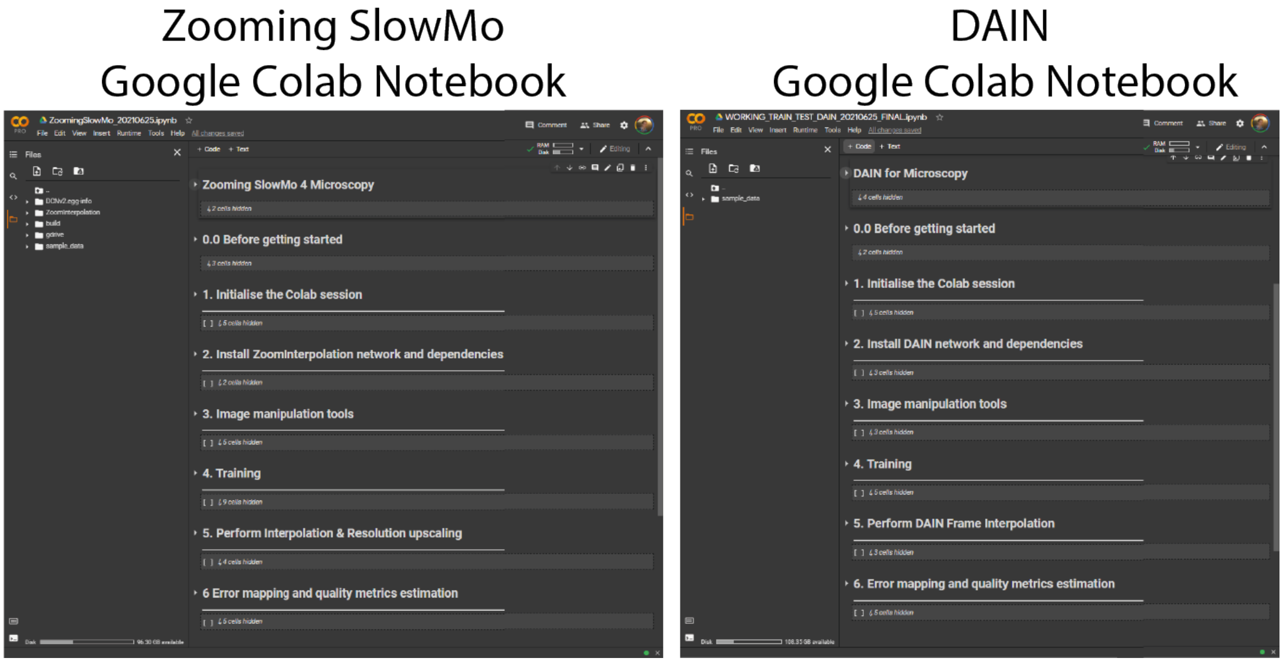

Screenshots of the two ZeroCostDL4Mic Google Colab notebooks for DAIN and ZS.

Supplementary Note 2: Image upscaling using Zooming SlowMo CAFI network

On top of ZS' interpolation functionality, it also has the ability to simultaneously increase the lateral image resolution while performing the temporal or axial content-aware interpolation. The 2x and 4x lateral upsampling capabilities of ZS were compared with BIL and BIC upsampling and one other lateral upsampling neural network called SRFBN-S which showed state-of-the-art competitive results in the so called "Image super-resolution" upsampling task https://github.com/Paper99/SRFBN$_{C}VPR$19. The ZS and SRFBN-S neural networks were fine-tuned on training data of the same imaging modality (for more details on the training see Supplementary Table S4). ZS clearly outperforms BIL and BIC upsampling and creates better results compared to the SRFBN-S neural network (see Supplementary Figures S27-S32). Finally, we compared the lateral upsampling functionality of ZS with the 4x upsampling results of the provided noisy electron microscopy images of the recently published PSSR network (39). Here ZS was fine-tuned on the training data with added gaussian noise as it was done in the original paper for PSSR. ZS produced clearly better results compared to BIC/BIL and also equivalent results compared to PSSR (see examples in Supplementary Figure S33 and delta comparisons in Supplementary Figure S34). The full quality evaluation results (SSIM, RMSE, PSNR) for 2x and 4x lateral upsampling are presented in Supplementary Table S5 and S6.

Supplementary Videos

Supplementary Video S1: Demonstration of CAFI networks for temporal interpolation on point-scanning confocal microscopy dataset of fluorescently labelled mitochondria branches (data from Fang *et al.* (30)). Scale bars correspond to 5 µm and 2.5 µm for the full and zoomed-in video, respectively.

Supplementary Video S2: Demonstration of CAFI networks for temporal interpolation of a simulated particles dataset generated with the ISBI particle tracking challenge plugin from Icy (34). Particle diameter is 15 pixels and the selected movement velocities are ranging from V2 to V8.

Supplementary Video S3: Demonstration of TrackMate (35) tracking improvements of simulated particles generated with the ISBI particle tracking challenge plugin from Icy (24) after temporal interpolation with CAFI. Particle diameter is 15 pixels and the movement velocities selected ranging from V2 to V8.

Supplementary Video S4: Demonstration example of lysosomal tracking improvements after temporal interpolation with CAFI using TrackMate (35). Lysosomes were labelled with FLCS1 (42) and the image sequence was collected as 3D+t dataset on a Leica SP5 with a 63x magnification 1.4 NA oil objective. Z-stacks were projected with maximum intensity generating a 2D+t dataset. The tracking results from the full image sequence was taken as ground truth tracks for quality comparison. Scale bars correspond to 20 µm and 6 µm for the full and zoomed-in video, respectively.

Supplementary Video S5: Demonstration of CAFI networks for temporal interpolation on inverted spinning-disk confocal microscopy dataset of GFP labelled *Dictyostelium discoideum* cells. Images were recorded using a 63x 1.4 NA oil objective with 2-minute frame intervals. Scale bars correspond to 10 µm and 4 µm for the full and zoomed-in video, respectively.

Supplementary Video S6: Demonstration of CAFI networks for temporal interpolation on a spinning-disk confocal microscopy dataset of fluorescently labelled fibronectin of A2780 cells (data from Kaukonen *et al.* Scale bars correspond to 10 µm and 3 µm for the full and zoomed-in video, respectively.(38)).

Supplementary Video S7: Demonstration of CAFI networks for temporal interpolation on confocal brightfield dataset of SH-SY5Y cells. Images were recorded on a Leica SP5 with a 63x magnification 1.4 NA oil objective with 1-second frame intervals. The image sequence was downsampled removing every second image in two iterative steps and re-interpolated in temporal dimension with 2x CAFI and 4x iCAFI. Scale bars correspond to 10 µm and 4 µm for the full and zoomed-in video, respectively.

Supplementary Video S8: Demonstration of CAFI networks for temporal interpolation on a point-scanning confocal microscopy dataset of fluorescently labelled lysosomes of SH-SY5Y cells. Images were recorded on a Leica SP5 with a 63x magnification 1.4 NA oil objective with 1-second frame intervals. The image sequence was downsampled removing every second image in two iterative steps and re-interpolated in temporal dimension with 2x CAFI and 4x iCAFI. Scale bars correspond to 5 µm and 2 µm for the full and zoomed-in video, respectively.

Supplementary Video S9: Demonstration of zCAFI networks for axial interpolation on an electron microscopy dataset of rat hippocampus (data from Fang *et al.* (30)). The image sequence was downsampled removing every second image in two iterative steps and re-interpolated in axial dimension with 2x CAFI and 4x iCAFI. Scale bars correspond to 0.2 µm and 0.08 µm for the full and zoomed-in video, respectively.

Supplementary Video S10: Demonstration of zCAFI networks for axial interpolation on a structured illumination microscopy (SIM) dataset of fluorescently labelled actin of DCIS.COM cells (data from Kaukonen *et al.* (38)). Scale bars correspond to 10 µm and 2 µm for the full and zoomed-in video, respectively.

Supplementary Video S11: Demonstration of tzCAFI on fluorescently labelled fibronectin of A2780 cells on a 4D spinning-disk confocal microscopy dataset (data from Kaukonen *et al.* (38)). The networks were trained for the interpolation task on data in the temporal dimension and the same fine-tuned network was used for both interpolation dimensions (axial and temporal). Scale bars correspond to 5 µm.

Supplementary Video S12: Demonstration of tzCAFI on *C. elegans* embryo labeled with GFP targeting alpha tubulin recorded on a 4D lattice light sheet microscope. Due to data limitation the networks were just fine-tuned on augmented (90°, 180°, 270° rotated and mirrored) images of the tested dataset. This resulted in improvements for ZS but not for DAIN where the pretrained network showed already improved performance and was finally used for this comparison.

Supplementary Video S13: Demonstration of tzCAFI on CAR-T cells recorded on a 4D lattice light sheet microscope. Scale bars correspond to 8 µm. Due to data limitation, the networks were just fine-tuned on augmented (90°, 180°, 270° rotated and mirrored) images of the tested dataset. This resulted in improvements for ZS but not for DAIN where the pretrained network showed already improved performance and was finally used for this comparison.

Supplementary Video S14: Demonstration of tzCAFI on MDCK cells recorded on a 4D lattice light sheet microscope. Scale bars correspond to 8 µm. Due to data limitation, the networks were just fine-tuned on augmented (90°, 180°, 270° rotated and mirrored) images of the tested dataset. This resulted in improvements for ZS but not for DAIN where the pretrained network showed already improved performance and was finally used for this comparison.

Supplementary Video S15: Demonstration of tzCAFI on *C. elegans* embryo labeled with GFP targeting GFP-PH recorded on a 4D lattice light sheet microscope. Scale bars correspond to 8 µm. Due to data limitation, the networks were just fine-tuned on augmented (90°, 180°, 270° rotated and mirrored) images of the tested dataset. This resulted in improvements for ZS but not for DAIN where the pretrained network showed already improved performance and was finally used for this comparison.

Supplementary Video S16: Demonstration of tzCAFI on fluorescently labelled lysosomes in SHSY-5Y cells recorded on recorded on a confocal microscop. Scale bars correspond to 8 µm. Due to data limitation, the networks were just fine-tuned on augmented (90°, 180°, 270° rotated and mirrored) images of the tested dataset. For this dataset fine-tuning did not improve the performance of the networks and therefore the already well performing pretrained networks were used for this comparison.

Supplementary Tables and Figures

ICY (ISBI Challenge Track Generator)	
	**T-Dimension Dataset**
**SNR:**	10
**Density:**	40
**Image width:**	512
**Image height:**	512
**Image depth:**	1
**Sequence length:**	225
**MinTrackLength:**	52
**Warmup length:**	50
**Extinction rate:**	0.003
**x borders simu:**	50
**y borders simu:**	50
**z borders simu:**	20
**xy pixel size:**	15
**Slice spacing:**	250
**Creator types:**	SWITCHING_UNIFORM
**Sigma Brownian:**	1
**q1:**	2
**V min:**	2
**V max:**	2/10*
**Probe directed Brownian:**	0.3
**Probe Brownian directed:**	0.3

Table S1. Parameter table for creating simulated datasets with ICY plugin (40). The * mark at parameter V max indicated the range of speeds selected for the training data generation.

2x temporal interpolation																							
			2x-NONE			2x-BIC			2x-BIL			2x-DAIN			2x-DAIN (FT)			2x-ZS			2x-ZS (FT)		
	Dim	Image nr.	SSIM	RMSE	PSNR	SSIM	RMSE	PSNR	SSIM	RMSE	PSNR	SSIM	RMSE	PSNR	SSIM	RMSE	PSNR	SSIM	RMSE	PSNR	SSIM	RMSE	PSNR
Dictyostelium Data	T	51	0.744	0.160	26.750	0.816	0.121	37.107	0.815	0.124	35.815	0.856	0.096	42.981	0.856	0.096	43.007	0.866	0.095	42.517	**0.867**	0.094	42.917
SH-SY5Y Data	T	63	0.778	0.089	78.786	0.800	0.107	40.953	0.803	0.114	37.792	0.828	0.101	42.051	0.829	0.101	42.057	0.832	0.100	41.429	**0.835**	0.099	42.113
Synthetic Data	T	113	0.748	0.104	41.014	0.762	0.112	35.975	0.766	0.114	36.499	0.808	0.095	42.598	0.810	0.095	42.619	0.818	0.094	42.651	**0.820**	0.093	42.555
Lysosomal Data	T	57	0.922	0.049	80.319	0.926	0.061	41.127	0.924	0.060	40.900	0.935	0.046	81.321	0.940	0.045	81.237	0.941	0.047	53.423	**0.942**	0.046	54.979
Mitochondrial Data	T	97	0.891	0.101	40.834	0.899	0.107	35.580	0.902	0.107	35.827	0.914	0.094	41.203	0.916	0.094	41.230	0.918	0.094	41.150	**0.921**	0.093	41.310
Fibronectin Data	T	13	0.663	0.108	78.749	0.680	0.128	42.939	0.682	0.130	42.188	0.732	0.104	46.883	0.733	0.104	46.896	0.732	0.103	46.617	**0.734**	0.104	45.067

Table S2. Quality evaluation of NONE, BIC, BIL, DAIN, FT-DAIN, ZS and FT-ZS interpolation in temporal dimension on 6 different datasets. The numbers in red and blue indicate the best and second-best performance.

2x axial interpolation																							
			2x-NONE			2x-BIC			2x-BIL			2x-DAIN			2x-DAIN (FT)			2x-ZS			2x-ZS (FT)		
	Dim	Images nr.	SSIM	RMSE	PSNR	SSIM	RMSE	PSNR	SSIM	RMSE	PSNR	SSIM	RMSE	PSNR	SSIM	RMSE	PSNR	SSIM	RMSE	PSNR	SSIM	RMSE	PSNR
Fibronectin Data	Z	13	0.679	0.123	40.273	0.714	0.128	36.798	0.715	0.128	36.422	0.732	0.112	41.386	0.733	0.111	41.409	0.701	0.114	41.025	**0.752**	0.108	41.653
Actin Data	Z	33	0.769	0.127	35.265	0.813	0.126	33.219	0.807	0.130	32.395	0.810	0.105	40.077	0.812	0.105	40.105	0.812	0.103	40.180	**0.870**	0.093	41.984
Hippocampus Data	Z	97	0.748	0.134	39.966	0.775	0.146	34.249	0.780	0.148	33.565	0.801	0.121	41.449	0.802	0.120	41.483	0.806	0.119	41.587	**0.811**	0.118	41.698

Table S3. Quality evaluation of NONE, BIC, BIL, DAIN, FT-DAIN, ZS and FT-ZS interpolation on 3 different datasets in axial dimension. The numbers in red and blue indicate the best and second-best performance.

Training / fine tuning for lateral upscaling								
Dataset	Microscopy type	Upscale factor	LR pixel dimension	HR pixel dimension	Size HR	epochs SRFBN|niter ZS	LR SRFBN|ZS	Batch size SRFBN|ZS
VIMEO video data	-	2x	488x256	244x128	82 GB	- |600000	- |1e-5	- |16
VIMEO video data	-	4x	488x256	122x64	82 GB	- | provided	- | provided	- | -
SH-SY5Y data	Confocal brightfield microscope	2x	1024x1024	512x512	3.6 GB	10|20000	1e-4|1e-5	16|16
SH-SY5Y data	Confocal brightfield microscope	4x	1024x1024	512x512	3.6 GB	10|20000	1e-4|1e-5	16|16
Dictyostelium data	Spinning disc confocal microscope	2x	512x512	256x256	4.3 GB	10|20000	1e-4|1e-5	16|16
Dictyostelium data	Spinning disc confocal microscope	4x	512x512	128x128	4.3 GB	10|20000	1e-4|1e-5	16|16
Hippocampus data	Electron microscope	2x	512x512	256x256	12.1 GB	5|10000	1e-4|1e-5	16|16
Hippocampus data	Electron microscope	4x	512x512	128x128	12.1 GB	5|10000	1e-4|1e-5	16|16
Lysosomal data	Confocal microscope	2x	512x512	256x256	5.2 GB	10|20000	1e-4|1e-5	16|16
Lysosomal data	Confocal microscope	4x	512x512	128x128	5.2 GB	10|20000	1e-4|1e-5	16|16

Table S4. Training data and selected parameters for fine-tuning the ZS and SRFBN-S networks for the lateral upsampling task for the different datasets. The other network parameters were kept as default.

2x lateral upsampling							
**Dataset**	**Method**	**LR**	**HR**	**Slices**	**SSIM**	**RMSE**	**PSNR**
**SH-SY5Y data**	**ZS**	256	512	11	**0.9658**	0.0912	38.493
	**SRFBN-S**	256	512	11	0.9634	0.0925	38.135
	**BIC**	256	512	11	0.9564	0.1016	35.605
	**BIL**	256	512	11	0.9249	0.1200	32.631
**Dictyostel. data**	**ZS**	256	512	13	**0.7281**	0.1568	29.978
	**SRFBN-S**	256	512	13	0.7256	0.1571	29.932
	**BIC**	256	512	13	0.7054	0.1584	29.780
	**BIL**	256	512	13	0.6779	0.1609	29.481
**Lysosomal data**	**ZS**	256	512	11	**0.7714**	0.1432	26.270
	**SRFBN-S**	256	512	11	0.7631	0.1429	26.259
	**BIC**	256	512	11	0.7132	0.1500	25.492
	**BIL**	256	512	11	0.6889	0.1539	25.023

Table S5. Quality evaluation metrics (SSIM, RMSE, PSNR) for 2x lateral upsampling. The numbers in red and blue indicate the best and second-best performance, respectively.

.

4x lateral upsampling							
**Dataset**	**Method**	**LR**	**HR**	**Slices**	**SSIM**	**RMSE**	**PSNR**
**SH-SY5Y data**	**ZS**	256	1024	11	**0.8473**	0.1212	33.943
	**SRFBN-S**	256	1024	11	0.8423	0.1223	33.725
	**BIC**	256	1024	11	0.8252	0.1281	32.491
	**BIL**	256	1024	11	0.7855	0.1394	30.737
**Dictyostel. data**	**ZS**	128	512	13	**0.6193**	0.1676	28.736
	**SRFBN-S**	128	512	13	0.6188	0.1678	28.720
	**BIC**	128	512	13	0.6015	0.1702	28.407
	**BIL**	128	512	13	0.5762	0.1733	28.018
**Lysosomal data**	**ZS**	128	512	11	0.6367	0.1615	24.109
	**SRFBN-S**	128	512	11	**0.6375**	0.1612	24.102
	**BIC**	128	512	11	0.6227	0.1658	23.455
	**BIL**	128	512	11	0.6110	0.1692	23.069
**Hippo. data**	**ZS**	125	500	42	**0.4085**	0.2413	22.659
	**PSSR**	125	500	42	0.4076	0.2414	22.648
	**BIC**	125	500	42	0.3797	0.2603	21.371
	**BIL**	125	500	42	0.3980	0.2528	21.861

Table S6. Quality evaluation metrics (SSIM, RMSE, PSNR) for 4x lateral upsampling. The numbers in red and blue indicate the best and second-best performance, respectively. The EM lateral upsampling results were just available for the comparison with PSSR. SRFBN-S failed on this dataset due to mishandling of the introduced noise in the training data.

**TrackMate Parameters**	
Estimated blob diameter:	10 um
Threshold:	5
Selected Tracker:	Simple LAP tracker
Linking max distance:	25
Gap-closing max distance:	25
Gap-closing max frame gap:	2

Table S7. Parameter table for tracking of simulated particles with Fiji Trackmate plugin (35).

TrackMate Parameters Lyso-Dataset	
**Estimated blob diameter:**	3 µm
**Threshold:**	10
**Selected Tracker:**	Simple LAP tracker
**Quality particles**	Number calibrated to GT number
**Linking max distance:**	8
**Gap-closing max distance:**	8
**Gap-closing max frame gap:**	2

Table S8. Parameter table for tracking of lysosomal particles with Fiji Trackmate plugin (35).

List of possible applications of CAFI	
**Category**	**Description**
**Long term imaging**	CAFI allows for faster multi-color imaging whereby the training dataset can be generated by fast continuous imaging of individual colors and inference done on slower multi-color channels (especially for slow multi-color systems like turret changing systems)
**Faster multi-color imaging**	CAFI allows for faster multi-color imaging whereby the training dataset can be generated by fast continuous imaging of individual colors and inference done on slower multi-color channels (especially for slow multi-color systems like turret changing systems)
**Improve tracking performance**	The tracking performance can be improved which allows for smoother traces and better linking
**Increasing image density of 4D datasets**	CAFI can improve 3D+t datasets by requiring fewer T frames and Z slices. This is especially helpful for experiments that are looking at fast processes. This can either help to reduce phototoxic or can improve image density with the same overall phototoxicity.
**Increasing high throughput imaging**	Increased recording speed due to fewer T and Z images results in the possibility to image more wells in a shorter amount of time (more plates per hour of imaging)

Table S9. Table presenting a not exhaustive list of possible CAFI applications for microscopy.

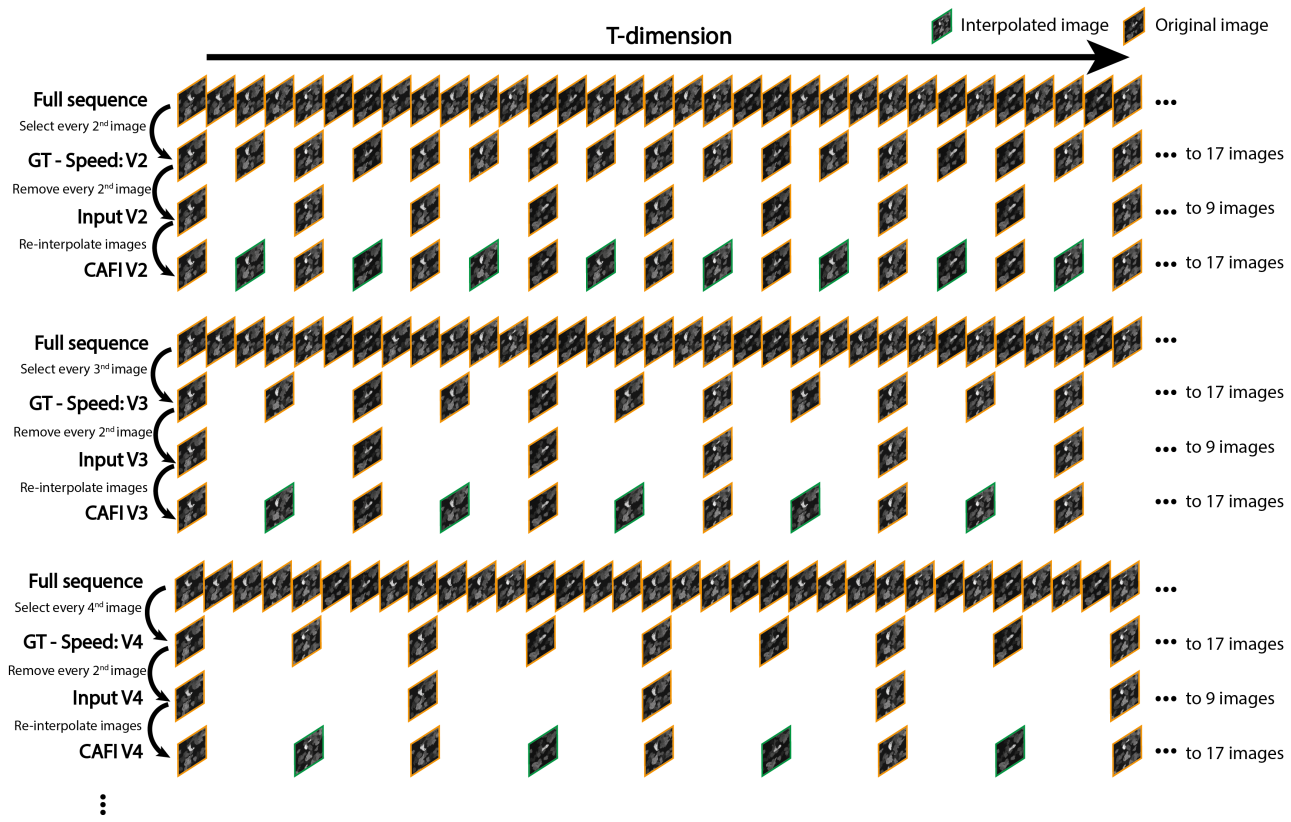

Fig. S1. Visual illustration of downsampling process to generate the ground truth (GT) for the different particle velocities. By removing an increasing number of images between two time points increased particle velocities are created as new GT datasets. Then every second image is removed and kept for quality evaluation and the downsampled sequence is then re-interpolated with the different interpolation techniques.

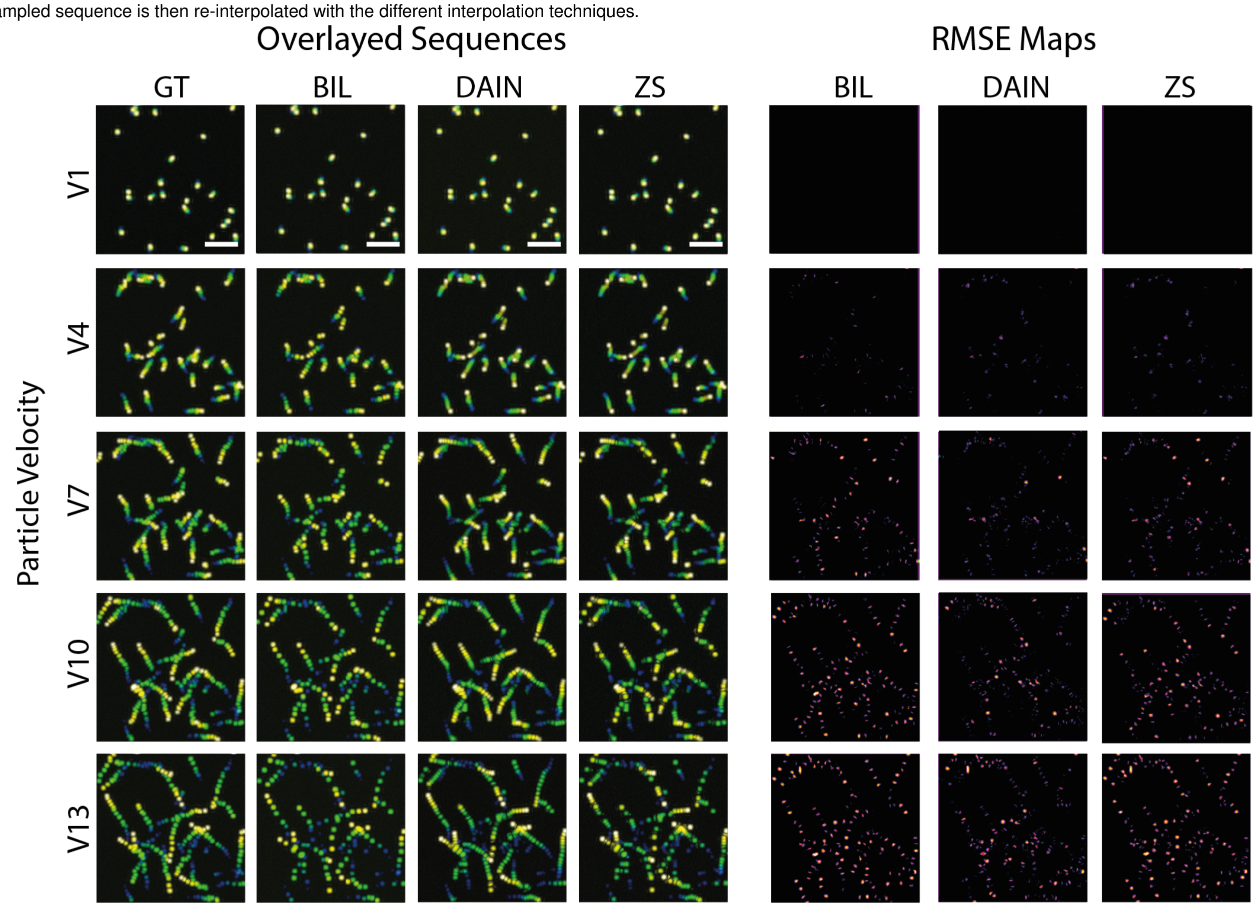

Fig. S2. Maximum intensity projection of the simulated time sequence dataset of the different image frame interpolation results (BIL, DAIN, ZS) at different particle movement velocities (left) with RMSE maps compared to ground truth (right). Scale bars: 50 pixels, n=17 frames.

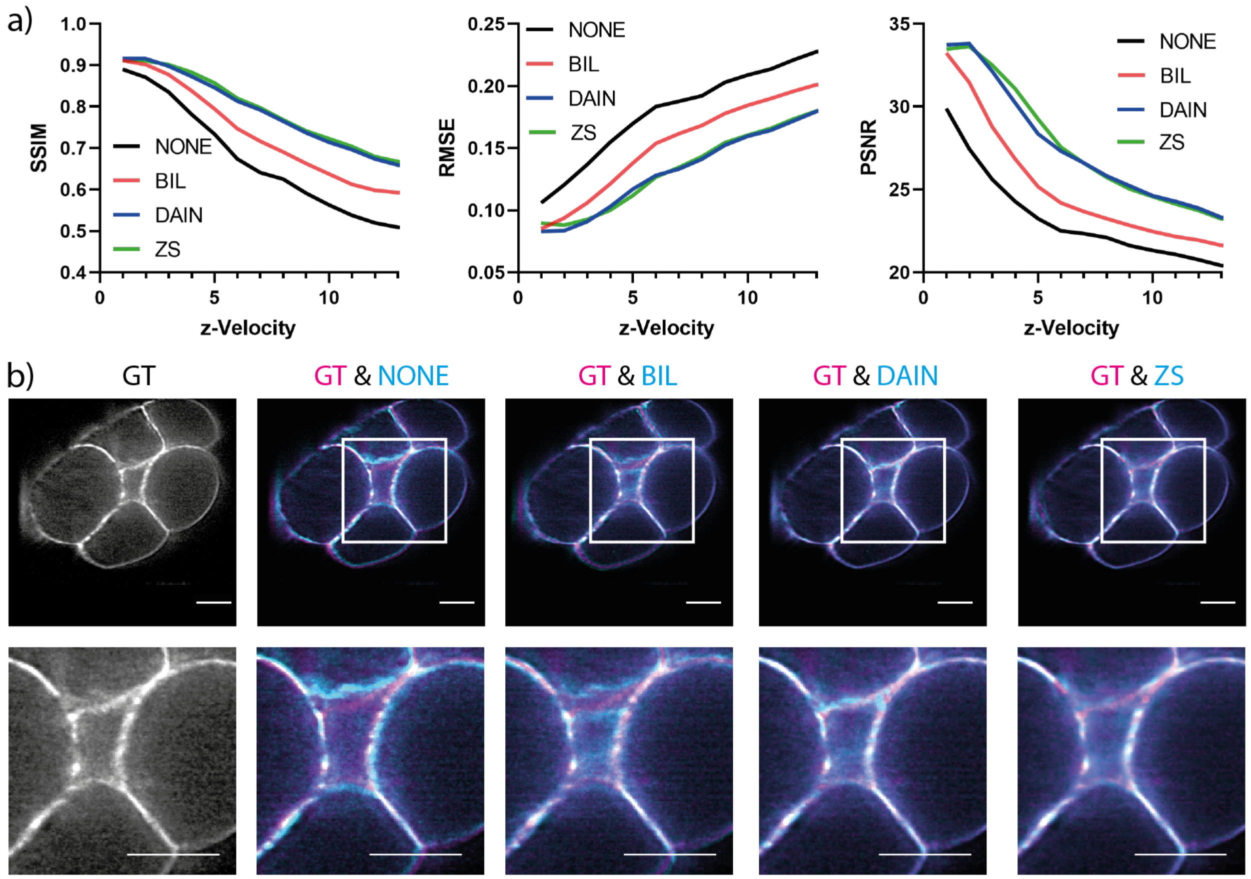

Fig. S3. Velocity interpolation in z-dimension: a) Image quality comparison of downsampled 3D *C. elegans* embryo light-sheet microscopy dataset in z-dimension. Between 1 and 15 images were removed between two frames in z-dimension followed by another downsampling step by a factor of 2. Then the sequences were re-interpolated with BIL, NONE, DAIN and ZS and the image quality (SSIM, RMSE, PSNR) of the interpolated images was compared to the ground truth datasets (n=10 interpolated images). b) Representative overlay example image of the interpolation result at interpolation velocity V7 (ground truth: magenta, interpolation technique: cyan; scale bar: 10 µm;).

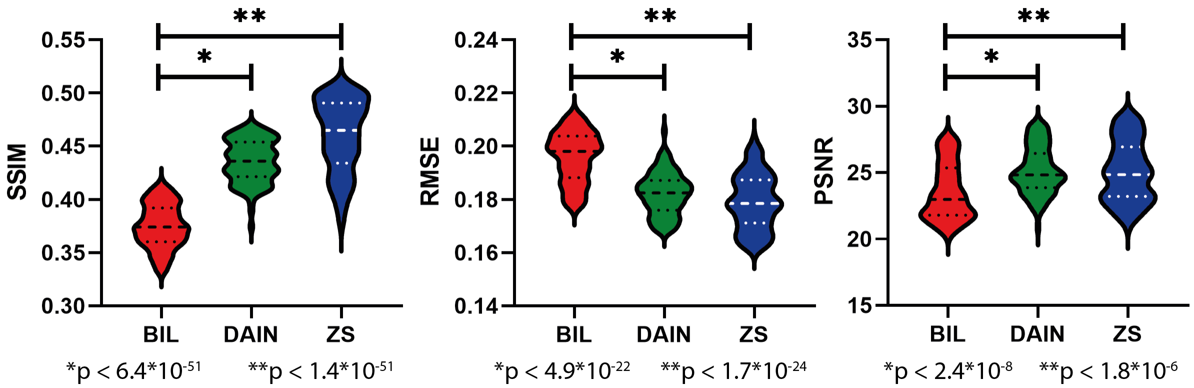

Fig. S4. Statistical analysis (two-sided student t-test) of the image quality metrics (SSIM, RMSE, PSNR) by comparing the values of DAIN and ZS of each interpolated image for all particle velocities with the quality metric results of BIL, showing strong significance of improvements for DAIN and ZS over BIL interpolation.

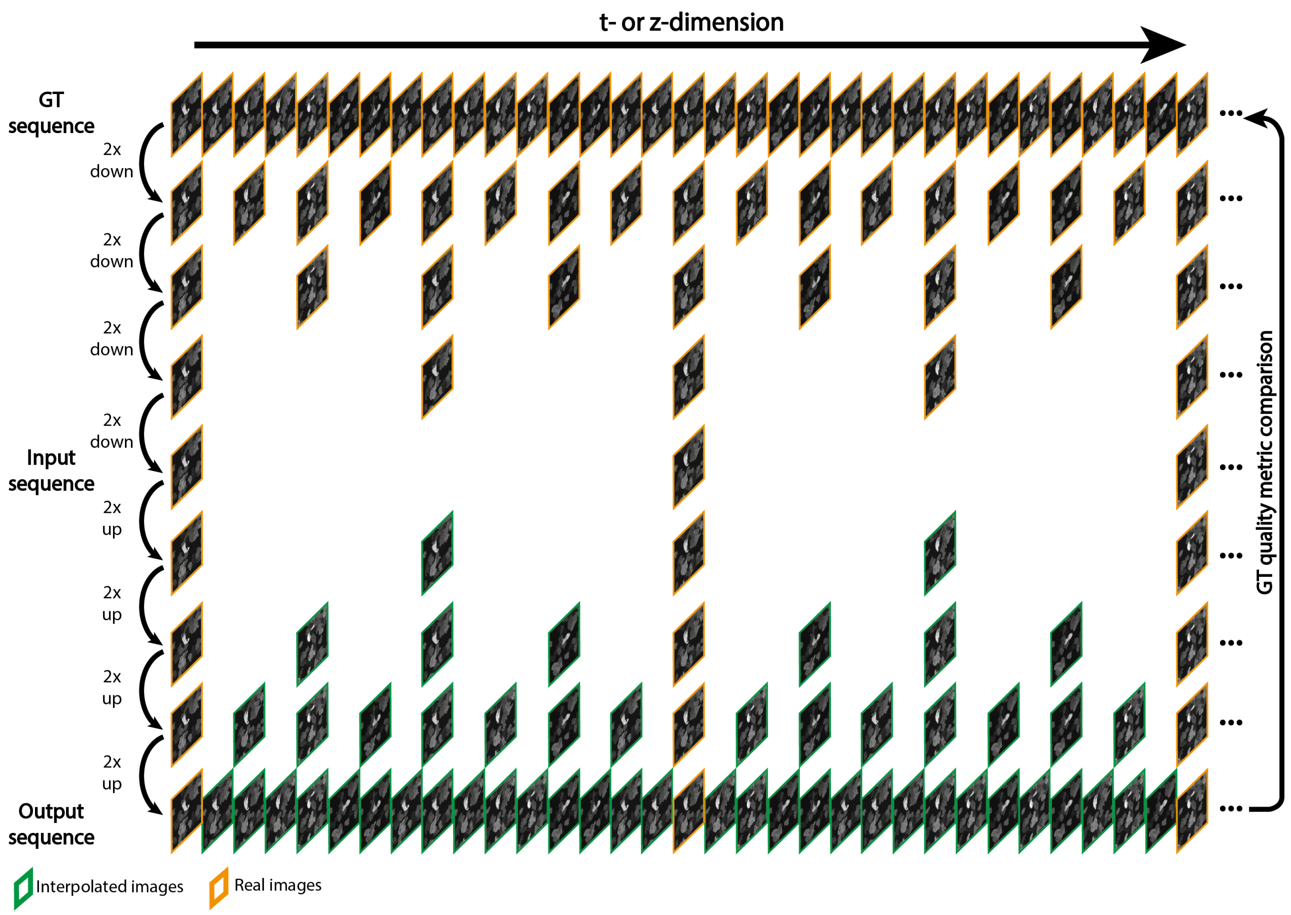

Fig. S5. Visual illustration of multi-step frame down- and upsampling for iCAFI. In several iterative steps every second image gets removed and is later re-interpolated in several steps reconstructing the high temporal frequency of the ground truth image sequence.

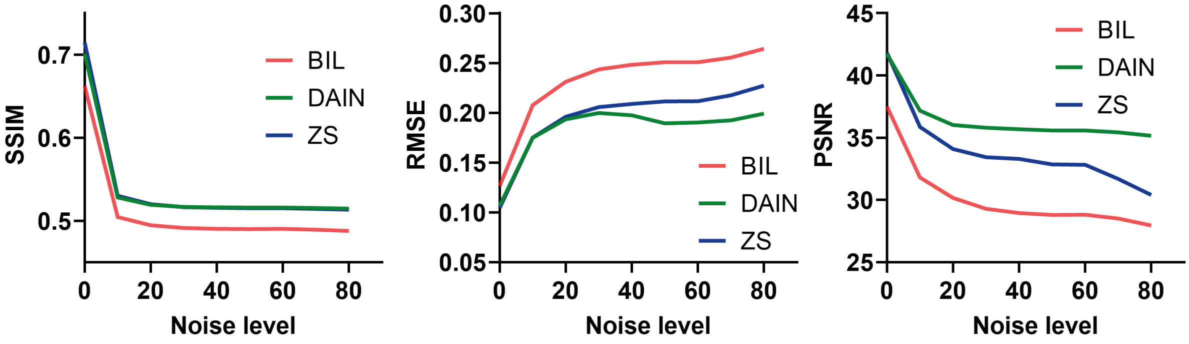

Fig. S6. Image quality assessment of noised simulated particle image sequences. Image quality metrics comparison (SSIM, RMSE, PSNR) of simulated noised dataset (standard deviation: 0-80) interpolation with BIL, DAIN and ZS with particle diameters of 15 pixels (n=45 images averaged).

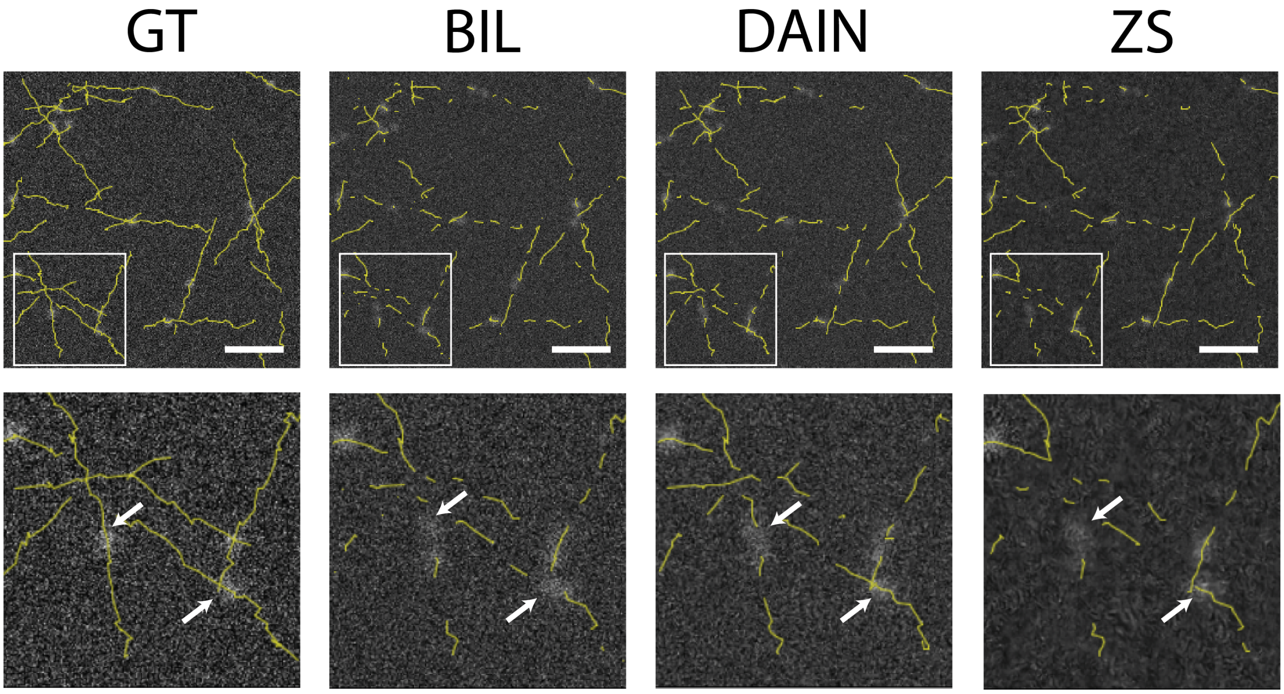

Fig. S7. Artifacts caused by reduced particle contrast. Simulated particle dataset generated with ICY track generator (49) at the noise level 40 created were interpolated with BIL, DAIN and ZS and particles with reduced contrast at this and higher noise levels caused a degrading tracking quality. White arrows highlight low contrast particles in interpolated images. Scale bars: 100 px.

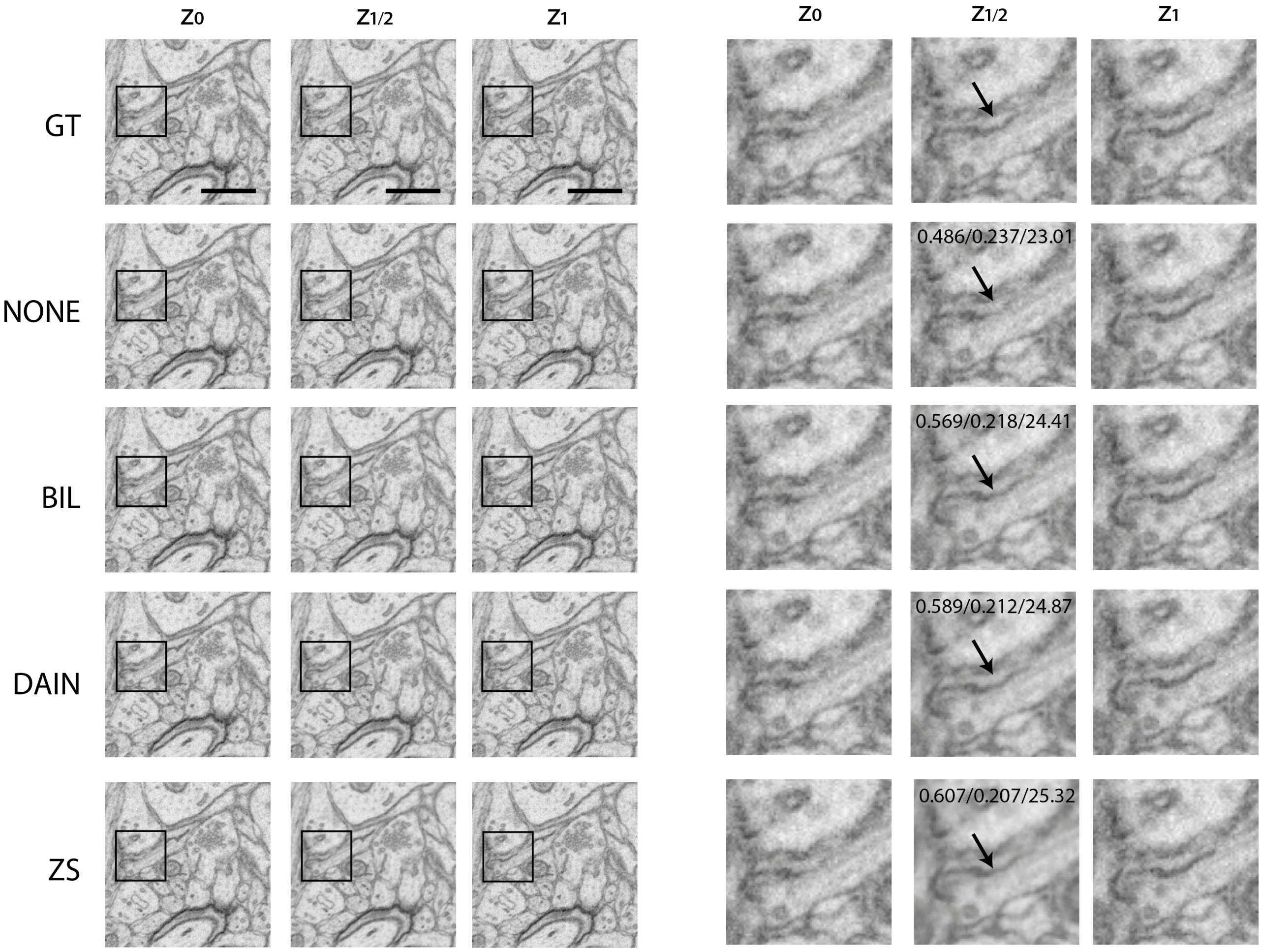

Fig. S8. 2x image slice interpolation in axial dimension with NONE, BIL, DAIN and ZS for rat hippocampus tissue recorded on an electron microscope. ZS produces better quality results than DAIN. ZS creates smoother transitions of an imaged dendrite (see black arrow in zoomed-in sections on the right). SSIM/RMSE/PSNR values displayed in the zoomed-in interpolated images. CAFI tools significantly outperform NONE and BIL interpolations. Scale bar: 0.4 µm. Data from Fang *et al.* (30).

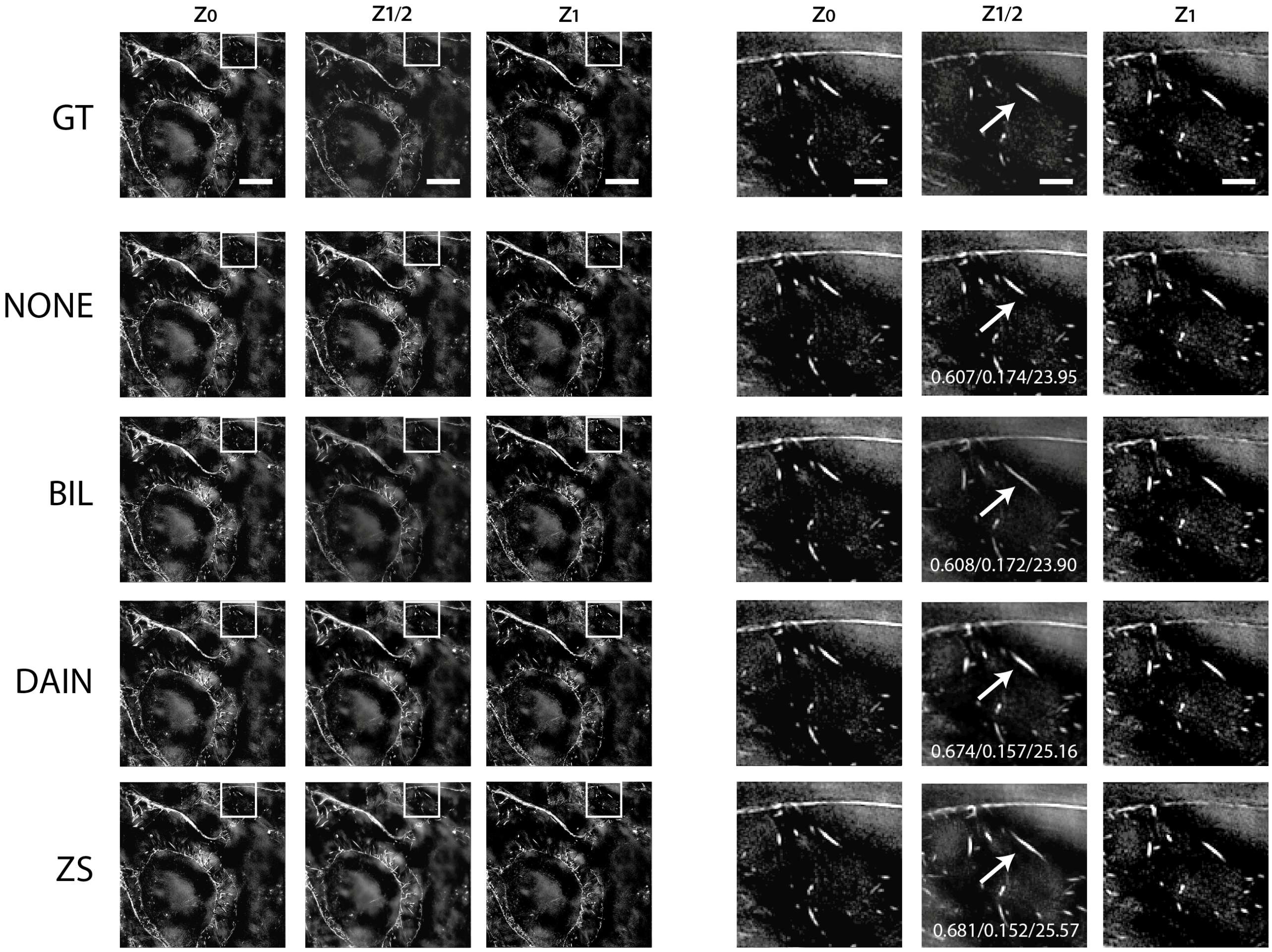

Fig. S9. 2x image slice interpolation in axial dimension of NONE, BIL, DAIN and ZS for actin labeled DCIS.COM cells recorded with a structured illumination microscope. ZS produces better quality results than DAIN and BIL. BIL interpolation results in a faulty elongation of the actin signal highlighted with white arrow. CAFI tools significantly outperform NONE, BIL interpolation. Scale bar: 10 µm. Data from Kaukonen *et al.* (38).

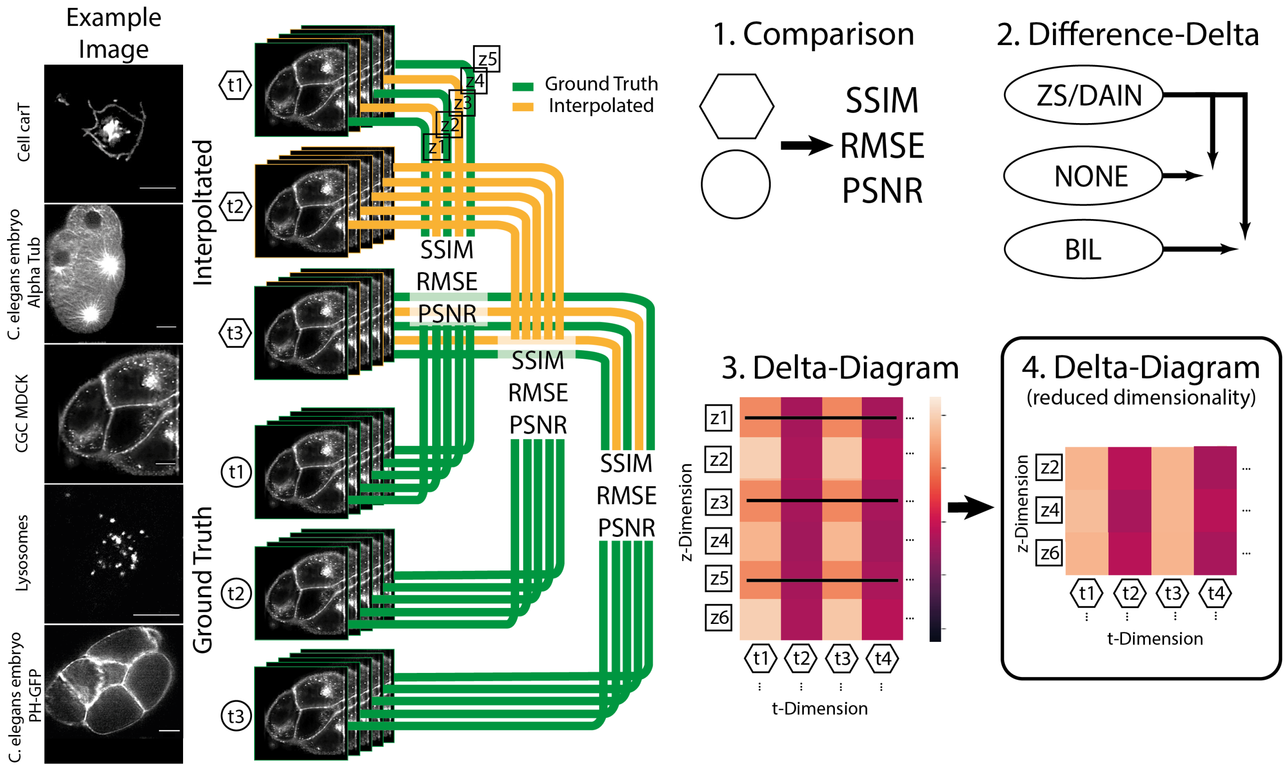

Fig. S10. Visualisation workflow for comparing neural network interpolations with classical interpolation results. First, the quality metrics (SSIM, RMSE, PSNR) were evaluated of all interpolation techniques for every image slice. Then the quality of each neural network image slice was compared with the classical interpolation results and the delta-value was visualized for each dataset as 2D heat maps. Every second z-slice (those that are identical to those in the ground truth dataset) was removed to show the differences more clearly on the interpolated images.

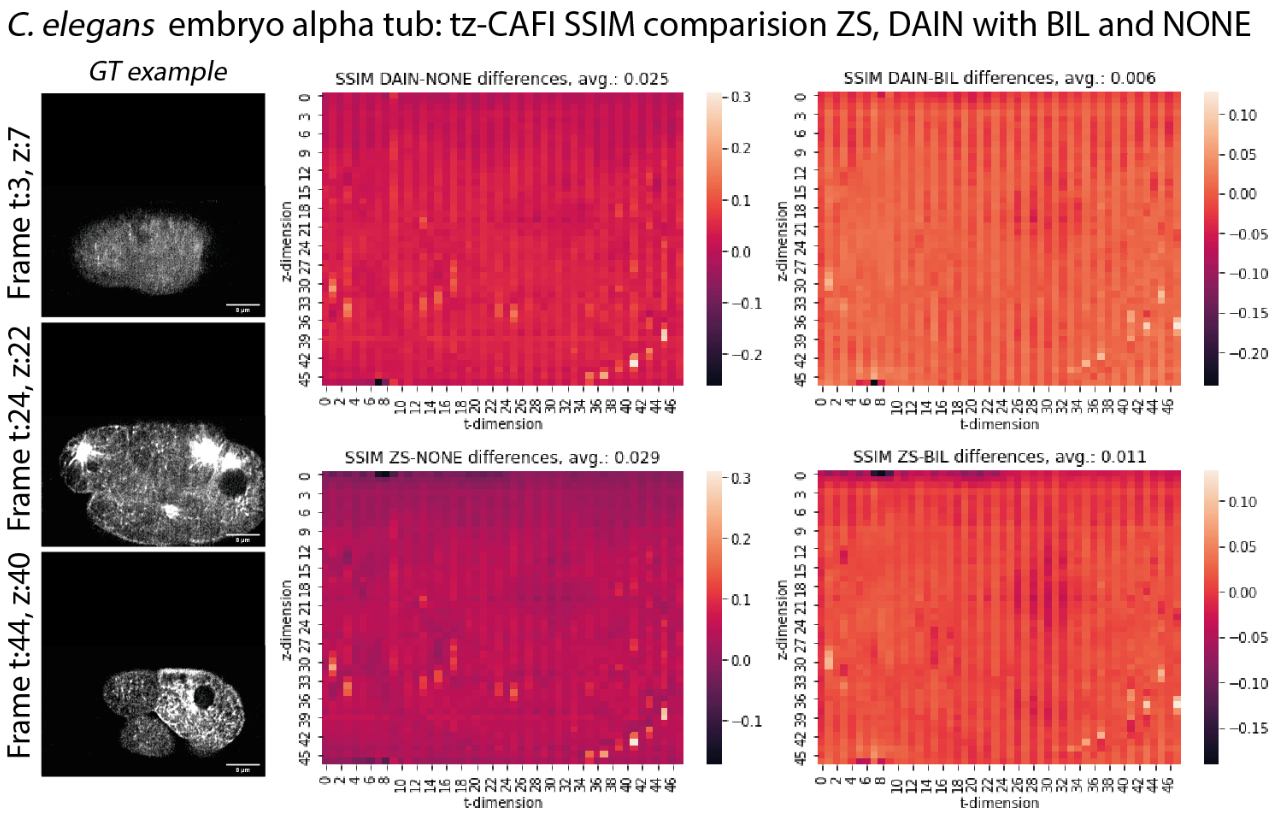

Fig. S11. Delta-SSIM quality comparison of DAIN and ZS compared to BIL and NONE of a 4D *C. elegans* embryo alpha tub dataset. Every second slice in the z-dimension was removed for this visualization (GT image dimensions: 47:97:512:512). The averaged similarity difference was calculated based on the entire dataset of all interpolated images across both t- and z-dimensions. Left panel shows example images of sample at different time points and z-dimensions. Scale bars: 8 µm.

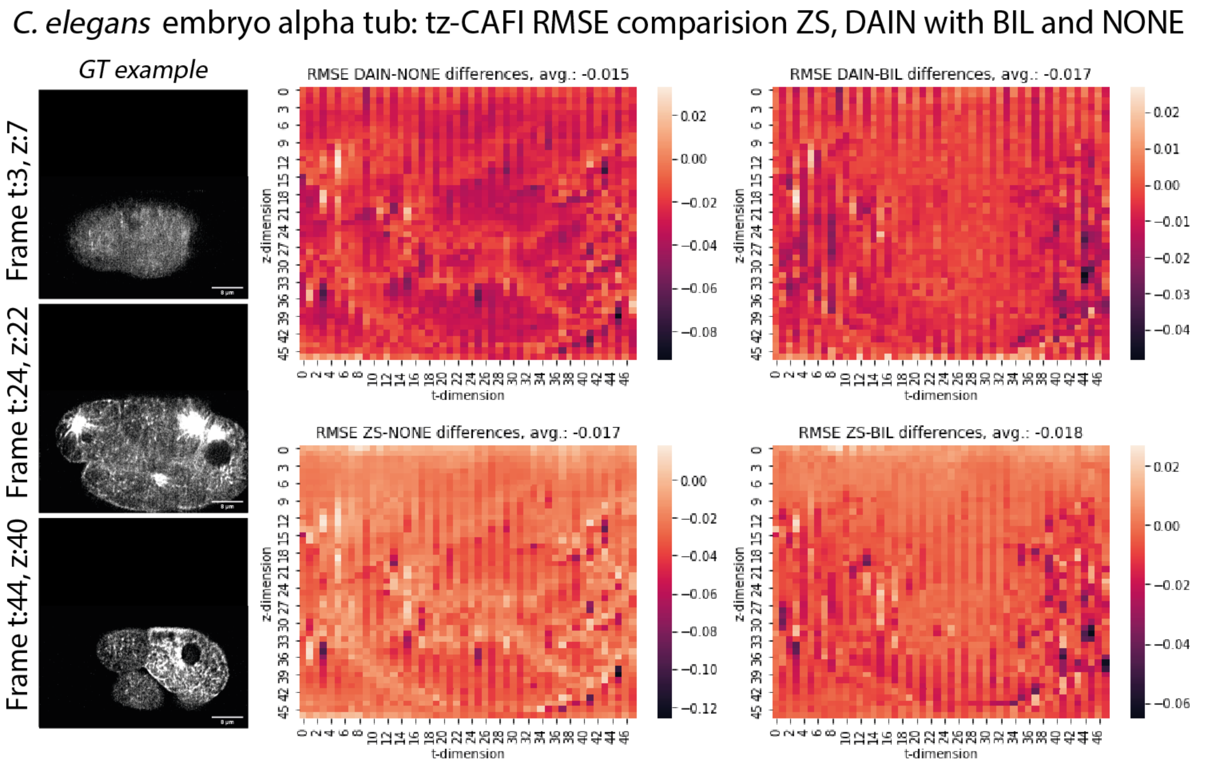

Fig. S12. Delta-RMSE quality comparison of DAIN and ZS compared to BIL and NONE of a 4D *C. elegans* embryo alpha tub dataset. Every second slice in the z-dimension was removed for this visualization (GT image dimensions:47:97:512:512). The averaged error difference was calculated based on the entire dataset of all interpolated images across both t- and z-dimensions. Left panel shows example images of sample at different time points and z-dimensions. Scale bars: 8 µm.

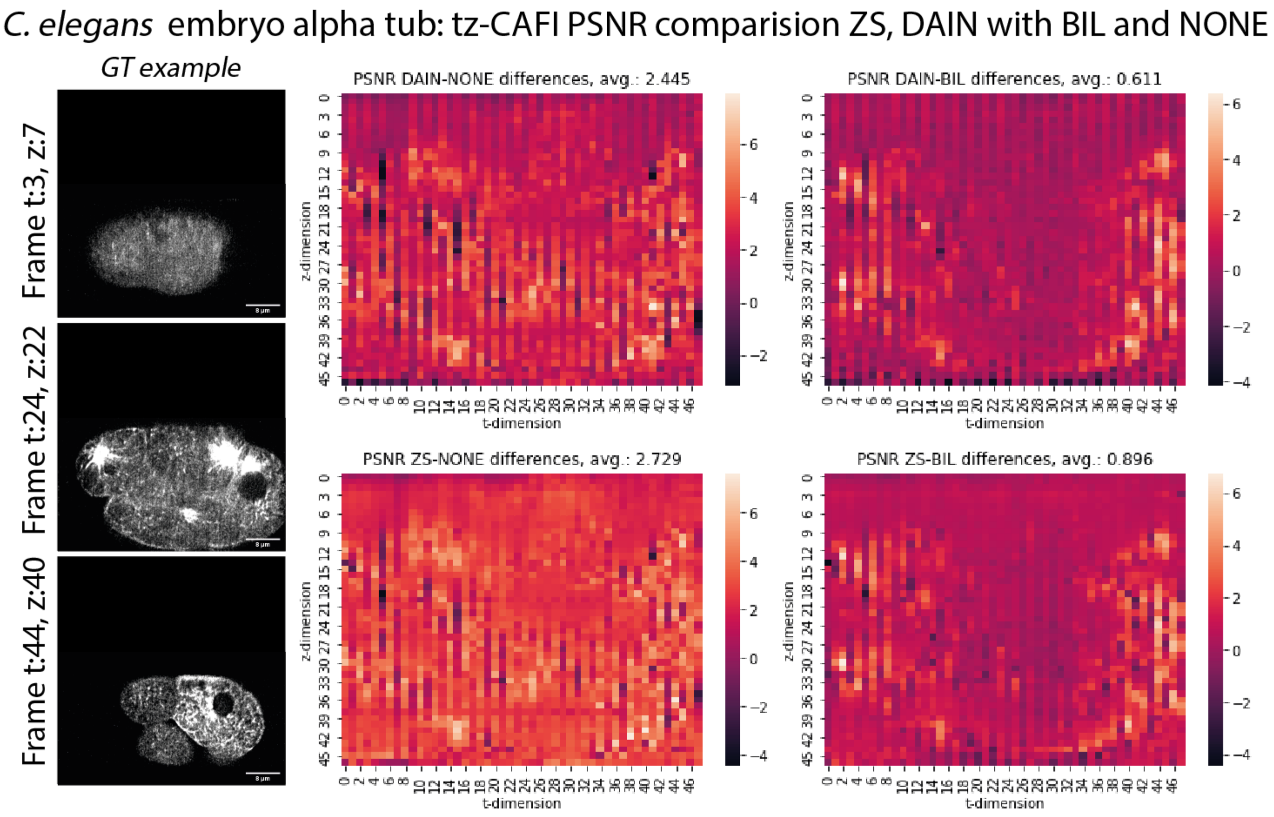

Fig. S13. Delta-PSNR quality comparison of DAIN and ZS compared to BIL and NONE of a 4D *C. elegans* embryo alpha tub dataset. Every second slice in the z-dimension was removed for this visualization (GT image dimensions:47:97:512:512). The averaged PSNR difference was calculated based on the entire dataset of all interpolated images across both t- and z-dimensions. Left panel shows example images of sample at different time points and z-dimensions. Scale bars: 8 µm.

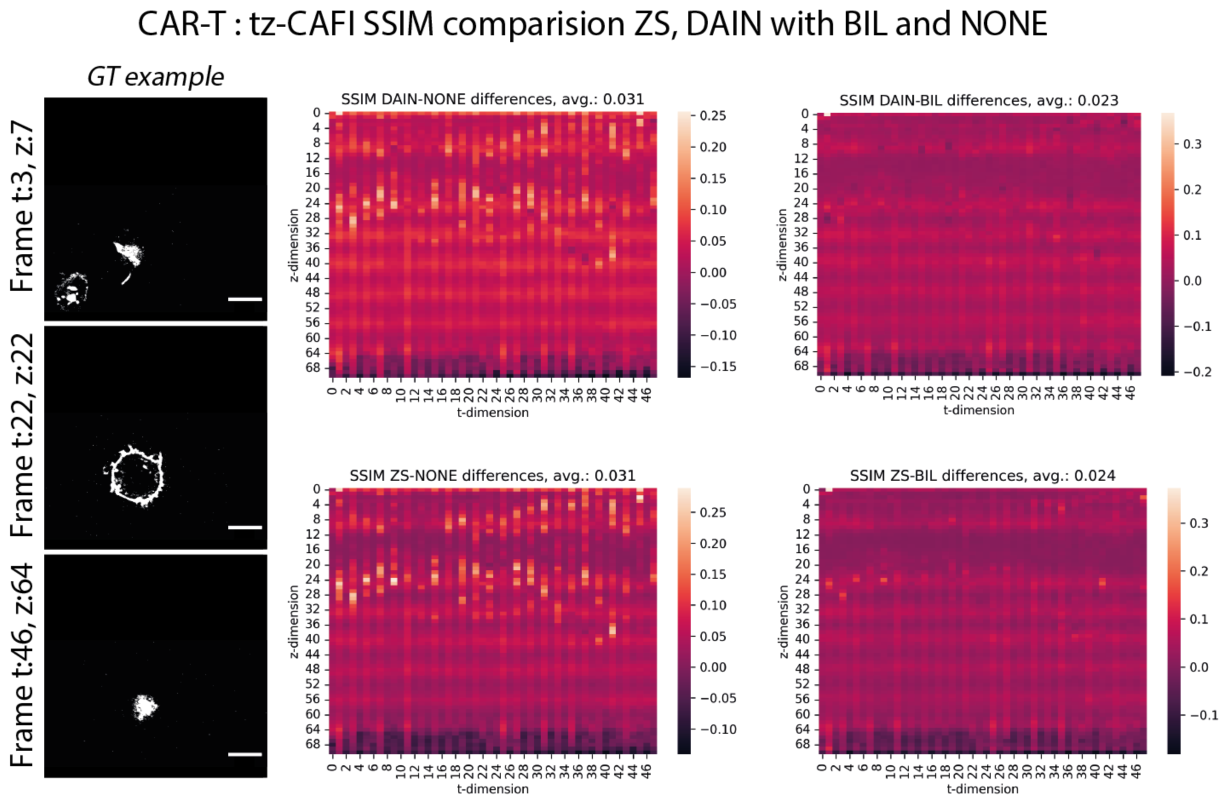

Fig. S14. Delta-SSIM quality comparison of DAIN and ZS compared to BIL and NONE of a 4D CAR-T cell dataset. Every second slice in the z-dimension was removed for this visualization (GT image dimensions: 47:115:512:512). The averaged similarity difference was calculated based on the entire dataset of all interpolated images across both t- and z-dimensions. Left panel shows example images of sample at different time points and z-dimensions. Scale bars: 8 µm.

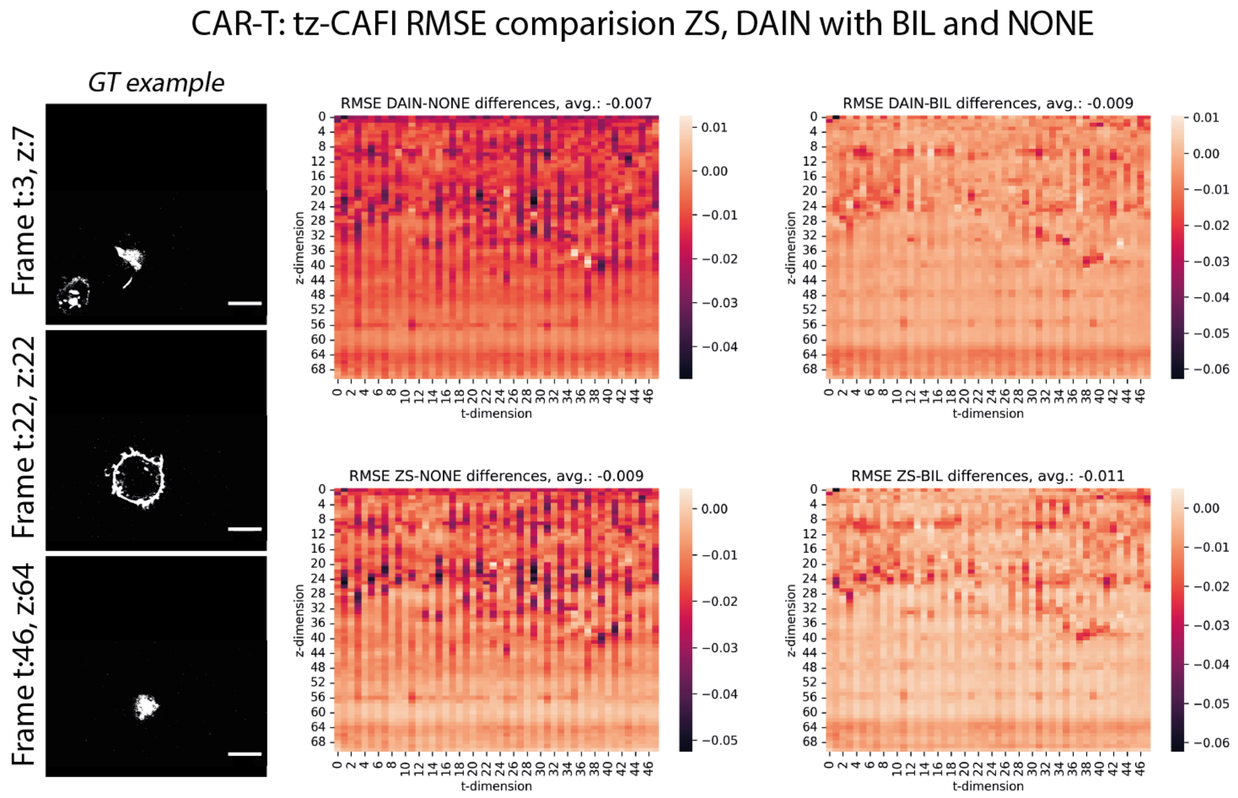

Fig. S15. Delta-RMSE quality comparison of DAIN and ZS compared to BIL and NONE of 4D CAR-T cell dataset. Every second slice in the z-dimension was removed for this visualization (GT image dimensions: 47:115:512:512). The averaged error difference was calculated based on the entire dataset of all interpolated images across both t- and z-dimensions. Left panel shows example images of sample at different time points and z-dimensions. Scale bars: 8 µm.

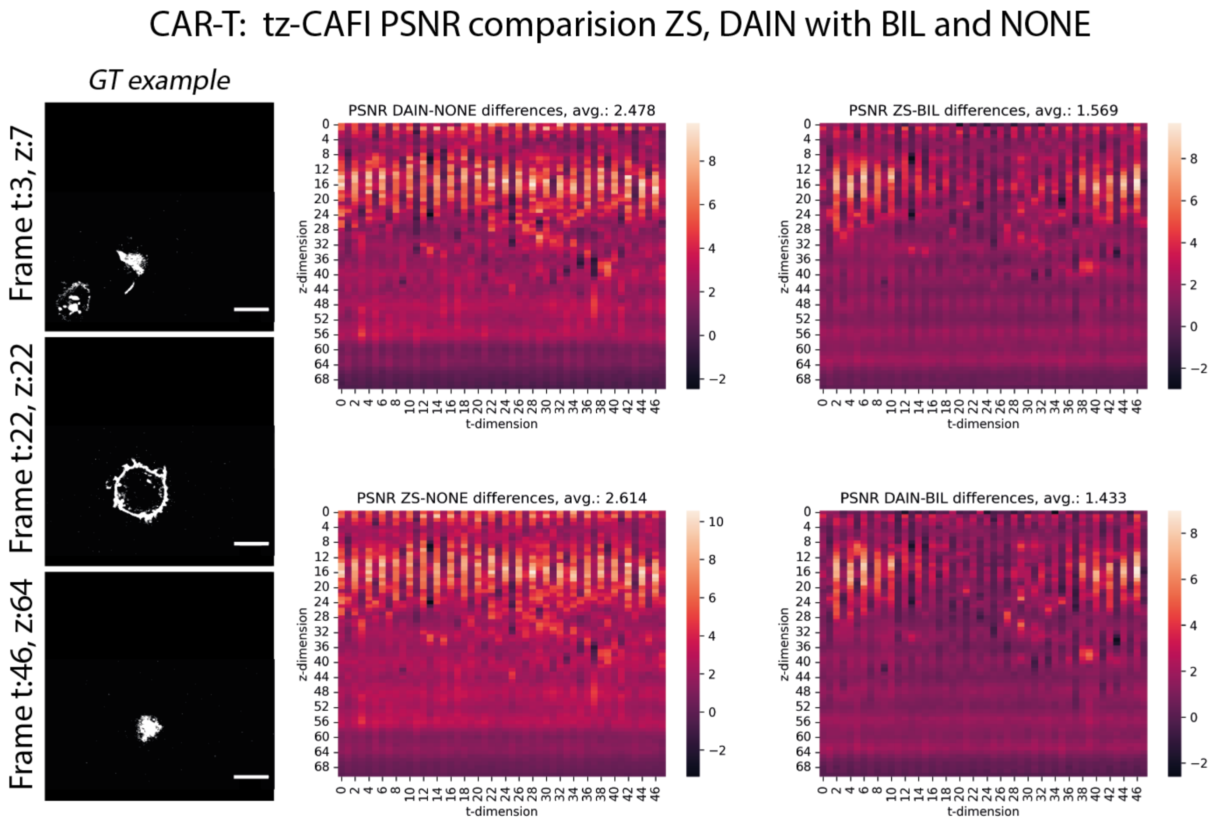

Fig. S16. Delta-PSNR quality comparison of DAIN and ZS compared to BIL and NONE of 4D CAR-T cell dataset. Every second slice in the z-dimension was removed for this visualization (GT image dimensions: 47:115:512:512). The averaged PSNR difference was calculated based on the entire dataset of all interpolated images across both t- and z-dimensions. Left panel shows example images of sample at different time points and z-dimensions. Scale bars: 8 µm.

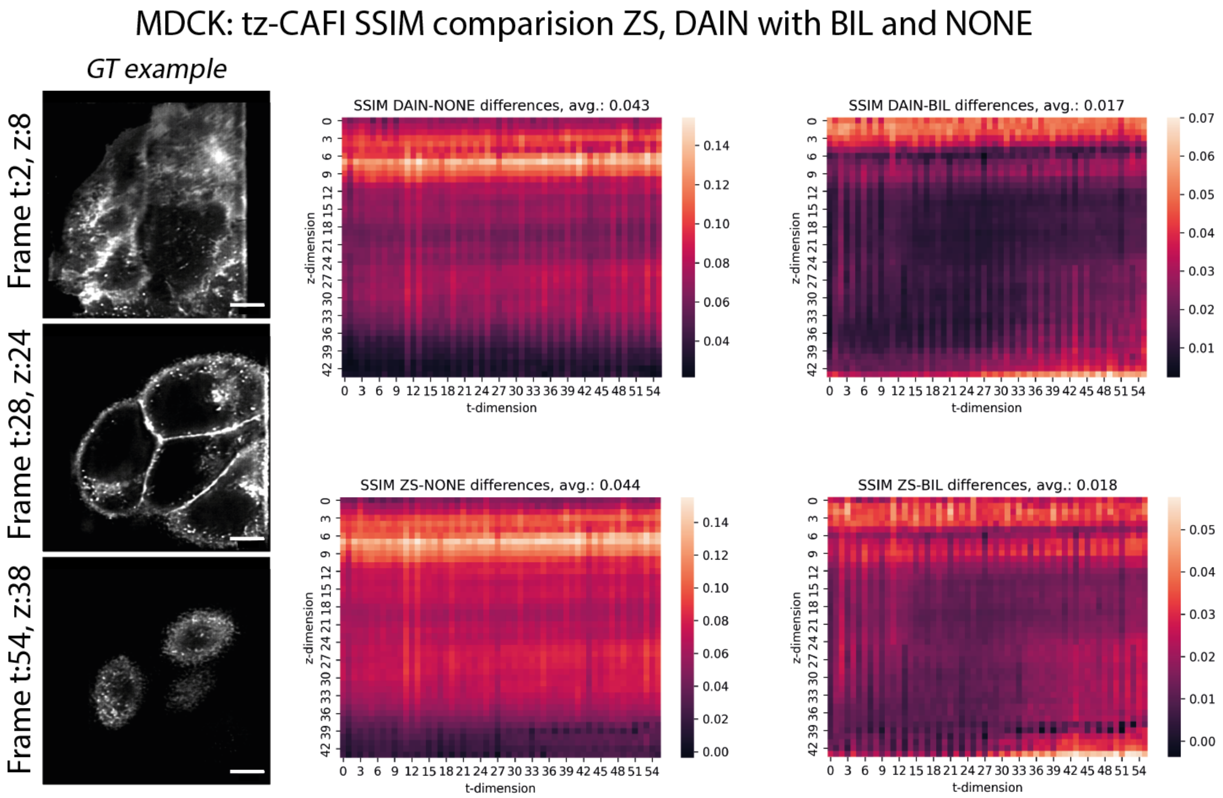

Fig. S17. Delta-SSIM quality comparison of DAIN and ZS compared to BIL and NONE of a 4D MDCK dataset. Every second slice in the z-dimension was removed for this visualization (GT image dimensions: 55:95:512:512). The averaged similarity difference was calculated based on the entire dataset of all interpolated images across both t- and z-dimensions. Left panel shows example images of sample at different time points and z-dimensions. Scale bars: 8 µm.

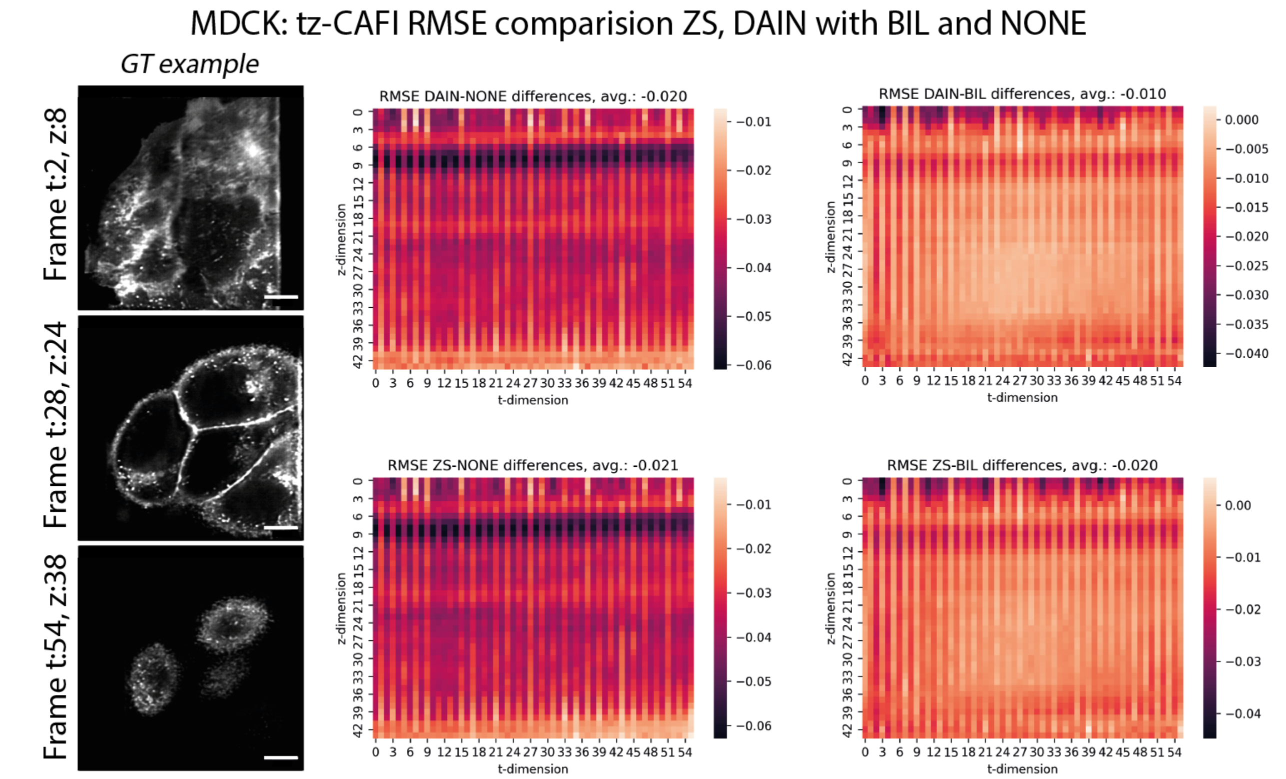

Fig. S18. Delta-RMSE quality comparison of DAIN and ZS compared to BIL and NONE of a 4D MDCK dataset. Every second slice in the z-dimension was removed for this visualization (GT image dimensions: 55:95:512:512). The averaged error difference was calculated based on the entire dataset of all interpolated images across both t- and z-dimensions. Left panel shows example images of sample at different time points and z-dimensions. Scale bars: 8 µm.

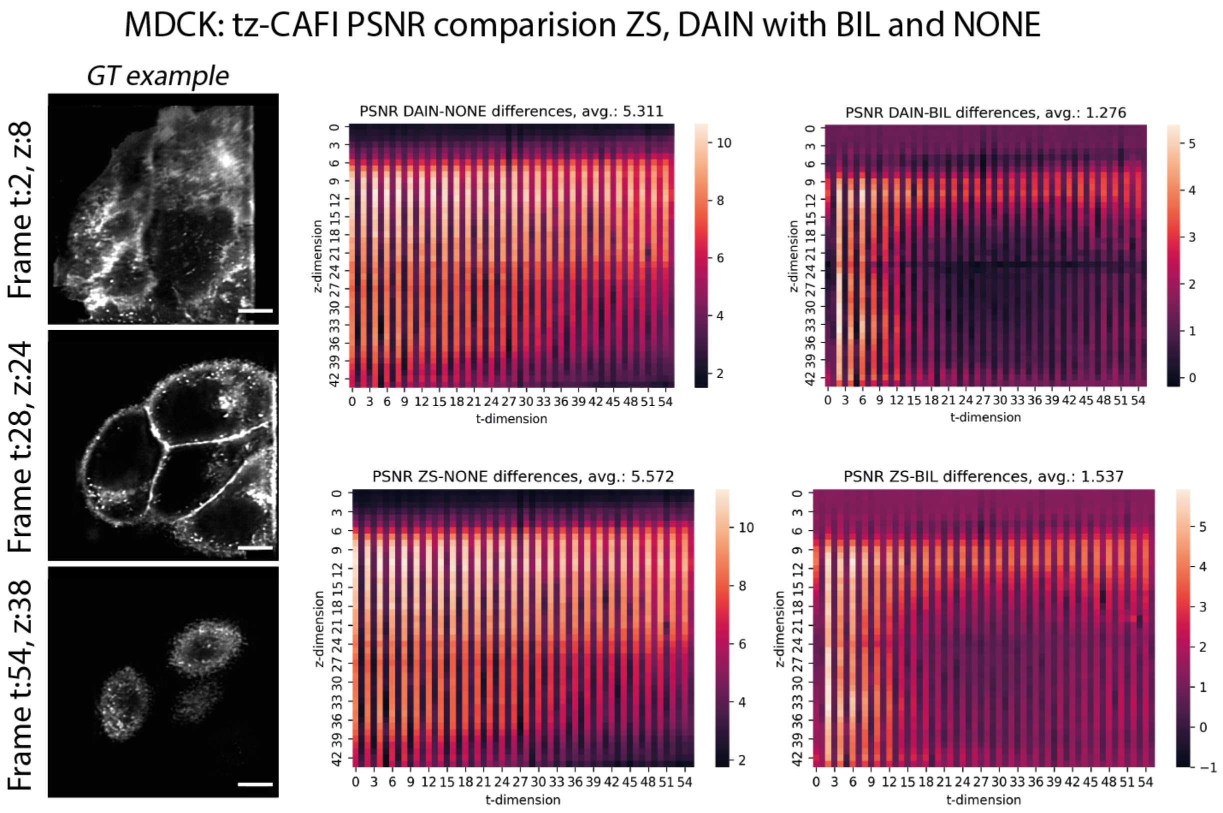

Fig. S19. Delta-PSNR quality comparison of DAIN and ZS compared to BIL and NONE of a 4D MDCK dataset. Every second slice in the z-dimension was removed for this visualization (GT image dimensions: 55:95:512:512). The averaged PSNR difference was calculated based on the entire dataset of all interpolated images across both t- and z-dimensions. Left panel shows example images of sample at different time points and z-dimensions. Scale bars: 8 µm.

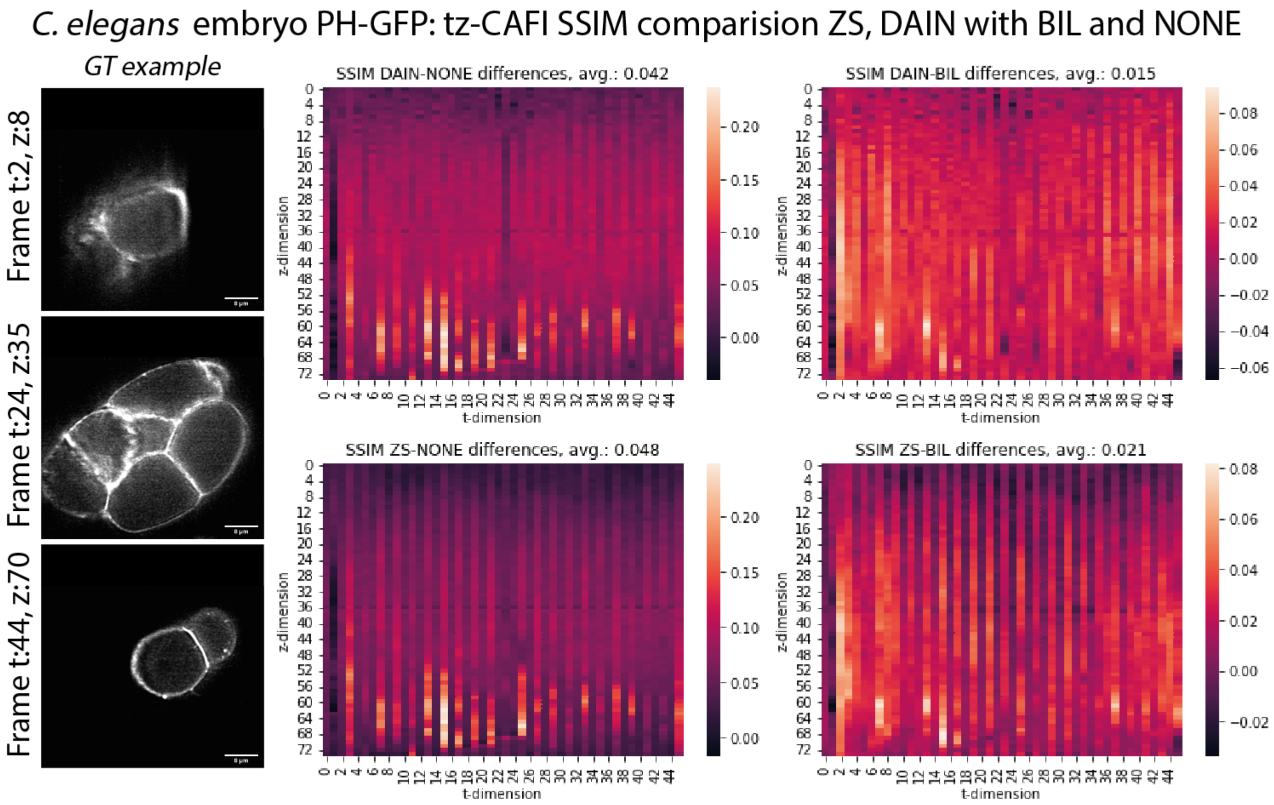

Fig. S20. Delta-SSIM quality comparison of DAIN and ZS compared to BIL and NONE of a 4D *C. elegans* PH-GFP dataset. Every second slice in the z-dimension was removed for this visualization (GT image dimensions: 45:149:512:512). The averaged similarity difference was calculated based on the entire dataset of all interpolated images across both t- and z-dimensions. Left panel shows example images of sample at different time points and z-dimensions.

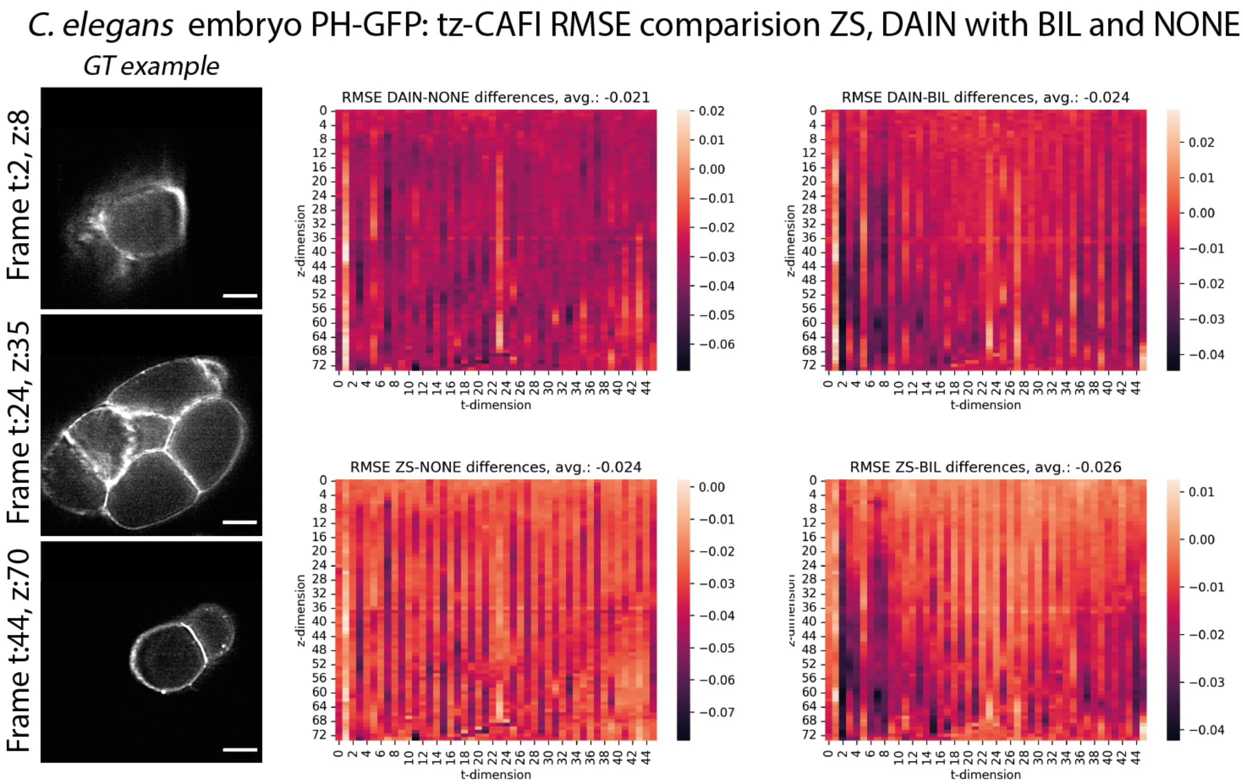

Fig. S21. Delta-RMSE quality comparison of DAIN and ZS compared to BIL and NONE of a 4D *C. elegans* PH-GFP dataset. Every second slice in the z-dimension was removed for this visualization (GT image dimensions: 45:149:512:512). The averaged error difference was calculated based on the entire dataset of all interpolated images across both t- and z-dimensions. Left panel shows example images of sample at different time points and z-dimensions. Scale bars: 8 µm.

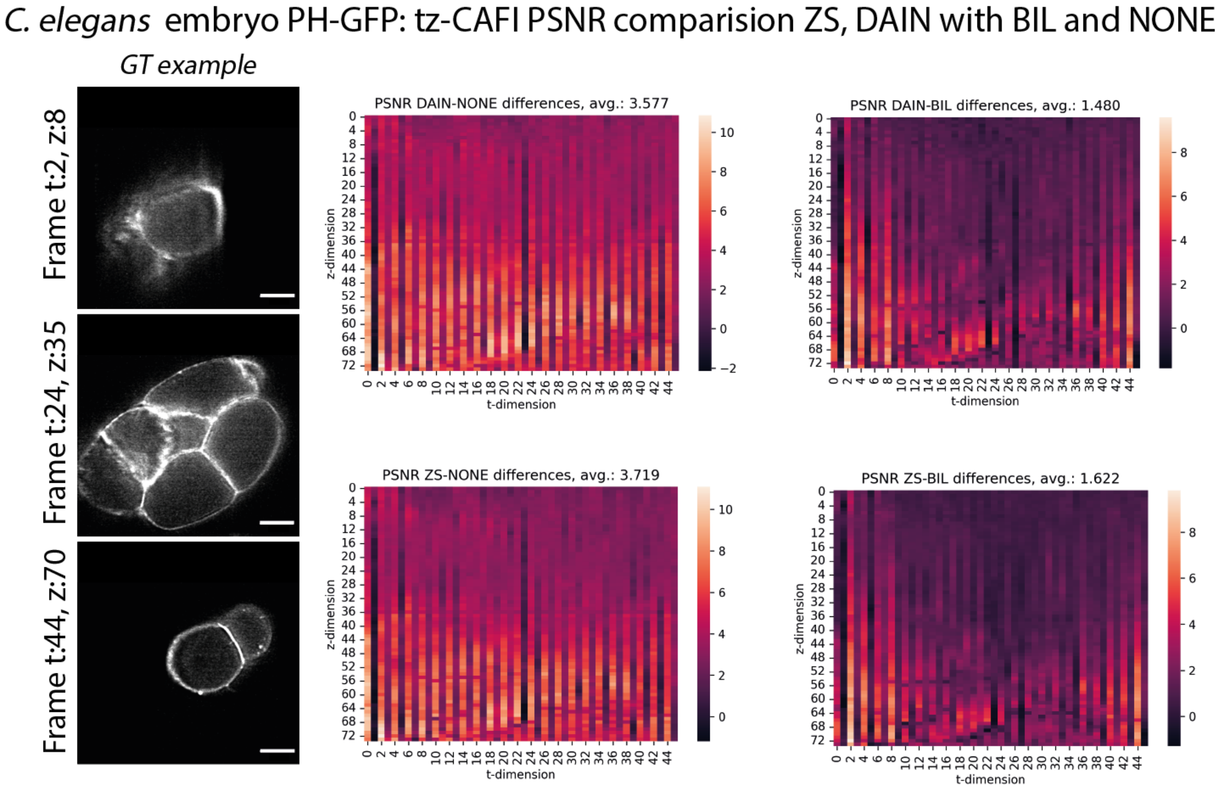

Fig. S22. Delta-PSNR quality comparison of DAIN and ZS compared to BIL and NONE of a 4D *C. elegans* PH-GFP dataset. Every second slice in the z-dimension was removed for this visualization (GT image dimensions: 45:149:512:512). The averaged PSNR difference was calculated based on the entire dataset of all interpolated images across both t- and z-dimensions. Left panel shows example images of sample at different time points and z-dimensions. Scale bars: 8 µm.

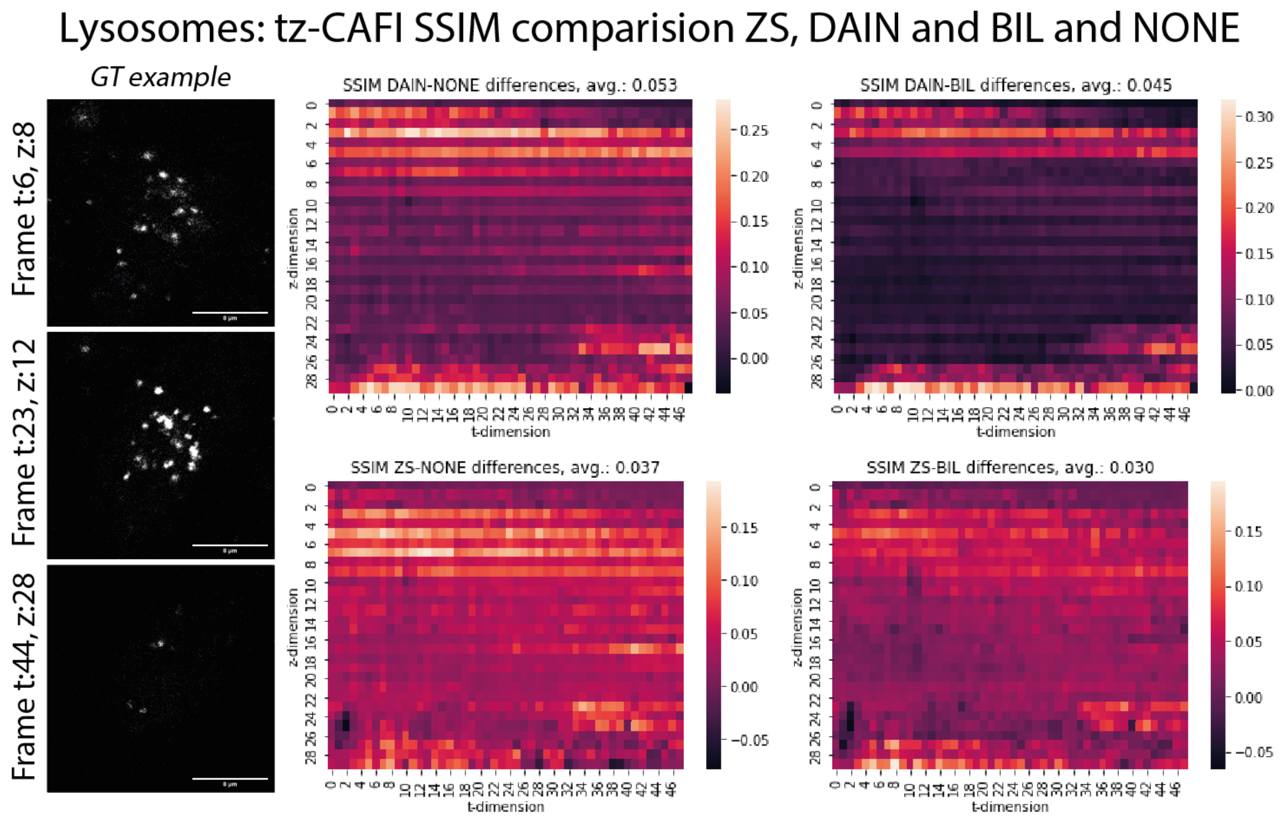

Fig. S23. Delta-SSIM quality comparison of DAIN and ZS compared to BIL and NONE of a 4D lysosomal dataset. Every second slice in the t-dimension was removed for this visualization. (GT image dimensions: 97:31:512:512). The averaged similarity difference was calculated based on the entire dataset of all interpolated images across both t- and z-dimensions. Left panel shows example images of sample at different time points and z-dimensions. Scale bars: 8 µm.

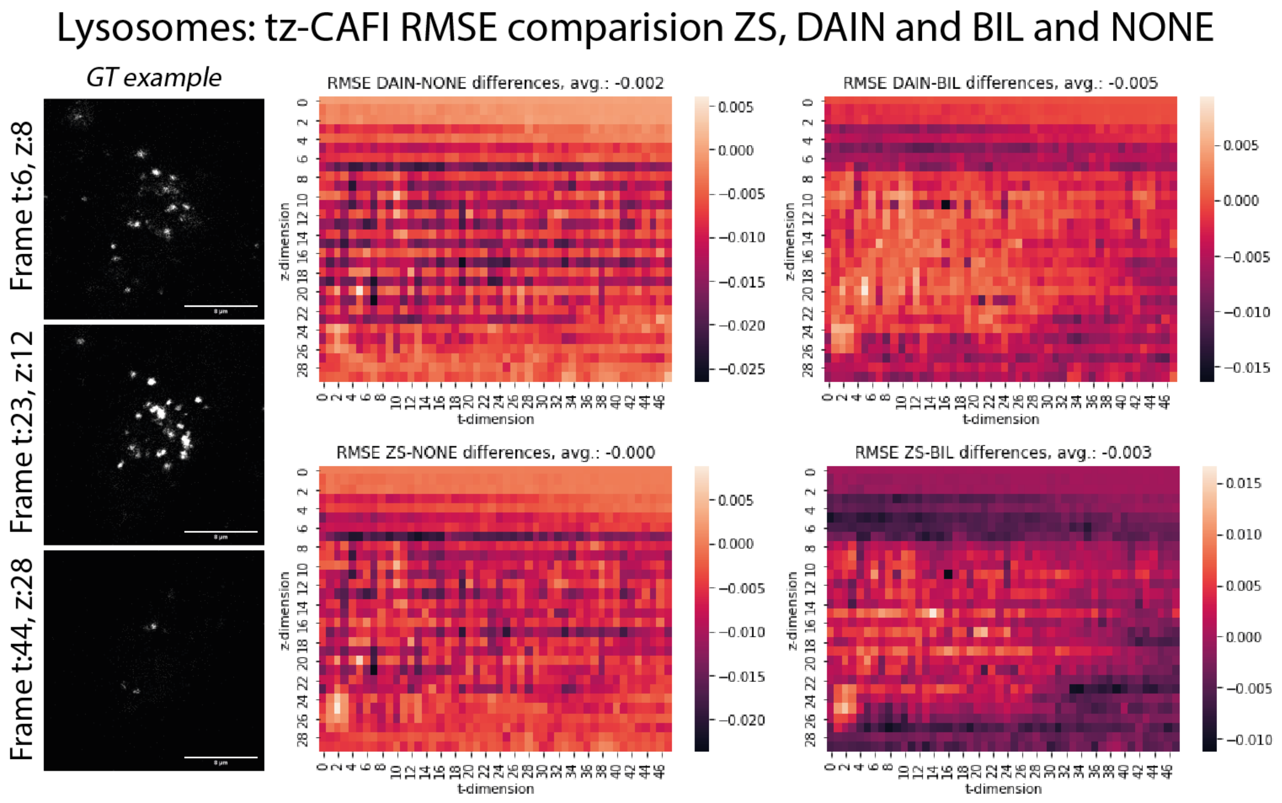

Fig. S24. Delta-RMSE quality comparison of DAIN and ZS compared to BIL and NONE of a 4D lysosomal dataset. Every second slice in the t-dimension was removed for this visualization (GT image dimensions: 97:31:512:512). The averaged error difference was calculated based on the entire dataset of all interpolated images across both t- and z-dimensions. Left panel shows example images of sample at different time points and z-dimensions. Scale bars: 8 µm.

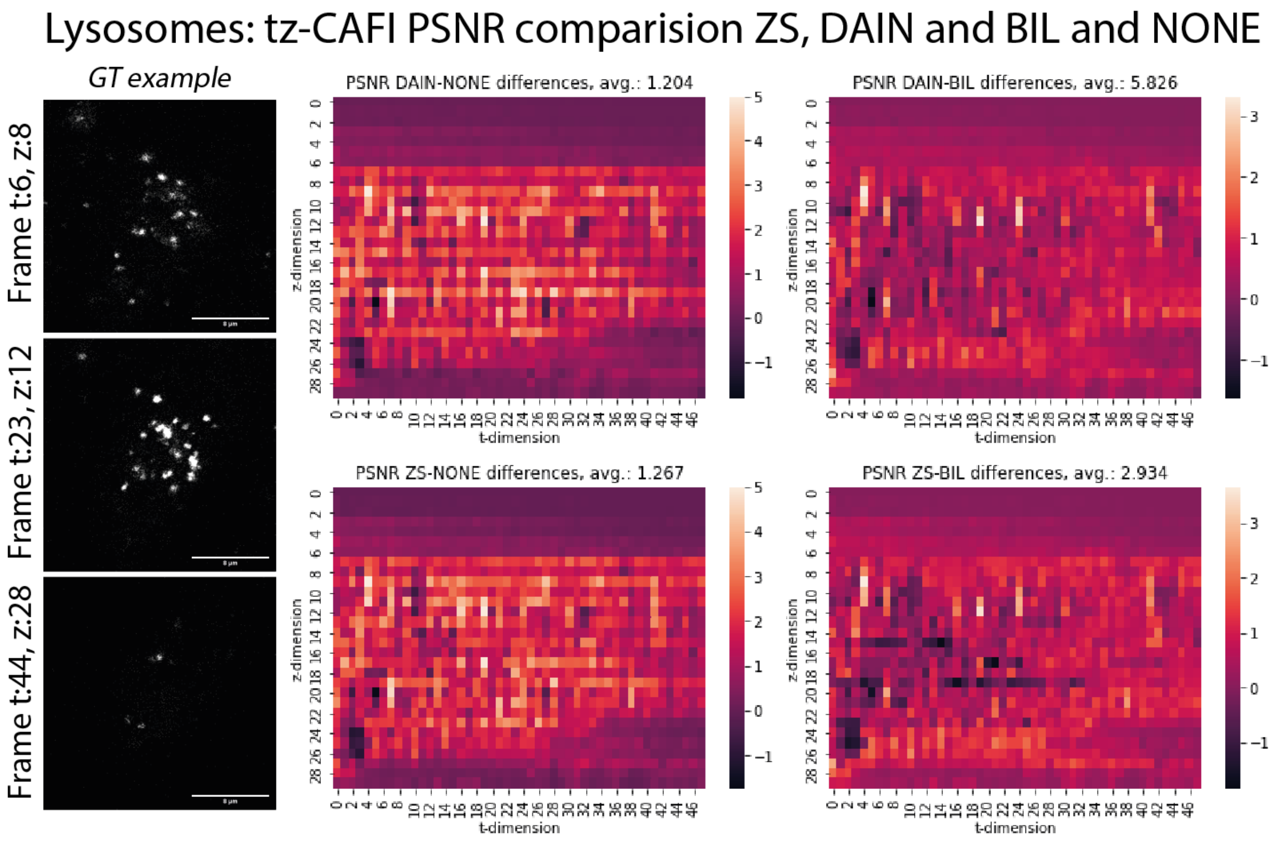

Fig. S25. Delta-PSNR quality comparison of DAIN and ZS compared to BIL and NONE of a 4D lysosomal dataset. Every second slice in the t-dimension was removed for this visualization (GT image dimensions: 97:31:512:512). The averaged PSNR difference was calculated based on the entire dataset of all interpolated images across both t- and z-dimensions. Left panel shows example images of sample at different time points and z-dimensions. Scale bars: 8 µm.

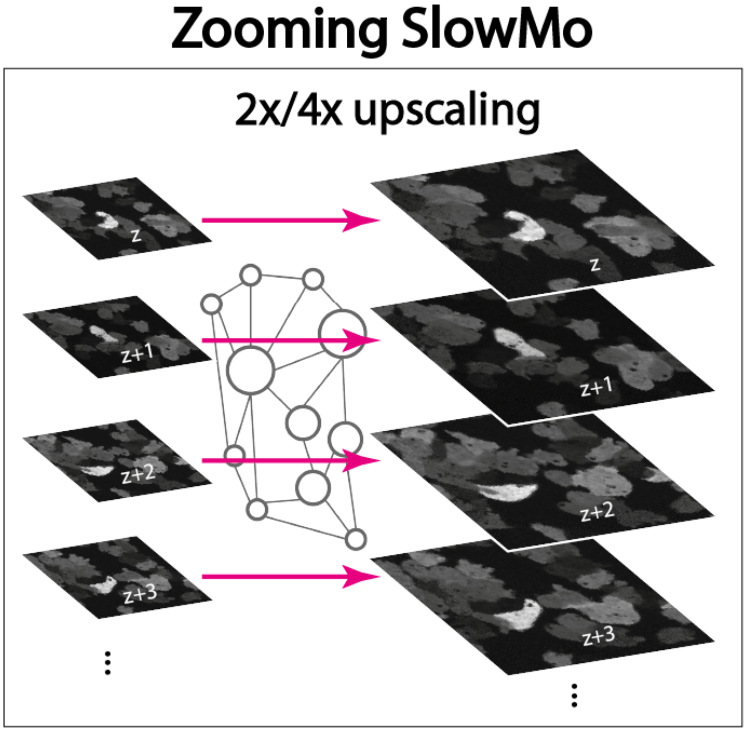

Fig. S26. Schematic representation of 2x or 4x lateral upsampling functionality of Zooming SlowMo (ZS). This functionality of ZS was compared for four different datasets by comparing it with BIC, BIL upscaling and two neural network solutions PSSR (30) and SRFBN-S (41).

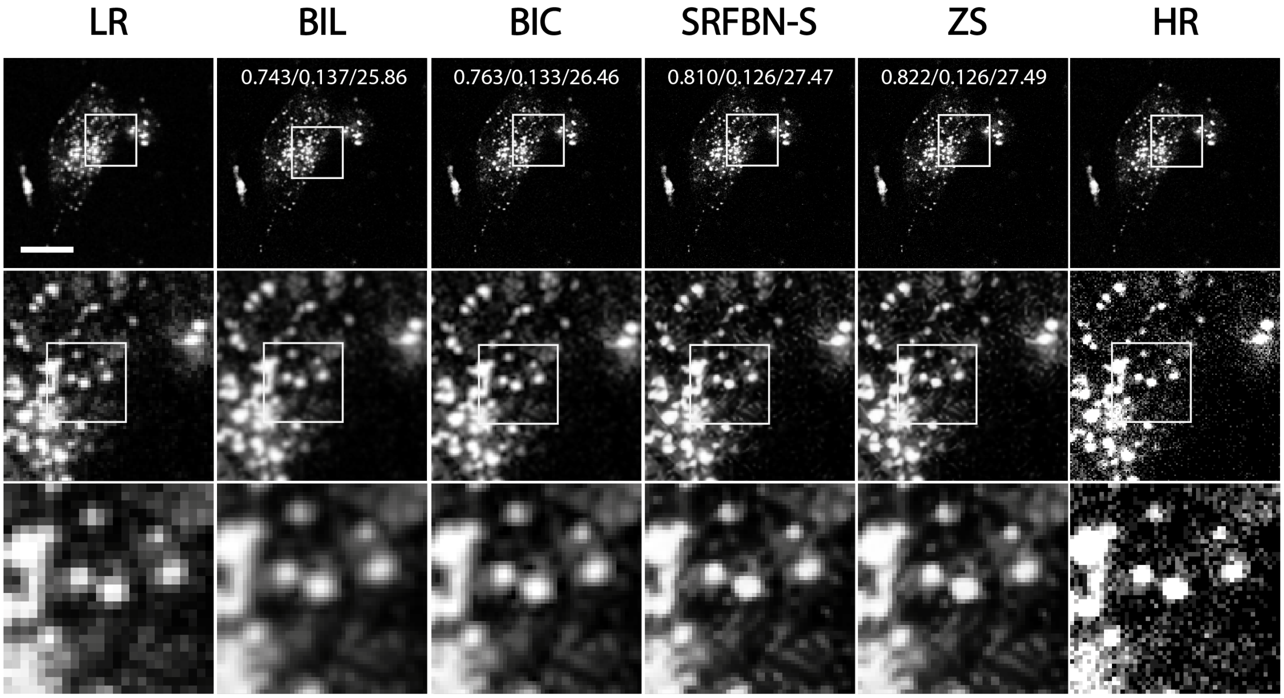

Fig. S27. 2x lateral upsampling of lysosomes of SH-SY5Y cells recorded on a point-scanning confocal microscope (256 px to 512 px). SSIM/RMSE/PSNR values displayed in the first row of upsampled images pertain to this specific image but are representative for the entire data sample of (11 number of images). Metrics for other slices can be found in the accompanying raw source data file. Scale bar: 15 µm.

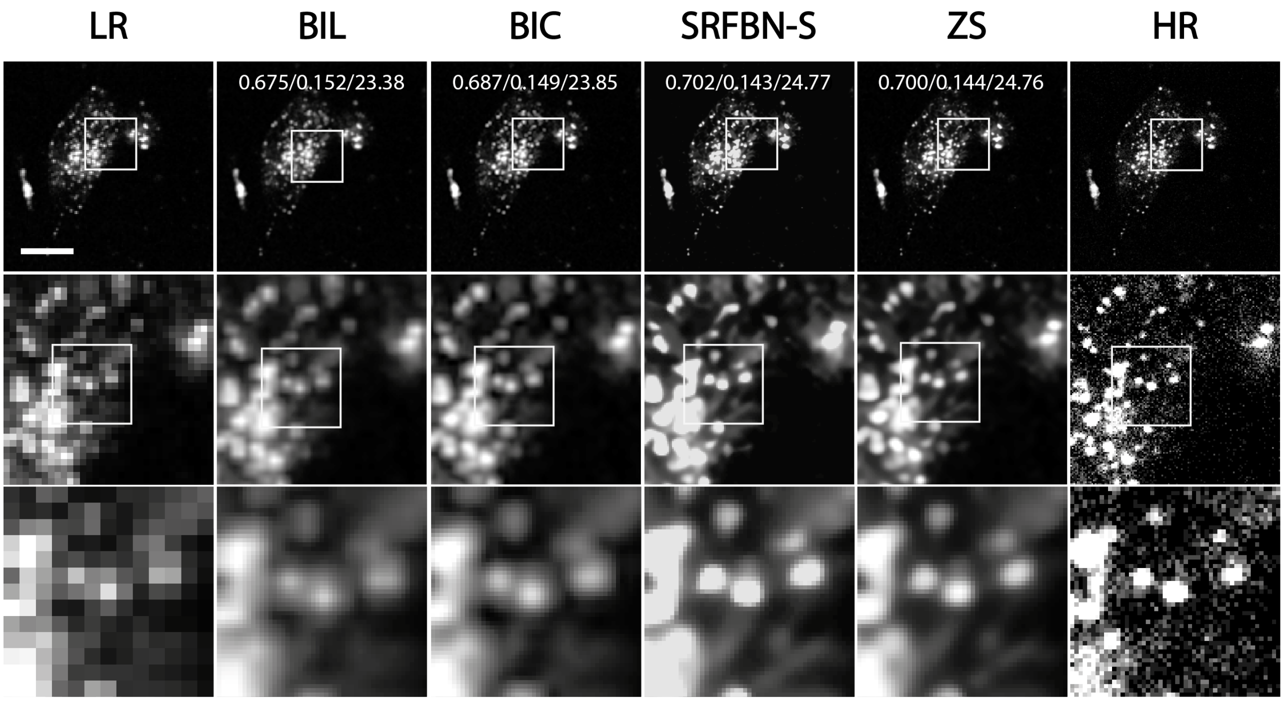

Fig. S28. 4x lateral upsampling of lysosomes of SH-SY5Y cells recorded on a point-scanning confocal microscope (128 px to 512 px). SSIM/RMSE/PSNR values displayed in the first row of upsampled images pertain to this specific image but are representative for the entire data sample of (11 number of images). Metrics for other slices can be found in the accompanying raw source data file. Scale bar: 15 µm.

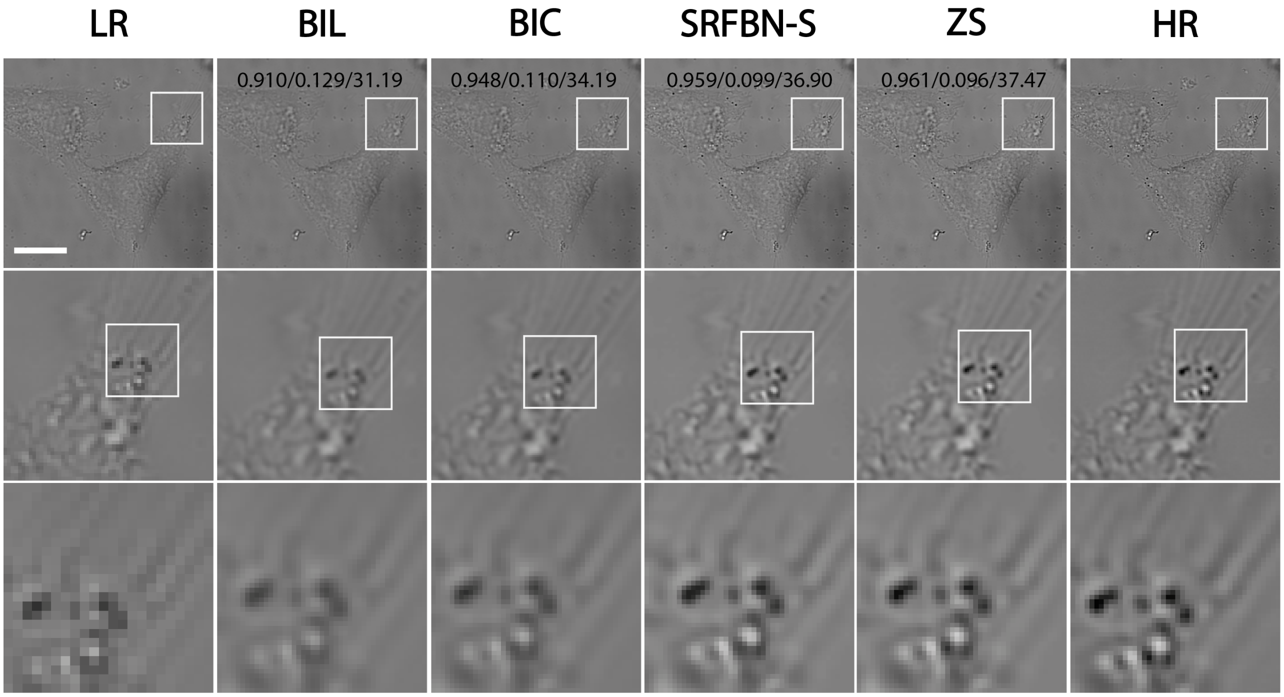

Fig. S29. 2x lateral upsampling of SH-SY5Y cells recorded on a confocal brightfield microscope (256 px to 512 px). SSIM/RMSE/PSNR values displayed in the first row of upsampled images pertain to this specific image but are representative for the entire data sample of (11 number of images). Metrics for other slices can be found in the accompanying raw source data file. Scale bar: 15 µm.

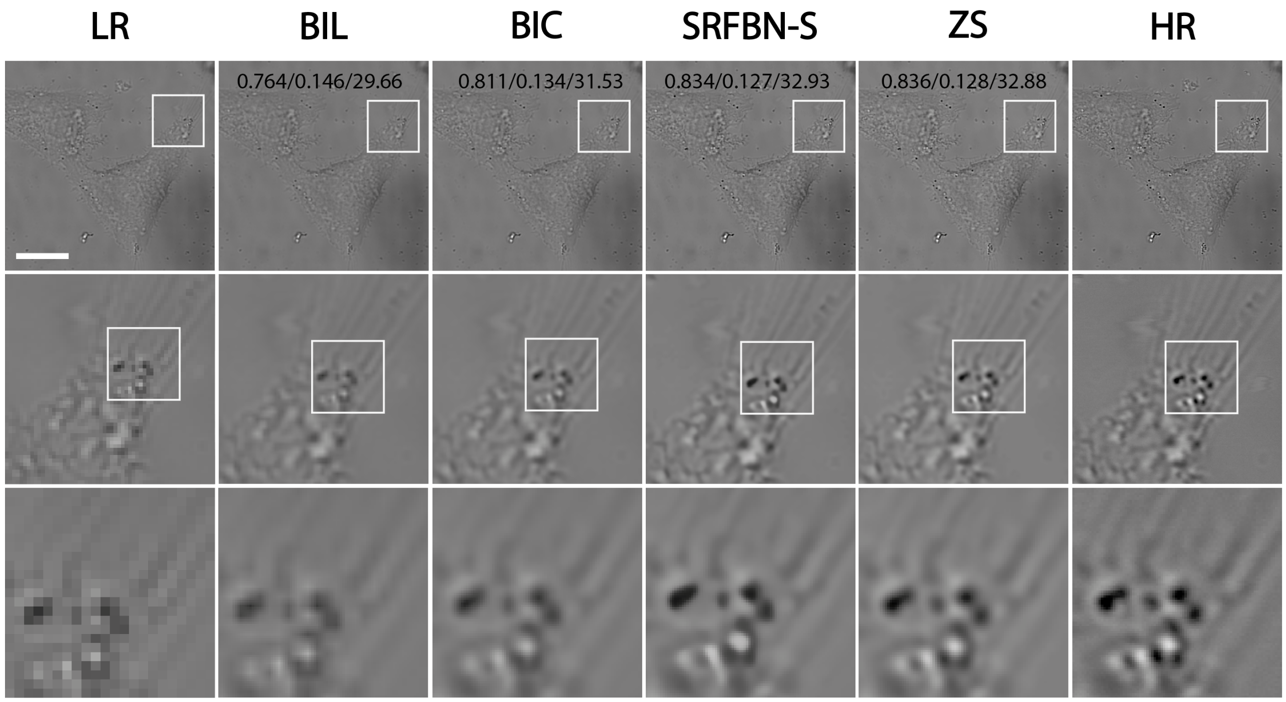

Fig. S30. 4x lateral upsampling of SH-SY5Y cells recorded on a confocal brightfield microscope (256 px to 1024 px). SSIM/RMSE/PSNR values displayed in the first row of upsampled images pertain to this specific image but are representative for the entire data sample of (11 number of images). Metrics for other slices can be found in the accompanying raw source data file. Scale bar: 15 µm.

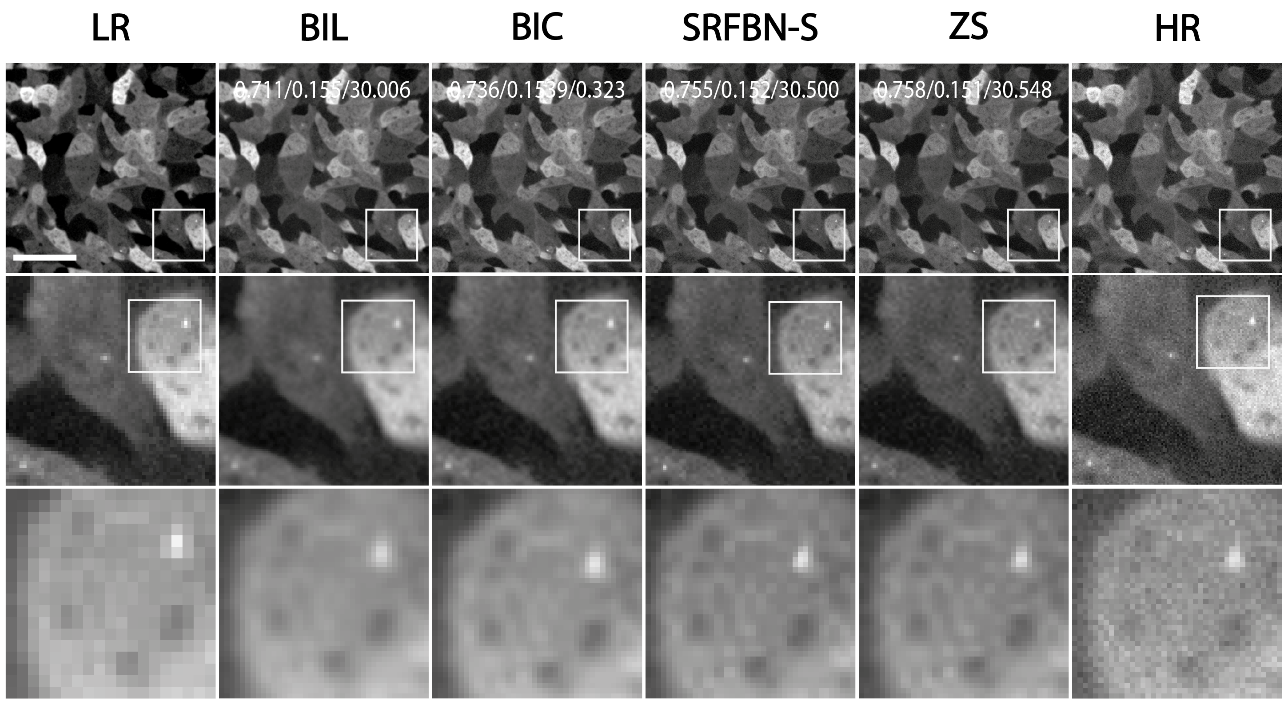

Fig. S31. 2x lateral upsampling of *Dictyostelium discoideum* cells recorded on a spinning-disk confocal microscope (256 px to 512 px). SSIM/RMSE/PSNR values displayed in the first row of upsampled images pertain to this specific image but are representative for the entire data sample of (13 number of images). Metrics for other slices can be found in the accompanying raw source data file.Scale bar: 12 µm.

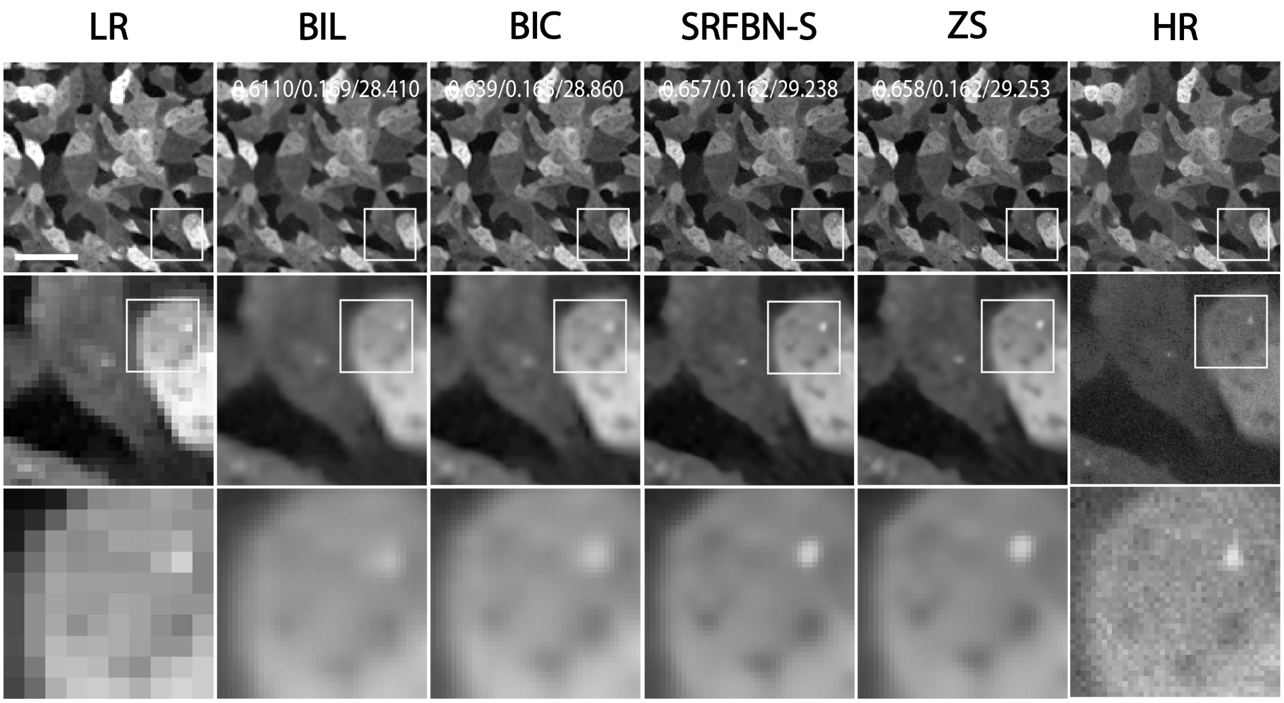

Fig. S32. 4x lateral upsampling of *Dictyostelium discoideum* cells recorded on a spinning-disk confocal microscope (128 px to 512 px). SSIM/RMSE/PSNR values displayed in the first row of upsampled images pertain to this specific image but are representative for the entire data sample of (13 number of images). Metrics for other slices can be found in the accompanying raw source data file. Scale bar: 12 µm.

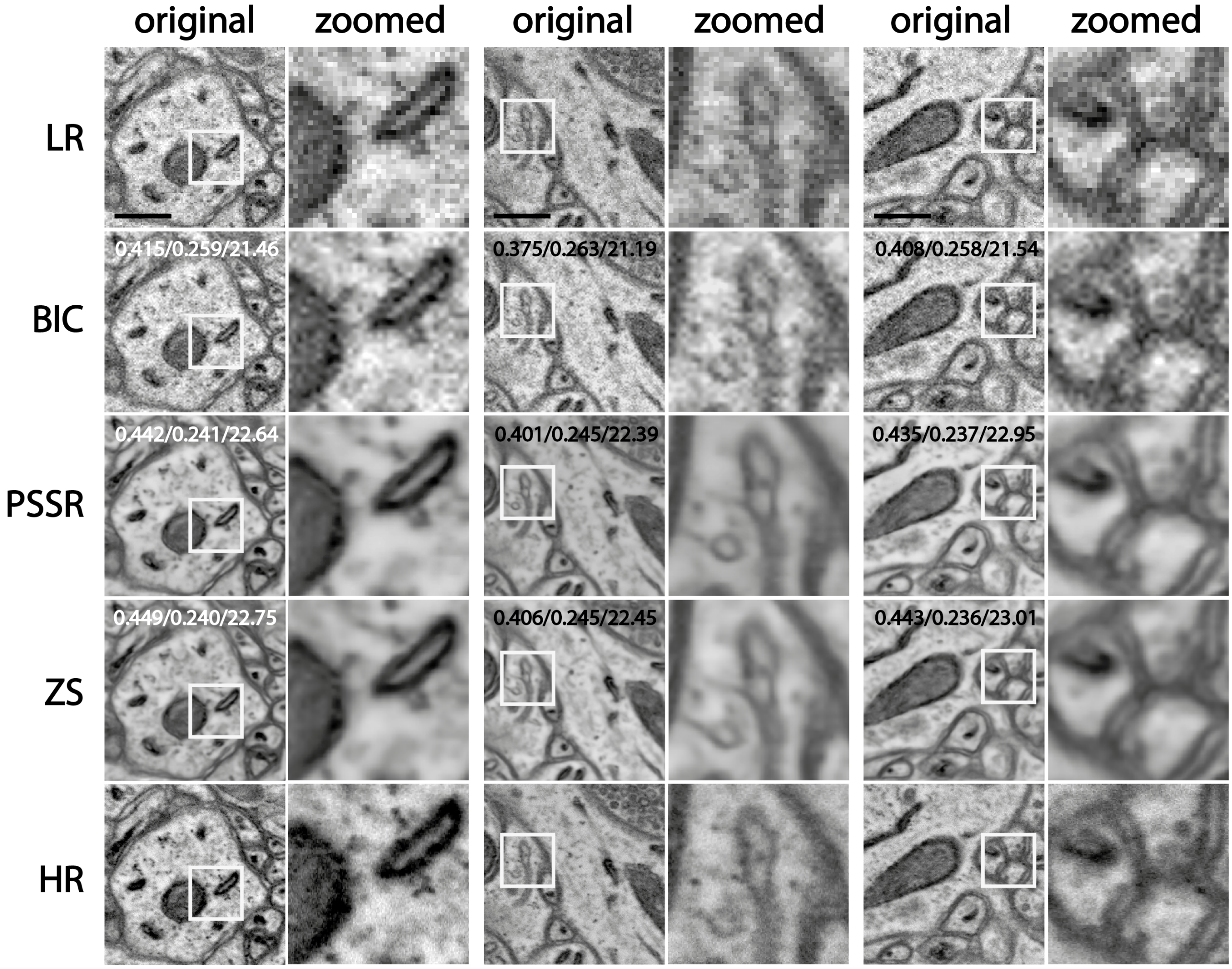

Fig. S33. 4x lateral upsampling comparison. Three representative noisy electron microscopy image examples with 4x lateral upsampling results of BIC, PSSR and ZS compared to low (LR) and high (HR) resolution images. Right image part shows the zoomed-in section highlighted with white box in the original image. Quality metrics (SSIM, RMSE, PSNR) are presented in the original lateral upsampling image of each category pertain to this specific image but are representative for the entire data sample of (42 number of images). Metrics for other slices can be found in the accompanying raw source data file. Scale bars: 0.4 µm. Data from Fang *et al.* (30).

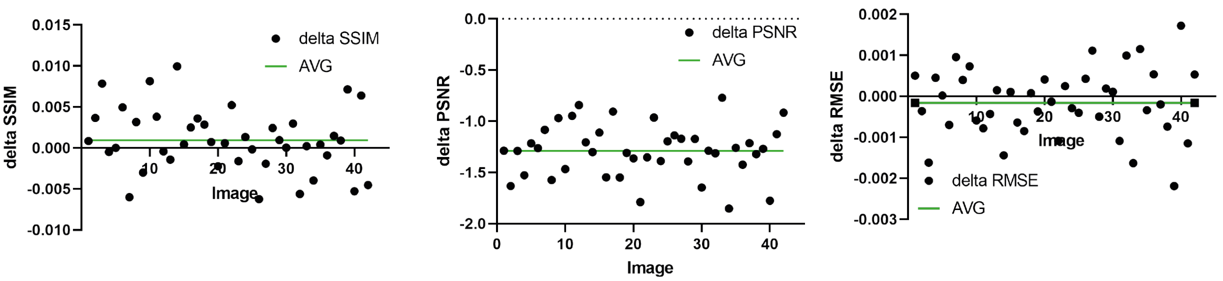

Fig. S34. Quality evaluation metrics comparison of all provided 4x lateral upsampling images of PSSR network in comparison to ZS upsampling. Delta SSIM, delta PSNR and delta RMSE are the differences of the three evaluation metrics by subtracting the evaluated image metric value of PSSR from the metric value of the same image achieved by ZS lateral upsampling.
